# Chemical and behavioural strategies along the spectrum of host specificity in ant-associated silverfish

**DOI:** 10.1186/s40850-022-00118-9

**Published:** 2022-05-11

**Authors:** T. Parmentier, M. Gaju-Ricart, T. Wenseleers, R. Molero-Baltanás

**Affiliations:** 1 Research Unit of Environmental and Evolutionary Biology, Namur Institute of Complex Systems, and Institute of Life, Earth, and the Environment, University of Namur, Rue de Bruxelles 61, B-5000 Namur, Belgium; 2 Terrestrial Ecology Unit (TEREC), Department of Biology, Ghent University, K.L. Ledeganckstraat 35, B-9000 Ghent, Belgium; 3 Depto. de Biología Animal (Zoología), University of Córdoba, E-14005 Córdoba, Spain; 4 Laboratory of Socioecology and Socioevolution, KU Leuven, Naamsestraat 59, B-3000 Leuven, Belgium

**Keywords:** Host specialization, Hydrocarbon - inquiline, *Messor* - myrmecophily, Specialization, Symbiont

## Abstract

**Background:**

Host range is a fundamental trait to understand the ecological and evolutionary dynamics of symbionts. Increasing host specificity is expected to be accompanied with specialization in different symbiont traits. We tested this specificity-specialization association in a large group of 16 ant-associated silverfish species by linking their level of host specificity to their degree of behavioural integration into the colony and to their accuracy of chemically imitating the host’s recognition system, i.e. the cuticular hydrocarbon (CHC) profile.

**Results:**

As expected, facultative associates and host generalists (targeting multiple unrelated ants) tend to avoid the host, whereas host-specialists (typically restricted to *Messor* ants) were bolder, approached the host and allowed inspection. Generalists and host specialists regularly followed a host worker, unlike the other silverfish. Host aggression was extremely high toward non-ant-associated silverfish and modest to low in ant-associated groups. Surprisingly, the degree of chemical deception was not linked to host specificity as most silverfish, including facultative ant associates, imitated the host’s CHC profile. *Messor* specialists retained the same CHC profile as the host after moulting, in contrast to a host generalist, suggesting an active production of the cues (chemical mimicry). Host generalist and facultative associates flexibly copied the highly different CHC profiles of alternative host species, pointing at passive acquisition (chemical camouflage) of the host’s odour.

**Conclusions:**

Overall, we found that behaviour that seems to facilitate the integration in the host colony was more pronounced in host specialist silverfish. Chemical deception, however, was employed by all ant-associated species, irrespective of their degree of host specificity.

**Supplementary Information:**

The online version contains supplementary material available at 10.1186/s40850-022-00118-9.

## Background

Host specificity is a fundamental characteristic in symbiont communities. It falls along a continuum with, at one end, host-specific symbiont species that target one or few narrowly related hosts and, at the other end, host-generalist species that engage with many, unrelated hosts [1]. It is expected that symbiont species may co-exist by a trade-off between host range and the average fitness achieved on different host species. This “jack of all trades is master of none” model predicts that generalist species may associate with different host species, but have a lower average fitness on the hosts shared with specialists [2].

Macrosymbionts employ a wide range of behavioural, chemical, acoustical and morphological tactics to approach, attract, manipulate and even deceive their host [3, 4]. There has been a strong focus on the chemical deception strategies of very specialized parasites, especially those targeting Hymenoptera. Several studies showed that these symbionts may hack the chemical signalling system of the host. A spectacular example can be found in a blister beetle, whose larvae lure and climb on the male of their solitary bee host by mimicking the sex pheromone of the female bee. Eventually, they pass on to the female during mating and are transported to the nest [5]. By contrast, behavioural strategies of symbionts that facilitate the association with the host have been poorly studied so far. However, more and more it appears that symbiont behaviour plays a pivotal role, either on its own or in combination with other tactics, for a successful host association of host specialists and generalists [6, 7]. Studying different tactics simultaneously in host symbiont communities may hint at how symbionts employ a range of different tactics concurrently and whether the relative importance of the tactics changes with increasing host specificity.

It has been argued that increasing host specificity within symbiont clades is accompanied by specialization [8, 9]. The specific association of organisms with a particular host environment imposes selection for specialization of traits [9]. This link between host specificity and trait specialization has mainly been studied in insect herbivores [10], but some studies tested this association in symbiont lineages as well [9]. For example, the attachment structures in monogenean parasites are more specific and specialized with increasing host specificity [11]. Another striking example is oviposition site selection of parasitoid wasps, with endoparasitoids being more host-specific than ectoparasitoids [12].

A remarkable gradation of host specificity can be found in the different groups of arthropods associated with ants. Some species of these ant guests or myrmecophiles only target a single ant species or genus, whereas, at the other extreme, some may associate with all ant species in their distribution range [13]. In line with the positive association between host specificity in other symbiont systems [11, 12], high host specificity in myrmecophiles is often associated with increased specialization in chemical ecology, behaviour and morphology [14–17]. This increased trait specialization enables them to approach their specific host and intimately interact with them (integrated myrmecophiles, see [18]). The degree of myrmecophile specialization is most prominent in the variety of chemical and behavioural strategies. Ant colonies have an intricate nestmate recognition system, which is based primarily on a blend of cuticular hydrocarbons which are mixed among nestmates to create a Gestalt odour [19]. This colony odour has a heritable component, but also environmental (e.g. diet) and social drivers (monogyny vs polygyny, nest isolation) have been identified [20–23]. Ants tend to reject or attack individuals when their odour deviates from the familiar colony odour [19]. Many myrmecophiles dupe the host by chemical disguise of the nest’s odour, which enables them to stay undetected in the nest. They either actively produce (chemical mimicry) or passively acquire the chemical odour (chemical camouflage) of their host colony ([24], see Table 1 in [25]). Active production of hydrocarbons is often found in well-integrated myrmecophiles with a narrow host range, whereas passive acquisition is more flexible and allows host species switching [24–26]. An alternative form of chemical disguise is when ant associates suppress the concentration of hydrocarbons below detectability for the host. This makes them odourless (chemical insignificance) and thus virtually undetectable [24]. Chemical insignificance on its own is probably a general strategy employed by non-integrated (low trait specialization, and tend to avoid host interaction, sensu Kistner 1979 [18]) social insect associates with a broad host range [25, 27–29]. Crucially, cuticular deception strategies are not mutually exclusive and often used in concert. Several studies showed that social insect parasites make first use of insignificance or chemical mimicry to approach and invade the host colony. Once integrated, they finetune the chemical deception by passively acquiring the host-colony specific cues [24, 30, 31]. Highly integrated myrmecophiles often secrete substances that manipulate the behaviour of the ant host in concert with chemical deception [32]. They also show advanced behaviour to facilitate their integration in the host colony. Their behavioural repertoire consists of approaching the host, climbing on the host worker, allowing inspection, exchanging of food and grooming with workers [15, 16, 33]. By contrast, the chemical and behavioural strategies of a large group of non-integrated myrmecophiles lack adaptations to their cuticular profile. They resemble their free-living relatives and are detected as intruders [33]. Ants display aggressive behaviour towards them, but they might survive the hostile nest environment by displaying unspecialized behaviour, such as fleeing, hiding, ducking, feigning death or emitting repellent chemicals [7]. Non-integrated species often have a broader host range than integrated species [15, 16], although some of these non-integrated species tend to have a narrow host range [13, 34]. These species are probably attracted to particular nest conditions or food sources only found in nests of related ants (e.g., myrmecophiles in organic mounds of *Formica* ants [35], carton nest in *Lasius fuliginosus* [36]).

A large number of silverfish (order Zygentoma) within the families Nicoletiidae and Lepismatidae made the transition from free-living soil dwellers to facultative guests of ant and termite nests and ultimately to permanent social insect associates [33, 37–40]. Silverfish are wingless, primitive insects with distinctive scales on their body and are particularly species-rich in the Iberian peninsula [40, 41]. Based on their dependency on ants, silverfish can be categorized as (1) unassociated (free-living) species, found away from ants (2) occasional or facultative myrmecophiles (occur in ant nests, but can also be found away from ants) and (3) obligate or strict myrmecophiles (always found in ant nests). The latter group spans a gradient of host specificity with species showing no specific host association, to host-specific species that are mainly restricted to the nests of a single ant genus, especially *Messor* or *Aphaenogaster* [40]. Previous research suggested the use of different chemical deception strategies in myrmecophilous silverfish with *Malayatelura ponerophila* and *Trichatelura manni* displaying chemical resemblance and two unidentified species employing chemical insignificance [28, 39, 42, 43]. In contrast to other myrmecophilous groups such as beetles and flies [33], there is apparently limited morphological divergence between host-specialist and generalist species and even to free-living relatives. The most defining morphological features are the yellow colour of most myrmecophilous species, a reduction of the length of terminal filaments and a trend to a limuloid shape, with the lateral areas of the thorax expanded [40].

Currently, few studies compared the behavioural and/or chemical integration and deception mechanisms within a single lineage of myrmecophiles with different degrees of host specificity. They either focused on two species at the extremes of the host specificity gradient (crickets in [15]) or compared host-specialized species (associated with one genus) with extreme host specialists (associated with one species, rove beetles in [16]). Our aim was to study behavioural and chemical strategies along the different stages of ant host specificity in a large group of European silverfish (species belonging to the order Zygentoma: families Nicoletiidae and Ateluridae) encompassing unassociated free-living species, facultative ant-associates, obligate ant associates with a broad host range (generalists) and host-specific species (host specialists). Furthermore, we analysed the CHC profile of freshly moulted individuals of host generalist and host specialist silverfish species that chemically mimic their host. This may help us to understand whether the CHCs were passively acquired (= chemical camouflage, moulted individuals would then lose the host profile) or actively produced (= chemical mimicry, moulted individuals would still carry the host profile even in absence of the host).

We hypothesized that facultative myrmecophiles have an idiosyncratic cuticular profile, deviating from their host and display similar behaviour than unassociated silverfish. We further predicted that obligate myrmecophilous species show different strategies, where species with broader host ranges relying on chemical insignificance, generalist species passively acquiring the host’s profile and the host specialists relying on chemical mimicry. In parallel, we predicted that unassociated and facultative species elicit high levels of aggression and display avoidance behaviour, whereas host specialist species provoke little or no aggression and tend to approach their host.

## Methods

### Study species

We studied species of ant-associated silverfish belonging to the subfamily Atelurinae of the family Nicoletiidae (2 species: *Atelura formicaria* and *Proatelurina pseudolepisma*) and to the subfamily Lepismatinae of the family Lepismatidae (14 species). Ant-associated silverfish were categorized based on the observed host associations and similar to the criteria used in [40]; i.e., facultative species (*Lepisma baetica* and *L. saccharinum*) regularly occur in absence of ants but can be found in ant nests as well. *Lepisma saccharinum* is usually a synanthropic insect and unassociated with ants in temperate Europe, but a stable population has been found living in nests of *Formica rufa* in Northern Belgium [44]. Generalist species (Atelurinae: *Atelura formicaria* and *Proatelurina pseudolepisma,* Lepismatinae: *Neoasterolepisma curtiseta*) are strictly bound to ant nests and can be found in colonies of several genera of ants. Host specialists are also strictly ant-associated and typically found with one host genus; i.e. more than 90% of the total associations registered for the species (detailed host associations see [40]). We sampled within the group of host specialists *Messor* specialists (*Neoasterolepisma balearicum*, *N. crassipes*, *N. foreli*, *N. gauthieri calvum*, *N. lusitanum*, *N. soerenseni*, *N. spectabile*, *N. wasmanni* and *Tricholepisma aureum*), *Aphaenogaster* specialists (*N. delator* and *N. hespericum*) and a *Camponotus* specialist (*T. indalicum*). All host specialists belong to the Lepismatinae subfamily and *Messor* specialists outnumber the other myrmecophilous species in southern Europe [40]. Based on morphological traits, there is strong support that *Messor* specialists form a monophyletic group within the Lepismatinae and are more derived than host generalists and *Aphaenogaster* and *Camponotus* specialist species (Additional file 1). Five species not associated with ants (Lepismatinae: *Allacrotelsa kraepelini* and Ctenolepismatinae: four *Ctenolepisma* species) were also sampled as a control for the behavioural and chemical strategies. All tested species and their respective hosts are listed in Table 1.

**Table 1:** Species of silverfish and their host ants sampled for this study

Silverfish species	Host specificity	Host ant species	Abbreviation host	Behavioural interaction tests -*N*_ind_	Survival tests -*N*_ind_ (*N*_survived_)	CHC analysis -*N*_ind_
*Neoasterolepisma balearicum*	*Messor* specialist	*Messor barbarus*	*Mess b*	–	–	3
*Neoasterolepisma crassipes*	*Messor* specialist	*Messor* sp.		–	–	3
*Neoasterolepisma foreli*	*Messor* specialist	*Messor barbarus*	*Mess b*	8	39 (39)	12
		*Messor timidus*		–	2 (2)	1
*Neoasterolepisma gauthieri calva*	*Messor* specialist	*Messor barbarus*	*Mess b*	–	–	3
*Neoasterolepisma lusitanum*	*Messor* specialist	*Messor barbarus*	*Mess b*	6	23 (23)	9
*Neoasterolepisma soerenseni*	*Messor* specialist	*Messor barbarus*	*Mess b*	7	–	–
*Neoasterolepisma spectabile*	*Messor* specialist	*Messor barbarus*	*Mess b*	13	86 (80)	41
*Neoasterolepisma wasmanni*	*Messor* specialist	*Camponotus cruentatus*	*Camp c*	–	–	5
*Tricholepisma aureum*	*Messor* specialist	*Messor* sp.	*Mes sp*	–	–	31
*Neoasterolepisma delator*	*Aphaenogaster* specialist	*Aphaenogaster senilis*	*Aphae s*	11	30 (20)	12
		*Aphaenogaster gibbosa*	*Aphae g*	–	2 (0)	2
*Neoasterolepisma hespericum*	*Aphaenogaster* specialist	*Aphaenogaster senilis*	*Aphae s*	–	1 (1)	–
*Tricholepisma indalicum*	*Camponotus* specialist	*Camponotus sylvaticus*	*Camp s*	–	–	5
*Atelura formicaria*	generalist	*Lasius niger*	*Las n*	8	–	–
		*Lasius flavus*	*Las f*	10	–	9
*Proatelurina pseudolepisma*	generalist	*Camponotus cruentatus*	*Camp c*	4	1 (0)	–
		*Camponotus micans*	*Camp m*	1	–	–
		*Iberoformica subrufa*	*Ibe s*	–	–	1
		*Lasius grandis*	*Las g*	–	1 (1)	0
		*Lasius niger complex*	*Las n*	7	1 (0)	4
		*Messor barbarus*	*Mes b*	–	1 (0)	–
		*Pheidole pallidula*	*Phei p*	8	5 (2)	–
		*Tetramorium* spp.	*Tet sp*	–	2 (0)	2
*Neoasterolepisma curtiseta*	generalist	*Aphaenogaster iberica*	*Aphae i*	–	3 (3)	1
		*Camponotus cruentatus*	*Camp c*	–	1 (1)	–
		*Camponotus micans*	*Camp m*	1	–	–
		*Camponotus pilicornis*	*Camp p*	7	17 (12)	4
		*Camponotus sylvaticus*	*Camp s*	3	1 (1)	
		*Cataglyphis hispanica*	*Cata h*	–	9 (0)	–
		*Cataglyphis rosenhaueri*	*Cata r*	5	–	–
		*Iberoformica subrufa*	*Ibe s*	5	5 (3)	1
		*Messor barbarus*	*Mess b*	–	7 (5)	–
		*Tapinoma nigerrimum*	*Tap n*	–	1 (1)	1
*Lepisma baetica*	facultative	*Messor barbarus*	*Mess b*	–	1 (0)	–
		*Pheidole pallidula*	*Phei p*	7	–	–
		*Tetramorium* spp.	*Tetr sp*	6	9 (8)	7
		unassociated		–	–	1
*Lepisma saccharinum*	unassociated/facultative	*Formica rufa*	*Form r*	11	–	4
*Allacrotelsa kraepelini*	unassociated	*–*		–	–	5
*Ctenolepisma ciliatum*	unassociated	*Camponotus cruentatus*	*Camp c*	2	–	7
		*Camponotus micans*	*Camp m*	5	–	
		*Messor barbarus*	*Mess b*	6	3 (0)	
		*Messor capitatus*	*Mess c*	1	–	
*Ctenolepisma nicoletii*	unassociated	*Aphaenogaster senilis*	*Aphae s*	–	1 (0)	8
		*Messor barbarus*	*Mess b*	–	1 (0)	
		*Pheidole pallidula*	*Phei p*	–	1 (0)	
*Ctenolepisma targionii*	unassociated	*Messor barbarus*	*Mess b*	–	1 (0)	–
*Ctenolepisma guadianicum*	unassociated	*–*		–	–	3

Host specificity based on [40]. The number of silverfish used for each type of test is indicated (*N*_ind_); The number of survived individuals in the survival tests are indicated in brackets (*N*_survived_). The number of ants used in behavioural interaction tests was always 10, the number of host ants analyzed in the chemical studies ranged from 1 to 6 in chemical studies. Unassociated silverfish were found without ants and consequently no host ants could be chemically analyzed in this group

We sampled the silverfish and ant hosts from populations in Southern and Eastern Spain, Southern France and Belgium (Additional file 2).

### Behavioural assay: ant-silverfish interaction

An extensive set of assays was conducted to study the behaviour of the silverfish and ants during interaction. As ants may kill silverfish when they interact, they were stored and transported separately to the laboratory. For these assays, 9 cm diameter plastic containers with circular plaster bottom and a fluon coated wall were used. Ten workers from the worker caste (in *Messor* 6–7 media majors were always included) were added and allowed to acclimatize for approximately 1 h. Then, one silverfish individual (coming from the same nest as workers, except for species that are not ant-associated) was introduced into the arena and, after a 20 s timeout, a video of 15 min was recorded using the camera of an iPhone XR. The number of tests performed for each pair of ant and silverfish species is listed in Table 1. Aggression behaviours of ants and the silverfish responses were scored from these videos.

Ant behaviours towards the silverfish were scored when the ant antenna crossed the body of a silverfish (= interaction). Then, we assumed that the ant was able to detect the silverfish. We identified in the response of the ants two non-aggressive interactions: ignoring (= an ant encounters a silverfish, but continues without any behavioural modification, Additional file 3: video S1) and inspection (an ant detects a silverfish and turns its head to the silverfish or antennate, Additional file 4: video S2); and three aggressive interactions: opening of the mandibles (= threat posture, an ant opens its mandibles, but does not attempt to bite, Additional file 5: video S3), biting attempt (= an ant snaps with its mandibles, but it does not touch the silverfish, Additional file 6: video S4) and effective bite (an ant touches the silverfish with its mandibles and can grasp it for some time, Additional file 7: video S5). The first 20 interactions of each test were considered. We also calculated the proportion of aggressive ant interactions versus non-aggressive interactions by dividing the sum of aggressive interactions by the total number of interactions (= 20).

**Fig. 1:**
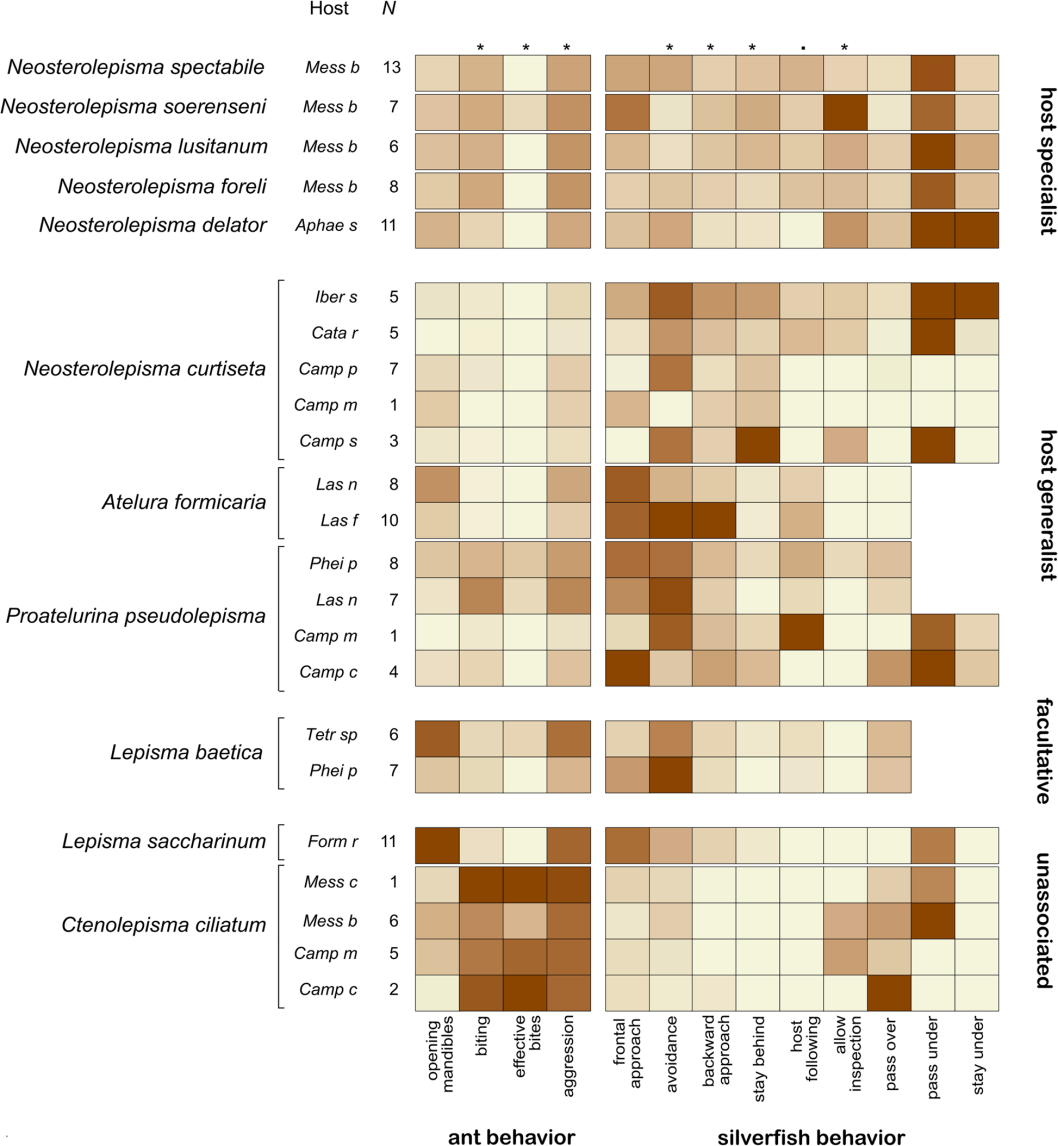
Heat map displaying the behaviour of ant hosts towards different species of silverfish (ant behaviour) and the behavioural repertoire of the silverfish (silverfish behaviour, see also Table 2). The hosts of the silverfish are abbreviated (following Table 1). Some silverfish species were associated and tested with multiple hosts. *N* number of behavioural trials per host-silverfish pair. The darkness of the colour in the heat map positively correlates with the frequency that a behaviour in a silverfish-ant pair is observed compared to other silverfish-ant pairs. Significant differences (*P* < 0.05) among the four functional groups for each behaviour are indicated with an *

The silverfish behaviours identified in the assays are defined in Table 2. Video sequences showing examples of these behaviours are presented as Additional files (Additional files 3,5,8-17: video S1, S3, S6-S15). For scoring infrequent behaviours shown by the silverfish (host following > 2 s, allowed inspection > 2 s, pass over, stay under > 2 s), the entire duration of the test was considered, but for more frequent behaviours (frontal approach, avoidance, backward approach, stay at the back > 2 s, pass under) only 4 min were chosen (3:00–5:00 and 13:00–15:00 time intervals of each video) to work efficiently. To account for cross-trial differences in ant/silverfish activity, we divided the frequency of each silverfish behaviour by the total number of ant-silverfish interactions observed in the time frame 3:00–5:00 and 13:00–15:00 (proxy for ant/silverfish activity) of the video. General differences in the behavioural repertoire between the four functional groups of silverfish (host specialist, host generalist, facultative and unassociated) were tested with a permutation test (PERMANOVA, adonis function, package vegan, [45]). The distance matrix was based on the number of times standardized silverfish behaviours shown in Table 2 were observed. Pass under and stay under were discarded as these behaviours were not possible in small host ants. Next, we tested whether the frequency of individual ant and standardized silverfish behaviours differed among the silverfish functional groups. For each behaviour, we ran a Kruskal-Wallis rank sum test with the frequency of the behaviour (23 independent observations per behaviour: trial average for each ant-silverfish pair as independent observation, see 23 rows in Fig. 1) as response variable and silverfish functional group as the exploratory variable. We controlled the false discovery rate of these multiple tests using the Benjamini-Hochberg procedure [46].

**Table 2:** The behavioural repertoire shown by the silverfish during behavioural tests. Exemplary videos can be found in the corresponding Additional files

Silverfish behaviour	Description	Time considered	Exemplary videos
**Frontal approach**	Silverfish approached the ants which stood still, and an interaction (= in reach of the antennae) occurred.	4 min	Additional file 3 (video S1)
**Avoidance**	When an ant and/or silverfish are moving they may approach frontally. Avoidance occurred when the silverfish changed its direction to avoid interaction (situations when the ant approached to the back or the side of the silverfish were discarded).	4 min	Additional file 8 (video S6) Additional file 9 (video S7)
**Backward approach**	The silverfish approached to the back of the ant, or laterally but out of the reach of the ant antennae. The distance between the silverfish and the ant is smaller than the length of the antenna of the silverfish	4 min	Additional file 10 (video S8) Additional file 11 (video S9)
**Stay at the back > 2 s**	Similar to the “approach from the back” behaviour, but the silverfish stayed more than 2 s very close to the back of a resting or slowly moving ant.	4 min	Additional file 10 (video S8) Additional file 11 (video S9)
**Host following > 2 s**	The silverfish approached a worker and persecuted it during more than 2 s. The ant was walking more or less quickly.	15 min	Additional file 12 (video S10)
**Allowed inspection > 2 s (yes/not)**	The silverfish did not move quickly when antennated by a worker	15 min	Additional file 13 (video S11) Additional file 14 (video S12)
**Pass over (yes / not)**	The silverfish walked over or tried to climb over the worker, usually very quickly	15 min	Additional file 15 (video S13)
Pass under*	The silverfish passed under the body of the ant.	4 min	Additional file 16 (video S14)
Stay under > 2 s (yes / not)*	The silverfish stayed under the body of a resting or slowly moving ant during more than 2 s.	15 min	Additional file 17 (video S15)

When the behaviour was accounted for 4 min, the time intervals were always 3:00–5:00 min and 13:00–15:00 min of the video fragment. Yes/no behaviours were scored as 1 if the behaviour occurred at least once during the 15 min of the video and as 0 if it was not observed, Frontal approach, approach from the back, avoidance, host following, allow inspection, pass over and pass under behaviour were standardized by dividing the counts by the number of interactions (= ant antenna crosses body of silverfish) observed in the trial between 3 and 5 and 13–15 min (proxy for interaction rate, considering that a higher number of these behaviours can be observed when ants and silverfish interact more)

*These behaviours were only observed and accounted in assays with ants of medium or big size. Silverfish could not pass or stay under small ants

The dataset with 142 standard tests was visually represented in a heatmap analysis where values of every ant and standardized silverfish behaviour were rescaled between 1 and 0 (where 1 is the maximum value of each behavioural act reported between all silverfish-host ant pairs and 0 is the minimum of this behavioural act reported).

### Behavioural assay: silverfish survival

As we observed that some silverfish quickly died when kept in small containers with ants, we wanted to check whether survival was dependent on the degree of host specificity. Just after their capture, silverfish and ants of the same colony were introduced together into a small transparent receptacle (5 cm diameter) and observed for 15 min to assess their survival rate (number of silverfish surviving at the end of the test / total number of silverfish introduced). The number of survival tests per silverfish species is listed in Table 1. For details of these survival tests see Additional file 18.

### Chemical analysis: protocol

Ants and silverfish were collected with hexane-cleaned forceps or glass aspirators (nests and corresponding coordinates see Additional file 2). The insects were killed by freezing and individually stored in glass vials at − 21 °C until solvent extraction and GCMS analysis. We extracted the cuticular compounds for 10 min in 2 ml vials capped with a PTFE septum (Sigma-Aldrich) in 50 μL of HPLC-grade hexane (Sigma-Aldrich) for a silverfish specimen and in 100 μL hexane for most ant workers. The largest ant workers were immersed in 200 μL of hexane. The hexane extract was transferred to another vial. Next, the solvent evaporated at room temperature in a laminar fume hood and the sample was stored at − 21 °C prior to analysis. Silverfish samples were reconstituted in 10 μL hexane and ant samples in 40 μL hexane. We injected 2 μL of each hexane extract into a Thermo GC (Trace 1300 series) coupled with a MS (ISQ series, − 70 eV, electron impact ionisation), equipped with a Restek RXi-5sil MS column (20 m × 0.18 mm × 0.18 μm) and with helium as a carrier gas at a flow rate of 0.9 mL min-1. We randomized the order of the injected samples. We selected splitless injection and held an inlet temperature of 290 °C. The oven temperature was set at 40 °C for 1 min, then followed by two temperature ramps from 40 °C to 200 °C at 20 °C min-1 and from 200 °C to 340 °C at 8 °C min-1, with a final hold of 4 min at 340 °C. We ran a C_7_ to C_40_ linear alkane ladder standard (49452-U, Supelco) at three different concentrations (0.001, 0.01 and 0.1 μg/mL) before and directly after the samples to calculate retention indices and sample concentrations. The relationship between peak area and the three tested alkane ladder concentrations was linear on a log-log scale. Therefore, we quantified the samples (amount of peak in microgram) by interpolation on a log-log scale, based on the peak areas of the closest eluting n-alkane of the external alkane ladders for each sample peak. Retention indices (Kovats indices) of all peaks were calculated using cubic spline interpolation [47] using the elution times of the aforementioned alkane ladders. Both peak quantification and retention index calculation were performed using an in-house developed R-script, available from the authors upon request. For each species, we selected the peaks that eluted between n-C20 and n-C40 and comprised on average more than 0.1% of the total peak area between n-C20 and n-C40. We identified cuticular hydrocarbons in the samples based on their mass spectra and retention indices. Double bond positions and stereoisomery were not determined.

### Chemical analysis: comparison of CHC profiles

First, we visually compared the variation in the complete CHC profiles across the entire ant-silverfish dataset (Additional file 19, total of 199 CHC peaks). There is evidence that nestmate recognition in some ants is based on only a part (Z9-alkene fraction, dimethyl fraction) of the CHC profile [48, 49], but for most ant species it is unknown which fraction contains colony-specific information. Therefore we opted to focus on the comparison of the complete ant and silverfish profile. Per sample, we standardized the mass of each cuticular hydrocarbon peak relative to the total mass of CHCs present in the sample. A Bray-Curtis similarity matrix was then calculated based on these compositional data and visualized with a non-metric multidimensional scaling plot (NMDS, R-package vegan). In parallel, we conducted a hierarchical cluster analysis (average linkage method) on the same matrix with the Bray Curtis similarities between the CHC profiles of the ants and silverfish. To avoid overloading of the cluster tree, we grouped the samples of the silverfish found with the same host species and the ant samples per species (by averaging the BC similarities in the matrix). To assess the statistical support of the clusters, we applied multiscale bootstrapping (1000 bootstraps) with the modified pvclust package for the Bray-Curtis similarity matrix. Second, we only focused on the profiles of *Messor* ants (*Messor barbarus* and *Messor timidus*) and the seven *Messor*-specialist silverfish (Table 3). The BC-matrix based on the compositional data of each sample in this *Messor - Messor* specialist subset was also visualized with a NMDS. Third, we compared the profile of each silverfish species with its host ants using different NMDS plots (BC-matrix based on compositional data). Silverfish species found in association with different ant species were compared with their different hosts in separate NMDS plots. For each host-silverfish pair, the significance of the CHC similarity between silverfish individuals and the host ant workers was tested using a PERMANOVA (adonis function in R-package vegan) based on the corresponding BC matrix of the compositional CHC peaks. Permutations were only allowed within profiles of silverfish and ants of the same nest (nest origin specified as a stratum). For each test, we ran 999 unique permutations, but less if there were too few samples to carry out this number of unique permutations (Table 3). We also conducted permutational analysis of multivariate dispersions (PERMDISP) for each host-silverfish pair to test for the homogeneity of multivariate dispersion (variation) [50].

**Table 3:** Overview of the chemical similarity to the host, chemical strategy and behaviour of the silverfish

Silverfish species	Subfamily	Host ant	N	shared CHCs (***N)***	Idiosyncratic CHCs (***N***)	BC (host BC)	P	Perm.	Host colonies	Chemical similar to host	Active production of host CHC’s	Suppression of CHCs	Behaviour
*MESSOR* SPECIALIST													
*Neoasterolepisma balearicum*	Lepismatinae	*Messor*	3	25	20	0.57 ± 0.05 (0.87 ± 0.09)	**0.049**	40	A(S145), B(S146), C(S150)	yes	?	?	–
*Neoasterolepisma crassipes*	Lepismatinae	*Messor*	3	26	11	0.71 ± 0.01 (0.87 ± 0.09)	**0.035**	56	A(S201)	yes	likely	?	–
*Neoasterolepisma foreli*	Lepismatinae	*Messor*	12	26	18	0.72 ± 0.06 (0.87 ± 0.09)	**0.001**	999	A(S120), B(S132), C(S134),D(S142)	yes	?	?	bold, approach host
*Neoasterolepisma gauthieri*	Lepismatinae	*Messor*	3	26	6	0.53 ± 0.07 (0.87 ± 0.09)	0.189	10	A(S133)	yes	?	?	–
*Neoasterolepisma lusitanum*	Lepismatinae	*Messor*	9	29	6	0.77 ± 0.08 (0.87 ± 0.09)	**0.014**	999	A(S138), B(S149)	yes	?	?	bold, approach host
*Neoasterolepisma soerenseni*	Lepismatinae	*Messor*								?	?	?	bold, approach host
*Neoasterolepisma spectabile*	Lepismatinae	*Messor*	41	29	12	0.62 ± 0.08 (0.87 ± 0.09)	**0.001**	999	A(S104), B(S119), C(S128), D(S129), E(S133), F(S135), G(S136)	yes	?	no	bold, approach host
*Tricholepisma aureum*	Lepismatinae	*Messor*	31	24	13	0.68 ± 0.10 (0.87 ± 0.09)	**0.001**	999	A(S201), B(S202), C(S203), D(S204)	yes	likely	?	–
*Neoasterolepisma wasmanni*	Lepismatinae	*Camponotus*	5	26	9	0.53 ± 0.03 (0.87 ± 0.09)	**0.016**	126	A(S205)	yes	?	?	–
*CAMPONOTUS* SPECIALIST													
*Tricholepisma indalicum*	Lepismatinae	*Camponotus*	5	21	1	0.79 ± 0.03 (0.87 ± 0.05)	**0.016**	126	A(S131)	yes	?	?	–
*Aphaenogaster* specialist													
*Neoasterolepisma delator*	Lepismatinae	*Aphaenogaster*	12	27	6	0.62 ± 0.10 (0.86 ± 0.06)	**0.001**	999	A (S102), B (S111), C(S117), D(S139)	yes	?	?	bold, approach host
HOST GENERALIST													
*Neoasterolepisma curtiseta*	Lepismatinae	*Aphaenogaster*	1	16	23	0.36 (0.86 ± 0.06)	–	6	(A)S121	yes	no	no	–
	Lepismatinae	*Camponotus*	4	25	8	0.48 ± 0.03 (0.87 ± 0.05)	0.056	35	(B)S124, C(S118)	yes	no	no	mild avoidance + host attraction
*Atelura formicaria*	Atelurinae	*Lasius*	9	11	4	0.33 ± 0.02 (0.90 ± 0.06)	**0.001**	999	A(S303), B(S302)	no	no	yes	mild avoidance + host attraction
*Proatelurina pseudolepisma*	Atelurinae	*Lasius*	4	17	4	0.33 ± 0.01 (0.90 ± 0.06)	**0.042**	70	A(S206)	yes	?	?	mild avoidance + host attraction
	Atelurinae	*Tetramorium*	2	19	2	0.72 ± 0.14 (0.82 ± 0.05)	0.571	20	B(S108), CS110)	yes	?	?	–
FACULTATIVE ASSOCIATE													
*Lepisma baetica*	Lepismatinae	*Tetramorium*	7	17	1	0.57 ± 0.06 (0.82 ± 0.05)	0.**025**	120	A(S109), B(S113), C(S116), D(S143)	yes	no	?	mild avoidance
UNASSOCIATED/FACULTATIVE													
*Lepisma saccharinum*	Lepismatinae	*Formica*	4	32	26	0.25 ± 0.04 (0.83 ± 0.03)	**0.016**	126	A(S301)	no	no	no	strong avoidance

N: number of silverfish samples for CHC determination; shared CHCs (N): number of CHC peaks shared with the host; idiosyncratic CHCs: peaks not shared with the host; BC (host BC): average Bray-Curtis similarity between silverfish and host colony workers; Bray-Curtis similarity between nestmate workers summarized for the host genus was put in brackets; P: *P*-value of the PERMANOVA tests; Perm.: number of unique permutations: if number > 999, 999 permutations were run. Letter codes used in the NMDS plots in Additional file 20 correspond with the colony identity indicated in brackets. Active production of CHCs based on experiments with moulted individuals, suppression of CHCs based on Fig. 7, behaviour based on Fig. 1

### Chemical analysis: CHC profiles of moulted individuals

Fourth, we assessed differences among the profile of freshly moulted and individuals sampled in association with the host colony in the *Messor* specialists *T. aureum* and *N. crassipes*. To obtain moulted individuals, we put some individuals in isolation from ants in a small container for some days, after moulting (after 3–6 days in isolation) they were not re-united with the host ants, but directly frozen in individual vials awaiting CHC analysis. The profiles of freshly moulted and associated individuals were statistically compared using a separate PERMANOVA for both species (adonis function, BC-matrix based on the compositional CHC composition). We also compared the CHC profiles of moulted individuals of the generalist *N. curtiseta* with two different host species, *Camponotus* sp. and *Formica* sp.

### Chemical analysis: silverfish CHC concentration

Finally, we compared the CHC concentrations between the silverfish species using a Kruskal Wallis test to assess whether some silverfish suppress the CHCs. The CHC concentration per individual was approximated by dividing the total amount of CHCs by the dry body weight. As all silverfish species have a similar body shape, we believe that we can use here body mass as a proxy for cuticular surface area. The amount of CHCs per sample (μg) was calculated by summing up all masses of the CHC peaks (for their calculation, see above) present in the sample. Dry body weight was determined with a balance (Brand: OHAUS; accuracy: 0.1 mg) after drying the individuals in an oven at 60 °C for 48 h. We did not compare the silverfish CHC concentrations with those of the host ants. The body shapes of ants and silverfish are completely different, which makes comparisons in CHC concentrations, based on dry weight as proxy for surface area, inaccurate (see discussion Additional file 3 in [43]). 

## Results

### Behavioural assay: ant-silverfish interaction

Silverfish species elicited different degrees of aggression (Kruskal-Wallis rank sum test, Chi^2^ = 15.0, *df* = 3, BH adjusted *P* = 0.024, Fig. 1). The highest levels of ant aggression were recorded towards unassociated and facultative silverfish (see biting, opening mandibles, proportion aggression, effective bites, Fig. 1). Host specialists and generalists elicited moderate levels of aggression, but they were rarely effectively bitten.

The behavioural repertoire of the silverfish species recorded in the standardized behavioural assays varied considerably (PERMANOVA: Pseudo-*F* = 4.99, *P* = 0.001, Fig. 1). *Messor* specialists were the boldest species and regularly approached their host from the front. In contrast to less host specific silverfish, they did not usually display avoidance, i.e., they allowed frontal contact with the ants. In some cases, they also accepted antennation by ants. Unassociated silverfish were also antennated, but this happened when they were injured by the ants. Host following (> 2 s) was typical of generalist and host specialist species and could last for more than 30 s. Pass under and stay under (> 2 s) were likely used to contact the ants when host workers were large, while avoiding ant aggression at the same time. Passing over behaviour is typical of unassociated silverfish, used for escaping quickly from ant aggressions. The generalist *Proatelurina pseudolepisma* and specialist species also used this tactic, but to a smaller extent. These species may resort to passing over (running or walking over the ant) to avoid frontal contact of long duration and acquire nest odour. Host specialist and generalist myrmecophilous silverfish regularly approached their host from the back and might stay behind the ant for a while, but this was rarely observed in unassociated silverfish (Fig. 1).

Nevertheless, these results have to be interpreted cautiously because the behaviour of the silverfish is dependent on the degree of aggression of the host ant. We can compare different silverfish species with the same host species/genus (e.g., C*amponotus* and *Messor* are targeted as hosts by all types of silverfish, Fig. 1). Additionally, the heatmap analysis plot (Fig. 1) also showed for generalist and facultative silverfish that interactions towards a particular species are similar between different host ants (e.g., *N. curtiseta* rows have almost similar colours).

### Behavioural assay: silverfish survival

Silverfish survival was highly different along the host specificity gradient. Table 1 indicates that silverfish specialized in *Messor* ants had a higher survival rate when exposed to their host after capture (96% overall, *N* = 150) than *Aphaenogaster* specialists (63.6%, *N* = 33), generalists (59.7%, *N* = 62) and facultative associates (80%, *N* = 10). Unassociated species did not survive (0%, *N =* 7) in these tests (Table 1), although most of these silverfish survived in standard tests when the surface of the arena was larger. In generalist species, survival was usually lower with bigger or more aggressive ants; for example, the survival of generalist silverfish with *Camponotus* + *Cataglyphis* + *Iberoformica* + *Messor* was 52% (*N* = 20) while their survival with *Tapinoma* + *Pheidole* + *Tetramorium* was 73% (*N* = 10). So differences observed among experiments with different ant taxa were probably dependent on the type of the ant. But it should be noted that, if we compared the results of tests with the same host ant over the gradient of specialization of silverfish, survival increased with a higher degree of host specificity. Thus, for example, survival of silverfish tested with *Messor* as host were 96% for specialists, 55% for generalists + facultative (*N* = 9) and 0% (*N* = 5) for unassociated silverfish.

### Chemical analysis: comparison of CHC profiles

**Fig. 2:**
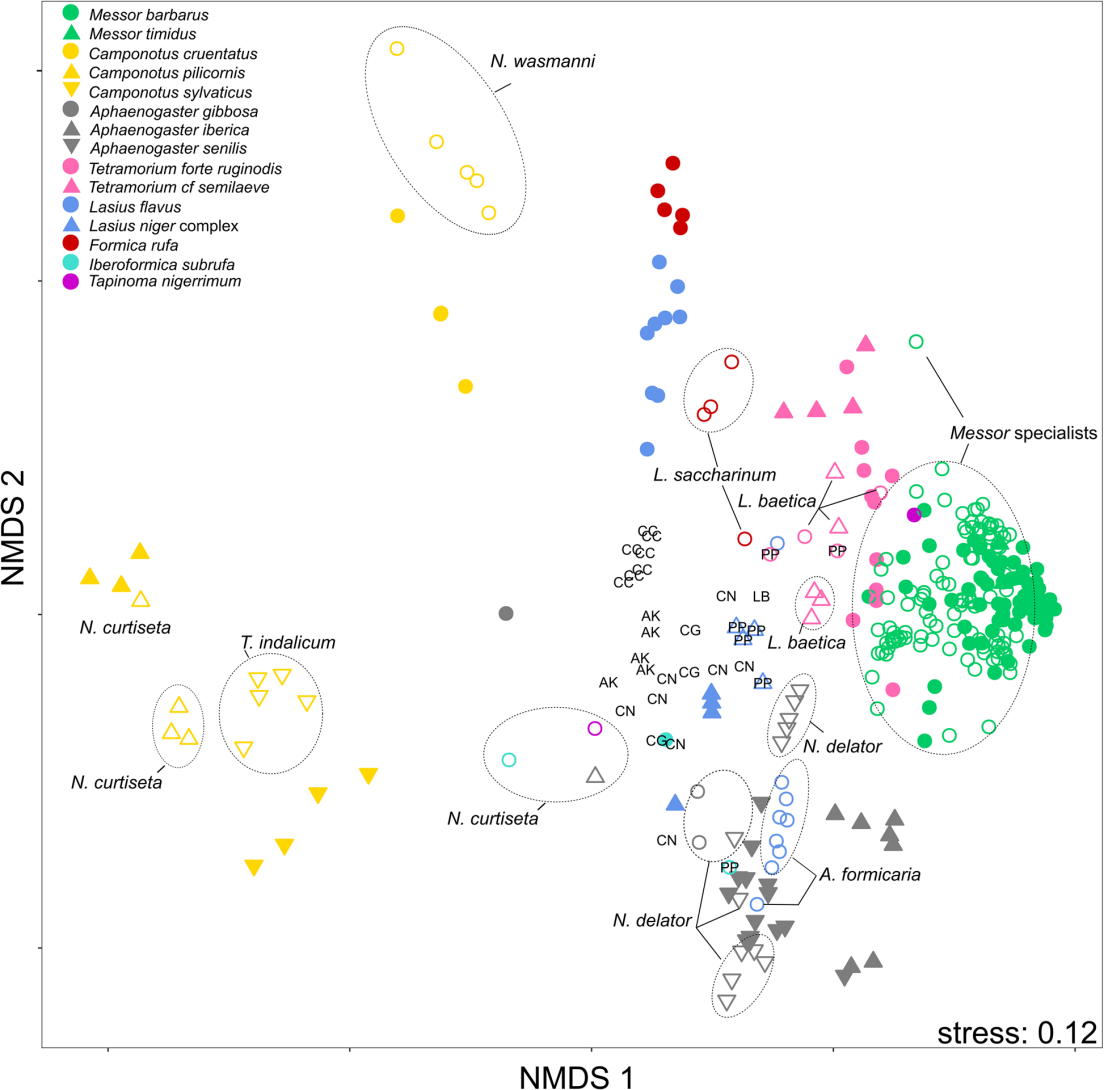
CHC similarity among silverfish and host ants. NMDS plot displays the Bray-Curtis similarities for all detected CHCs (*N =* 199). Filled symbols represent the host ants (congeneric species have the same colour), open symbols represent silverfish associated with ants. For clarity, the seven *Messor* specialist species are not specified on this plot but grouped as *Messor* specialists (green open symbols). A detailed NMDS plot with each *Messor* specialist species specified is given in Additional file 21. The colour and shape of the silverfish symbols correspond with the colour and shape of their host ant. Species identity of myrmecophilous silverfish is given on the plot, except for *Proatelurina pseudolepisma* which is indicated with a letter code (PP) for clarity. Unassociated silverfish are represented with a letter code: *Allacrotelsa kraepelini* (AK), *Ctenolepisma ciliatum* (CC), *Ctenolepisma nicoletii* (CN), *Lepisma baetica* (LB – one individual not associated with ants), *Ctenolepisma guadianicum* (CG)

**Fig. 3:**
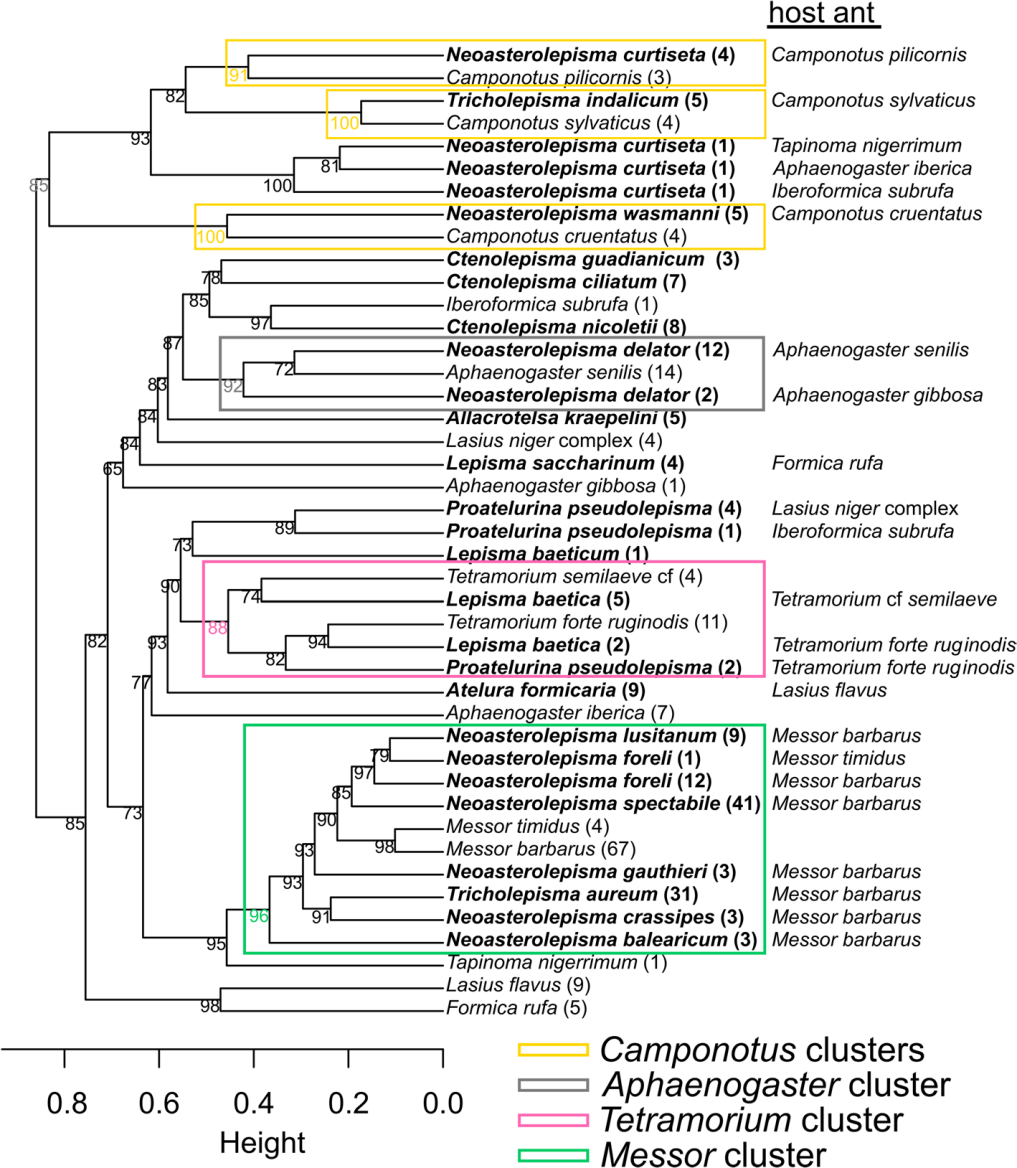
A hierarchical cluster analysis (average linkage method) on the matrix with the Bray Curtis similarity distances between the CHC profiles of the ants and silverfish. To avoid overloading of the tree, we grouped the samples of the silverfish found with the same host species and the ant samples per species (by averaging the BC similarites in the matrix), number of samples in each group/branch in brackets. To assess the statistical support of the clusters, we applied multiscale bootstrapping (1000 bootstraps) with the modified pvclust package for the Bray-Curtis similarity matrix. The approximately unbiased *P*-values are given for each cluster. Values greater than 95% are considered significant

We distinguished 199 different CHC peaks across the silverfish and host ant samples. An overview of the identified peaks and the proportional composition per sample can be found in Additional file 19. Characteristic chromatograms of the silverfish species and their host ants are displayed in Additional file 20. We found a large variation in the cuticular hydrocarbon profiles between the tested species (Fig. 2, Additional file 20). As predicted, most myrmecophilous silverfish species grouped with their respective host species/genera (cf. *Camponotus* clusters, *Messor* cluster, *Aphaenogaster* cluster, *Tetramorium* cluster) and shared many of their peaks with their host (Table 3). Similarity at the host species level is clear in this multivariate plot with the three *Camponotus* species separately clustering with their associated silverfish. The observed host-symbiont grouping in the NMDS plot was supported by the high bootstrap values (approximately unbiased *p*-values, [51]) in the parallelly conducted hierarchical cluster analysis (Fig. 3). Values greater than 95% are considered significant [51].

**Fig. 4:**
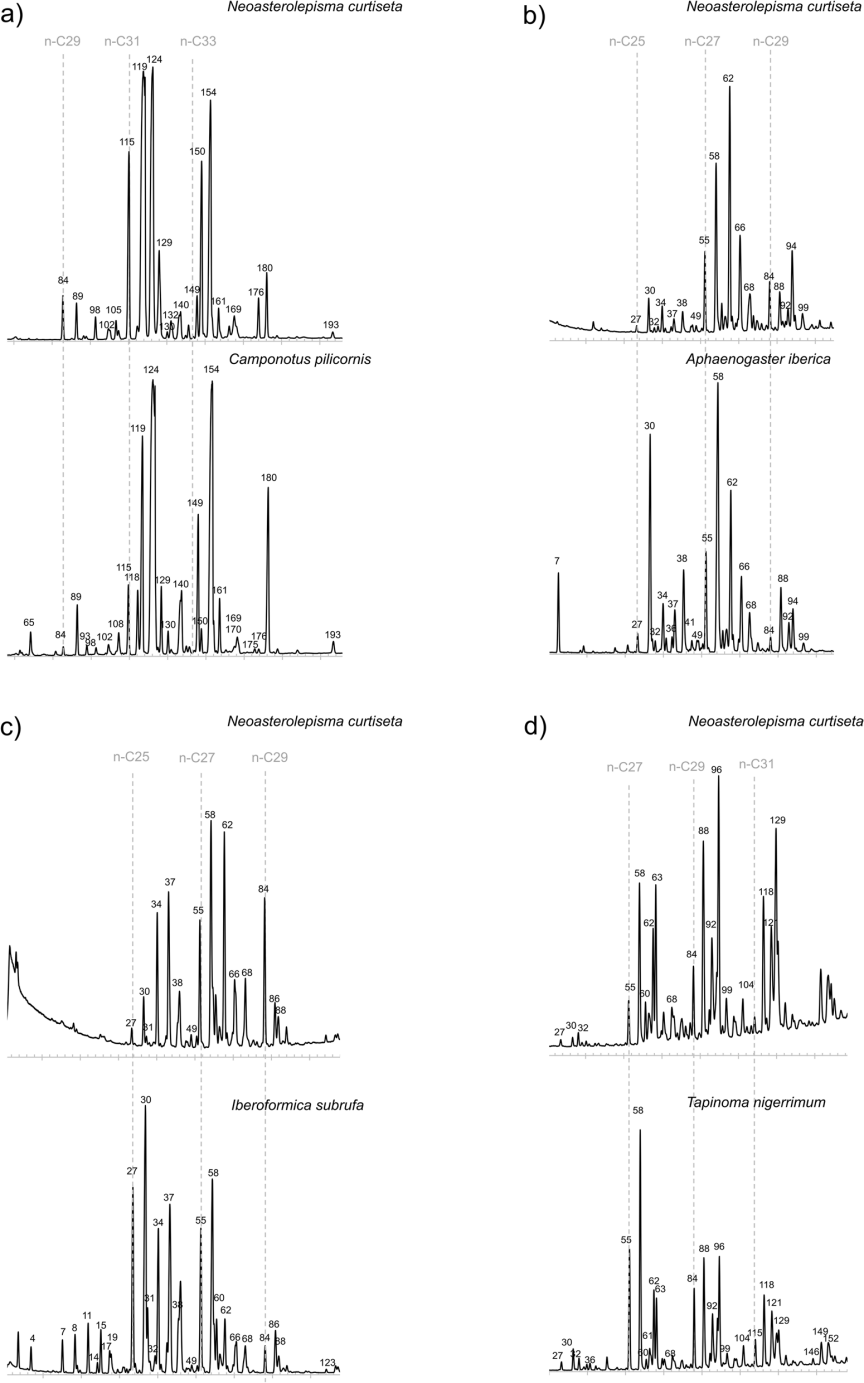
Representative plastic hydrocarbon profiles of the host generalist *Neoasterolepisma curtiseta* associated with different hosts: **a**) *Camponotus pilicornis*, **b**) *Aphaenogaster iberica*, **c**) *Iberoformica subrufa* and **d**) *Tapinoma nigerrimum*. Peak identities of the CHCs can be found in Additional file 19

The NMDS analysis focused on the *Messor* ants and the *Messor* specialists further stressed the close chemical resemblance between these species. The plot displayed some grouping of the silverfish individuals at the species level (colour codes in Additional file 21). Within the species groups, there was a tendency of clustering at the host colony level (letter codes in Additional file 21). A pairwise comparison between the chromatograms and NMDS plots of the silverfish species and their host ants stressed the strong overlap in the chemical profile of *Messor* specialists (*N. balearicum*, *N. crassipes*, *N. foreli, N. gauthieri calva*, *N. lusitanum*, N*. spectabile*, *T. aureum*) *Aphaenogaster* specialists (*N*. *delator*), *Camponotus* specialists (*Tricholepisma indalicum*), generalists (*N. curtiseta, P. pseudolepisma*) and the facultative *L. baetica* with their respective host ants (Fig. 2, Additional file 20). *Neoasterolepisma wasmanni* is considered as a *Messor* specialist [40], but it was found with *Camponotus*. The profile was also very similar to its alternative *Camponotus* host (Fig. 2, Additional file 20). The generalist *N. curtiseta* and the facultative *L. baetica* had variable profiles that match with the host species specific CHC profiles (Fig. 4). Although there was in most silverfish species a close host-silverfish resemblance in the hydrocarbon profile, most of them could be discriminated from their host ant (NMDS plots in Additional file 20, PERMANOVA results in Table 3). Lastly, the profile of the unassociated silverfish *L. saccharinum* and its host *F. rufa* was distinct (Additional file 20).

### Chemical analysis: CHC profiles of moulted individuals

**Fig. 5:**
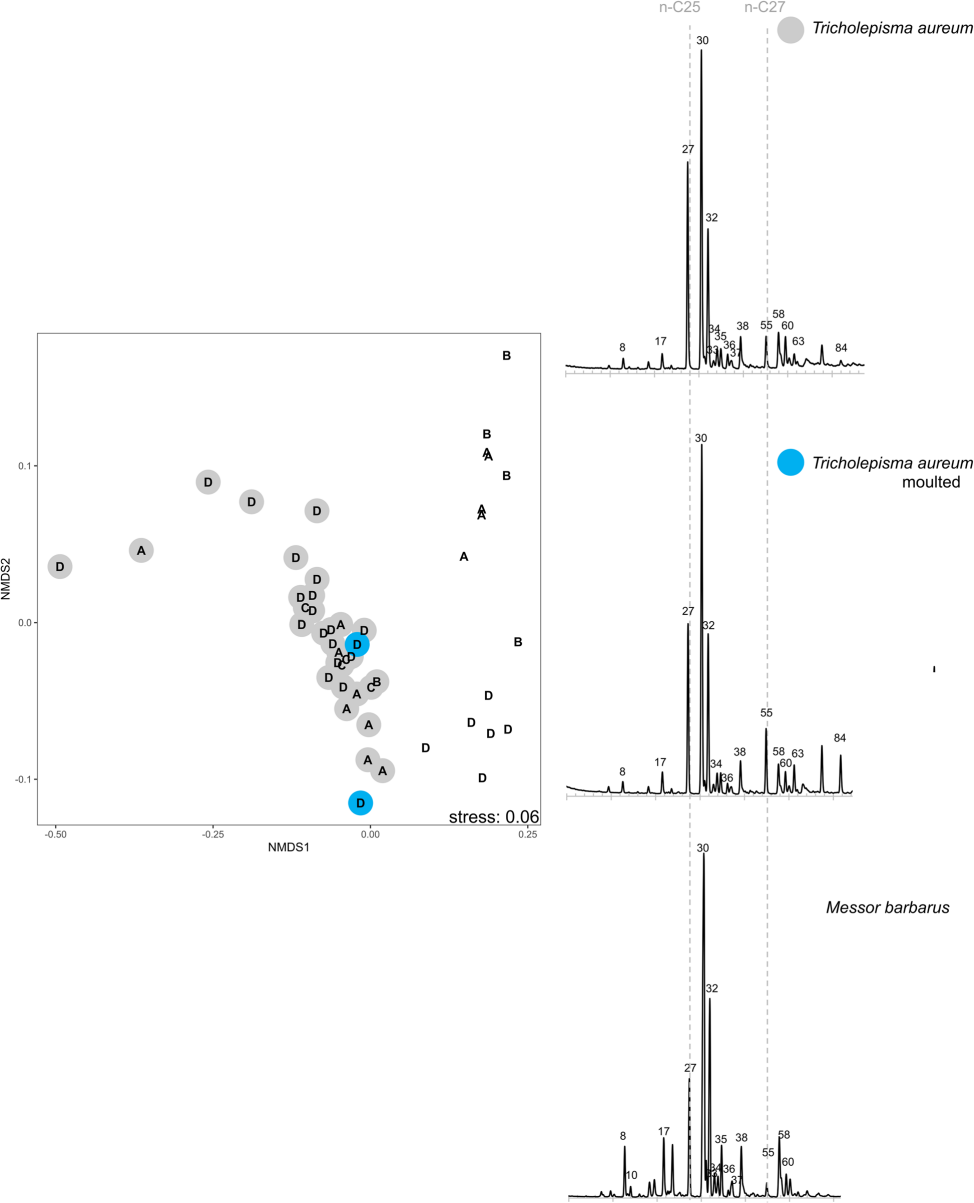
Representative cuticular hydrocarbon chromatogram of the *Messor* specialist *Tricholepisma aureum, a* moulted *T. aureum* individual*,* and the host *Messor barbarus.* Peak identities of the CHCs can be found in Additional file 19. The dissimilarities in the CHC profiles are displayed with a NMDS plot. Silverfish are represented by coloured circles (grey: associated with the host, blue: isolated individual and moulted) around a letter code. Ant individuals are depicted by a letter code without coloured circle. The letter code refers to the host colony (see Table 3)

**Fig. 6:**
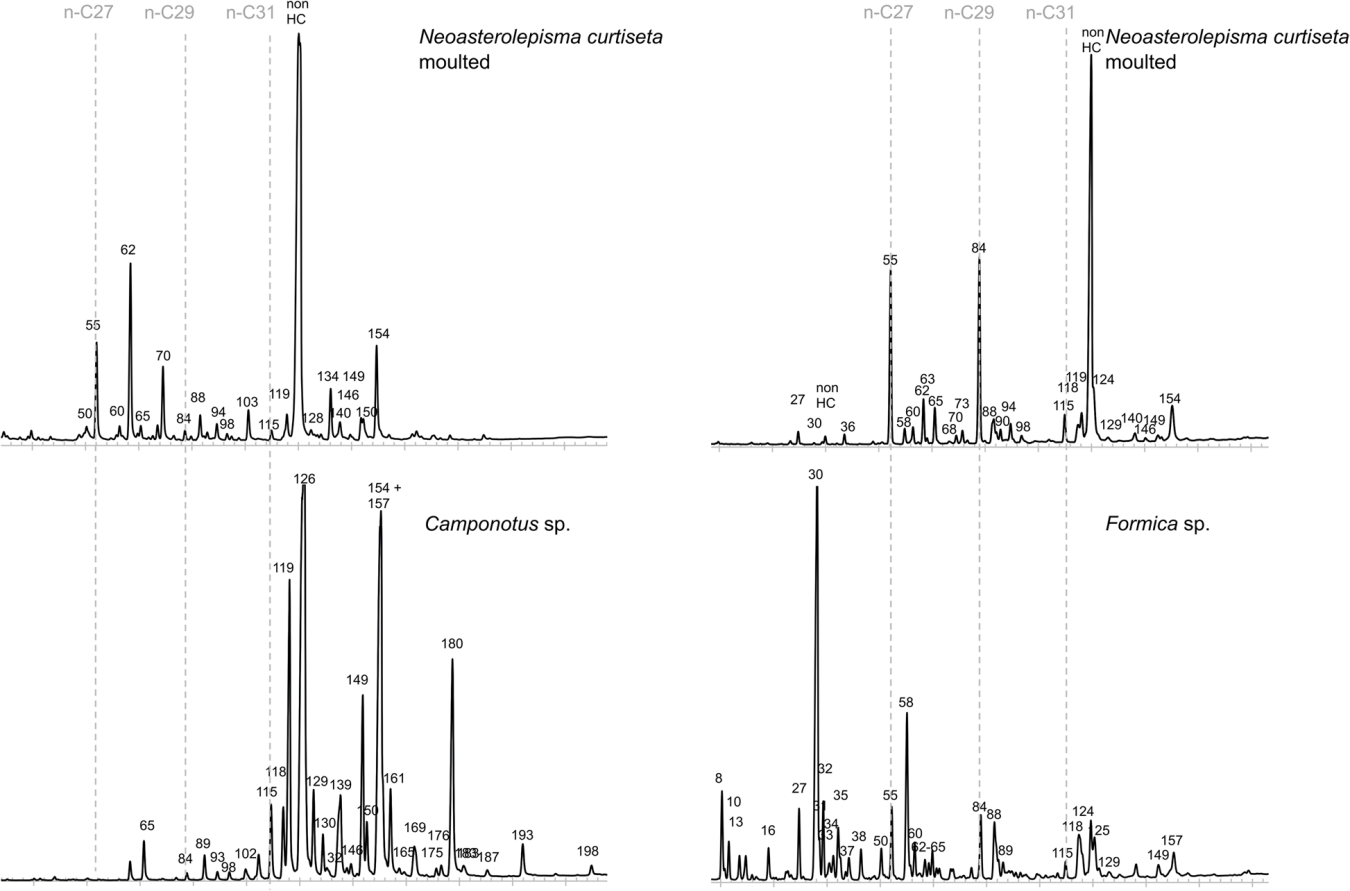
Moulted individuals of the generalist *N. curtiseta* have different CHCs than their host (compare with the matching profiles of ant-associated *N. curtiseta* in Fig. 4). Peak identities can be found in Additional file 19. The peak labelled as non-HC is not a hydrocarbon, but likely a steroid

The profiles of the *Messor* specialists *T. aureum* (Fig. 5, PERMANOVA based on associated and moulted individuals of colony S204: Pseudo-*F* = 0.5, *P* = 0.126, 190 permutations, 18 associated silverfish: BC similarity to workers host colony S204 = 0.68 ± 0.11 vs 2 moulted silverfish from colony S204: BC similarity to workers host colony S204 = 0.77 ± 0.01) and *N. crassipes* (Additional file 20, BC similarity of 3 associated silverfish to workers host colony S201 = 0.71 ± 0.01 vs BC similarity of 2 moulted silverfish to workers host colony S201 = 0.66 ± 0.08) did not change after moulting in absence of ants. Moulted individuals of the generalist *N. curtiseta* associated with *Camponotus* sp. and *Formica* sp. carried completely different profiles than their hosts, (Fig. 6, host *Camponotus*: 2 moulted silverfish, BC similarity to host colony = 0.10 ± 0.05) or *Formica* (host *Formica*, 2 moulted silverfish, BC similarity to host colony = 0.17 ± 0.13). By contrast, associated *N. curtiseta* individuals could reach a medium degree of similarity (BC similarity to *Camponotus* host: 0.48 ± 0.03, Additional file 20). The profiles of the moulted *N. curtiseta* were very similar and were not affected by their original *Formica* or *Camponotus* association (too few permutations available to test significant differences between host and *N. curtiseta*) (Fig. 6).

### Chemical analysis: silverfish CHC concentrations

**Fig. 7:**
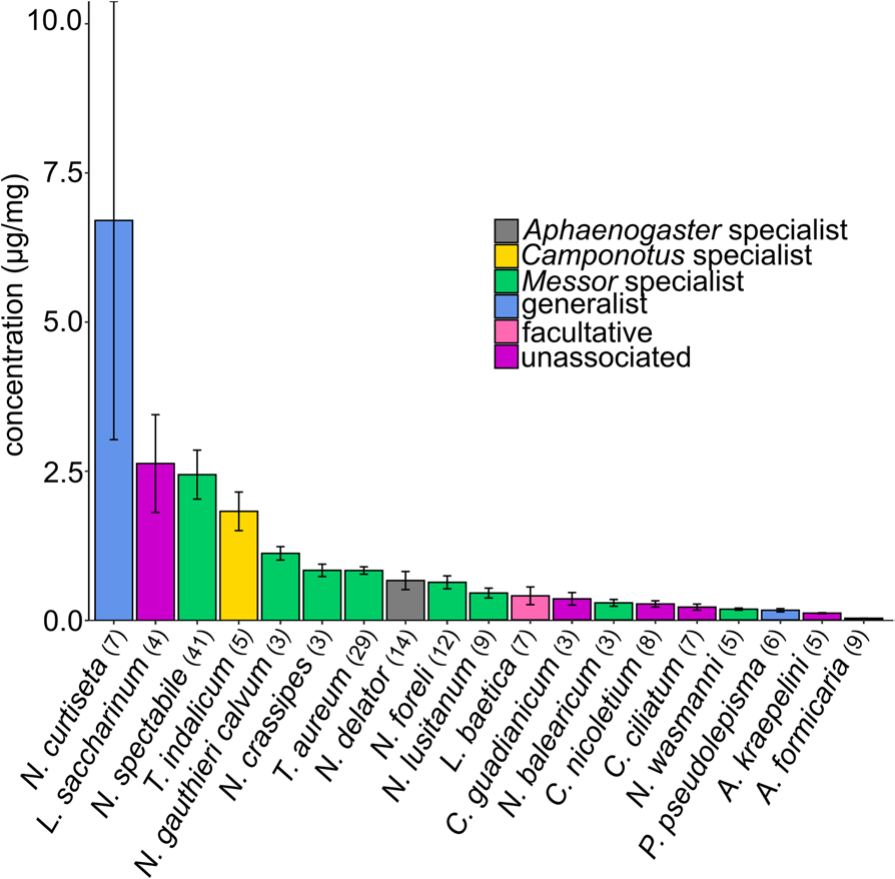
Bar plot comparing the CHC concentrations (±SE), i.e. total CHC mass (μg) / dry body weight (mg), for all analysed silverfish. The degree of host specificity of the silverfish is indicated with a colour code

CHC concentrations (amount of CHCs divided by dry body weight, μg/mg) of the tested silverfish species differed considerably (Kruskal-Wallis test, χ^2^ = 105.5, *df* = 18, *P* < 0.001). The lowest CHC concentration was detected in the generalist *A. formicaria* (0.03 μg/mg ± SD 0.01) which is more than 200-fold lower than the highest detected CHC concentration, found in the generalist *N. curtiseta* (6.70 μg/mg ± SD 9.72, Fig. 7). CHC concentrations (μg CHC/ mg body mass) of moulted silverfish (*N. crassipes* 1.08 ± 0.48, *N = 2*; *T. aureum* 0.79 ± 0.14, *N =* 2) were in the same range as these of associated silverfish (*N. crassipes*: 0.92 ± 0.12, *N =* 3; *T. aureum:* 0.84 ± 0.07, *N =* 31).

## Discussion

We demonstrated in a large group of 16 ant-associated silverfish species characterized by different degrees of host specificity the use of a variety of behavioural and chemical integration strategies. There was a close link between behavioural specialization and host specificity, with more host-specific species being bolder and more inclined to approach the host. A relationship between chemical specialization and host specificity was less clear as advanced chemical deception strategies were surprisingly present in all species along the host specificity gradient, even in a facultatively associated species. Nevertheless, active production of the nestmate recognition cues of the host ant was only found in the most host specific species.

*Messor* specialists displayed a trend of behavioural specialization, elevated boldness, and higher intimacy compared to silverfish with a broader host range. They were more inclined to allow frontal approaches, whereas generalists approached mainly from the back. The behaviour of the host specialists is however relatively rudimentary compared to the behaviour that for example beetle specialists developed to achieve intimate social integration [16, 32, 52–54]. The most specialized beetles are groomed, transported, can freely walk in the nest, and climb on the host workers, but this repertoire is clearly absent in silverfish-host interactions. Exchange of food droplets (trophallaxis) from the hosts to a symbiont is also a relatively specialized behaviour [33] that is present in the generalist species *A. formicaria* ([55], pers. observations TP) and probably occurs in the second species of Atelurinae studied, *P. pseudolepisma*. In the lineage of Lepismatinae which includes host generalists and specialists in different types of ants, trophallaxis has not been observed currently, but further experiments are required to unravel their trophic behaviour. The behaviour of generalist species was also more specialized than that of facultative and unassociated species. Compared to the facultative species, host generalists and specialists often stayed behind a worker or closely followed a worker for some time (Additional file 12: video S10). Probably this behaviour allows the silverfish to keep contact with the host colony, to co-forage and find new nests, and likely help them to acquire hydrocarbons. Although speculative, a first specialization in behaviour in the Lepismatinae may have evolved with the transition to generalist myrmecophily and have been further finetuned within the clade of the *Messor* specialist group (Additional file 1).

Intimate and bold behaviour with the ant host is typically intertwined with chemical deception of the host’s nestmate recognition cues [16, 19, 24–26]. On one hand, chemical mimicking enables the approach and interaction with the host as the associate is not perceived as an intruder. On the other hand, intimate behaviour may also result in a better chemical deception when intimate behaviour (grooming, rubbing, food begging ...) results in the passive transfer of host’s cues. The presence of chemical mimicking of the host colony odour was already suggested almost 100 years ago by Erich Wasmann and evidenced in numerous studies from the 1980’s onwards (see detailed overview Table 1 in [25]). Most studies typically compared one or a few guests with their host. Here we demonstrated the pervasiveness of this strategy in the species-rich group of silverfish associated with European ants [13, 40]. In the silverfish lineage of Lepismatinae, all the tested myrmecophilous species clearly resem bled their host worker chemically. In the two tested silverfish in the subfamily Atelurinae, both generalists, the chemical strategies were different: *Atelura formicaria* clearly adopted the insignificance strategy. *Proatelurina pseudolepisma* acquired imperfectly the nest odour, but chemical insignificance may also play here as the concentrations of CHCs were very low. It was surprising that the facultative associate *Lepisma baetica* (Lepismatinae) also carried identical cues as its host ants as this strategy is expected in specialized and integrated guests, but it can occasionally be present in facultative guests, see for example Death’s-head hawkmoth [56]. It is surprising that this very accurate deception does not go hand in hand with bolder behaviour, as seen in the more host specific species.

Silverfish as primitive ametabolous insects shed and replace their cuticle during adulthood. The hydrocarbons of insects remain on the exuvia, thus they can actively produce and/or passively acquire (from the nest material, ants, exuvia ...) a new profile after a moult [57]. We found that the *Messor* specialist *N. crassipes* and *T. aureum* retained their chemical profile after moulting in absence from host ants, which hint that these species, and probably the other related *Messor* specialists (Additional file 1), can actively produce the ant’s hydrocarbons. There is a possibility that they acquired (a part of) the profile by rubbing or feeding on the exuvia. We would then expect that the CHC concentrations of the moulted individuals would be lower than those of associated silverfish due to incomplete transfer during rubbing or metabolic loss, but CHC concentrations remained in the same range after moulting. The production of a host-specific signal is known as chemical mimicry and has been demonstrated in well integrated social insect guests using radioactive isotopes [58, 59] or by showing that the profile was retained in isolation from the host [28, 60]. The generalist species *Neoasterolepisma curtiseta* and the facultative species *Lepisma baetica* were able to flexibly match their chemical profile to host species with very different profiles similar to other non-host specific myrmecophiles, such as *Myrmecophilus* crickets [61] and different beetles and the silverfish *Trichatelura manni* associated with different army ant species [43]. After moulting, *N. curtiseta* was no longer a mimic of its host and carried an idiosyncratic profile irrespective of its original hosts. Therefore, the plasticity found in this group probably arises from the passive acquisition of the host’s bouquet, a chemical deception strategy known as chemical camouflage [62]. *Lepisma saccharinum* usually lives away from ants, but a relatively stable (at least 3 years old) population was found in some red wood ant nests [44]. *L. saccharinum* retained its idiosyncratic profile, unlike the congeneric facultative *L. baetica*. This suggests that merely living in an ant nest does not automatically result in the acquisition of the host’s profile in silverfish. The transfer of host cues can be accomplished through physical contact with the host or nest material [61, 63], alternatively they may recycle hydrocarbons by feeding on living ants, their brood or scavenging on dead ants [64]. At this point, physical contact with the host and the nest material is the more plausible strategy for generalist silverfish (and perhaps some facultative species) to acquire hydrocarbons. Recordings showed frequent attempts of contact using back and lateral approaches, passing over or under, etc. (Additional file 12: video S10). These behaviours are not as striking as the rubbing behaviour shown by the tropical myrmecophilous silverfish *Malayatelura ponerophila* (additional video in [63]) but probably efficient enough to acquire hydrocarbons. *Messor* specialists that, as we have demonstrated, are likely to produce a *Messor* profile de novo (chemical mimicry) also sought physical contact (see videos S8, S9, S14 and S15). This behaviour probably helps them to finetune their general *Messor* profile and approach the colony-specific blend. This is in line with other studies that demonstrated or suggested that some social insect guests combine active production of the host semiochemicals with passive transfer after adoption in the host colony [58, 65]. The generalist species *Atelura formicaria* was the only ant-associated silverfish with a chemical profile deviating from the host’s bouquet. However, the detected concentration of CHCs was extremely low, which hints that this species relied on chemical insignificance. This nonspecific deception strategy enables host switching and was previously detected in non-integrated guests and myrmecophiles with a very broad host range [25, 27, 28]. Low hydrocarbon concentrations likely make these species more sensitive to drying out, as the primary function of a waxy cuticular hydrocarbon layer in insects is anti-desiccation [57]. As all ant-associated species show some form of chemical deception, there is no clear trend to specialization in chemical strategies with increasing host specialization. Chemical mimicry and camouflage can both be seen as advanced strategies to dupe the host [19, 26]. Chemical mimicry, as probably present in the silverfish host specialists, offers protection to the myrmecophile when it disperses and associates with a new colony, whereas species relying on camouflage must evade initial aggression by agile behaviour, rapid rubbing of the host or possibly the use of volatiles [26]. However, this passive strategy gives more flexibility compared to a permanent chemical cloak as host species with very different chemical profiles may eventually be infested [26, 62]. This is supported by the generalist *N. curtiseta* in our study which likely adopted chemical camouflage to infest several different host species characterized by distinct chemical profiles (Fig. 4). Note that species that actively produce the host chemicals (chemical mimicry) will also need to passively acquire colony-specific compounds for a better integration, as nestmate recognition happens at the colony-level [19]. It is tempting to speculate that chemical mimicry arose in the common ancestor of *Messor* specialists (Additional file 1). However, the evolutionary trajectory of chemical specialization within the clades of myrmecophilous silverfish is unclear and, at this point, we cannot infer how many times chemical camouflage and mimicry evolved independently.

Although the chemical mimicking likely impedes their detection as non-nestmates, all myrmecophiles were eventually detected as intruders and provoked mild to modest degrees of aggression. This is in contrast to many specialized, well-integrated myrmecophiles, which stay undetected and intimately engage in colony life as true nest mates [33]. The chemical resemblance of the tested myrmecophilous silverfish is moderate to strong (max BC similarity of 0.77 in *N. lusitanum*) but not perfect. Well-integrated, specialized myrmecophiles likely show higher BC similarities (cf Table 2 in [43]: BC similarity to the host colony of 0.94 in the army ant beetle *Ecitophya*). However, a recent study on army ant myrmecophi les indicated that myrmecophiles with relatively high chemical similarity to their host were also detected and evoked much higher levels of aggression than species with lower CHC host similarities [43]. The authors hypothesized that other aspects than chemical similarity may be important for achieving social integration and to suppress host aggression, such as the body shape (cfr. Myrmecoid body shape), behaviour, glandular secretions and vibroacoustical signals. In addition, it should be noted that nestmate recognition in social insects is typically based on only a subset of the chemical cuticular profile [48, 49]. This implies that the match with the discriminating subset of host hydrocarbons in the myrmecophilous silverfish may be even higher than here recorded with the full spectra. Imperfect mimicry is quite common in social insect guests and is often associated with some degree of host aggression [25, 39, 66, 67]. The highest level of aggression was predictably observed in the assays with unassociated silverfish. Currently, it is unclear whether the level of provoked aggression is correlated with the potential costs to the colony. Preliminary observations confirmed Janet’s observations [55] that the generalist *A. formicaria* is able to steal food droplets through trophallaxis. But most of the European silverfish probably incur low costs to the host and act more as commensals (cf. diet of a co-habiting beetle in *Messor* ants, [67]). Future studies will try to elucidate the nature of the symbiotic associations in myrmecophilous silverfish by focusing on their trophic preferences. Long exposure to frequent ant interactions was costly for facultative and for the generalist species *N. curtiseta* as they got injured or even killed in small containers. These species tend to live at the periphery of the nest and probably make use of hiding (which was not possible in the container trials) to avoid direct interaction with the ants. Although the host specialist species provoked aggression, they could tolerate long exposure to high ant densities without apparent costs. This discrepancy can be explained by the fact that ants (for example by habituation) or the silverfish behaved differently after interacting over a much longer time frame than this of the behavioural assays. We also have indications that they approached the host more frequently when it was occupied with other tasks, such as brood caring or food manipulation (Additional file 22: video S16). Alternatively, the physiology and morphology of these host specialist silverfish may help to better withstand the stressful conditions.

## Supplementary Information

**Additional file 1.** Phylogeny based on morphological traits of the studied species.

**Additional file 2.** Coordinates of nests where ants and silverfish were collected for chemical analyses.

**Additional file 3: Video S1.** Ant behaviour: ignoring (*N. spectabile* with *Messor*). Silverfish behaviour: frontal approach.

**Additional file 4: Video S2.** Ant behaviour: frontal antennation (inspection) (*Messor* with *N. soerenseni*).

**Additional file 5: Video S3.** Ant behaviour: opening mandibles (*Messor* with *N. spectabile*). Silverfish behaviour: frontal approach resulting in an interaction.

**Additional file 6: Video S4.** Ant behaviour: bite attempt (*Messor* with *N. spectabile*).

**Additional file 7: Video S5.** Ant behaviour: effective bite (*Camponotus* with *Ctenolepisma ciliatum*).

**Additional file 8: Video S6.** Silverfish behaviour: avoidance (*Lepisma baetica* with *Pheidole*).

**Additional file 9: Video S7.** Silverfish behaviour: avoidance (*N. curtiseta* with *Camponotus*).

**Additional file 10: Video S8.** Silverfish behaviour: approach from the back and stay at the back for more than 2s (*N. spectabile* with *Messor*).

**Additional file 11: Video S9.** Silverfish behaviour: approach from the back and lateral rubbing (*N. spectabile* with *Messor*).

**Additional file 12: Video S10.** Silverfish behaviour: host following (*N. spectabile* with *Messor*).

**Additional file 13: Video S11.** Silverfish behaviour: allowing host antennation (*Aphaenogaster* and *N. delator*).

**Additional file 14: Video S12.** Silverfish behaviour: allowing host inspection, but injured by bites (*Ctenolepisma ciliatum* with *Camponotus*).

**Additional file 15: Video S13.** Silverfish behaviour: jumping behaviour (*N. spectabile* with *Messor*).

**Additional file 16: Video S14.** Silverfish behaviour: passing under the host and ignoring (*N. spectabile* with *Messor*).

**Additional file 17: Video S15.** Silverfish behaviour: staying under the host (*N. spectabile* with *Messor*).

**Additional file 18.** Details of survival tests.

**Additional file 19.** Proportional CHC peaks for each sample and id of each CHC peak.

**Additional file 20.** Exemplary chromatograms of silverfish and corresponding host species. NMDS plots based on the Bray-Curtis distances of the shared CHC peaks are also given for ant-silverfish pairs.

**Additional file 21.** NMDS based on the CHC profiles of *Messor* and *Messor* specialists.

**Additional file 22: Video S16.** Silverfish behaviour: approaching the host during larval transport (*N. delator* with *Aphaenogaster*).

## Data Availability

The chemical datasets supporting the conclusions of this article are included within the additional files. Behavioural datasets are available from the corresponding author on reasonable request.
