# Supplementary material for: Chemical and behavioural strategies along the spectrum of host specificity in ant-associated silverfish
Source: BMC Zool. 2022 May 11;7:23. doi: 10.1186/s40850-022-00118-9 (PMC10127367; doi:10.1186/s40850-022-00118-9)
Supplement: Supplementary file 1 — Additional file 1. Phylogeny based on morphological traits of the studied species. [file 40850_2022_118_MOESM1_ESM.docx]

**CURRENT KNOWLEDGE OF THE PHYLOGENY OF ZYGENTOMA**

**No molecular phylogenies are carried out in this order. The molecular markers* of few species are known and only the internal phylogeny of the subfamily Cubacubaninae in the family Nicoletiidae is supported with molecular studies.**


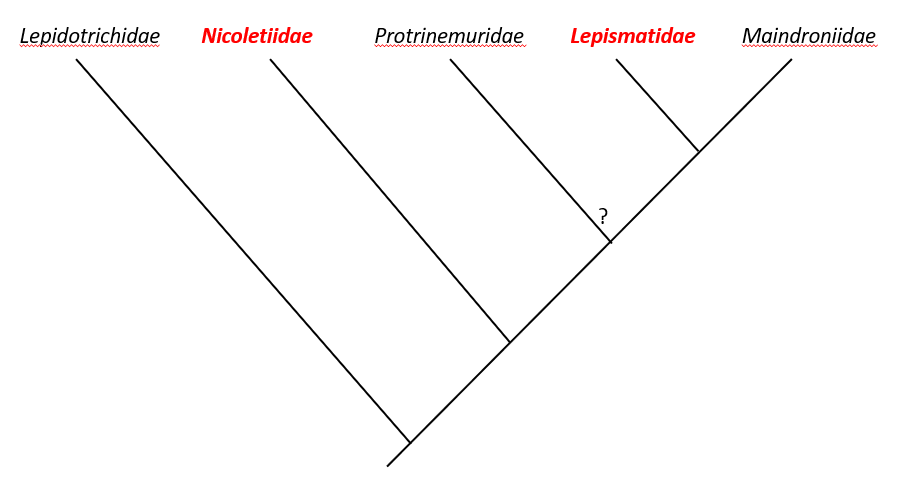


Cladogram of the **order Zygentoma** showing phylogenetic relationships between families of this order, according Koch (2003). It is based on morphological traits. The myrmecophilic species studied in this work belong to the families Nicoletiidae and Lepismatidae, marked with red. The position of the family Protrinemuridae is doubtful. The few data available from molecular data support the position of Lepidotrichidae, Nicoletiidae and Lepismatidae (for example, see Cucini et al., 2021).


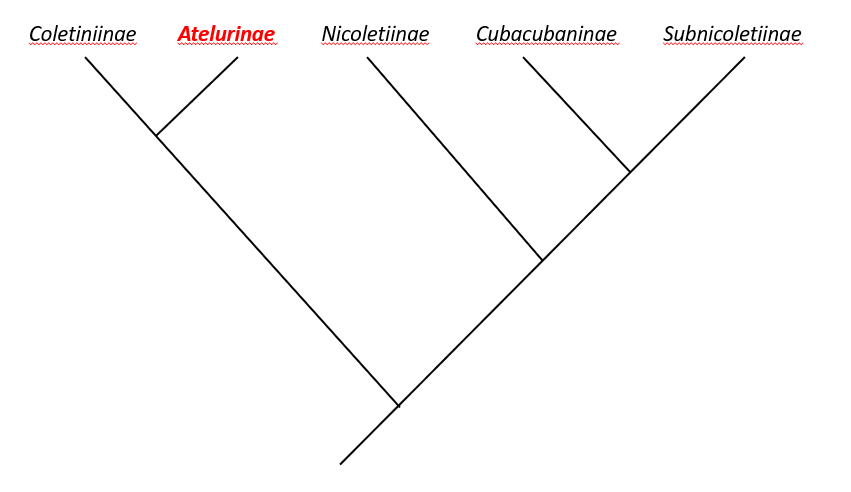


Cladogram showing the phylogenetic relationships between the subfamilies of **the family Nicoletiidae**, based on morphological traits (adapted from Mendes, 2002). Two genera studied in this work belong to the subfamily Atelurinae, whose species are mostly associated to ants or termites. The internal phylogeny of this subfamily has been never analysed.


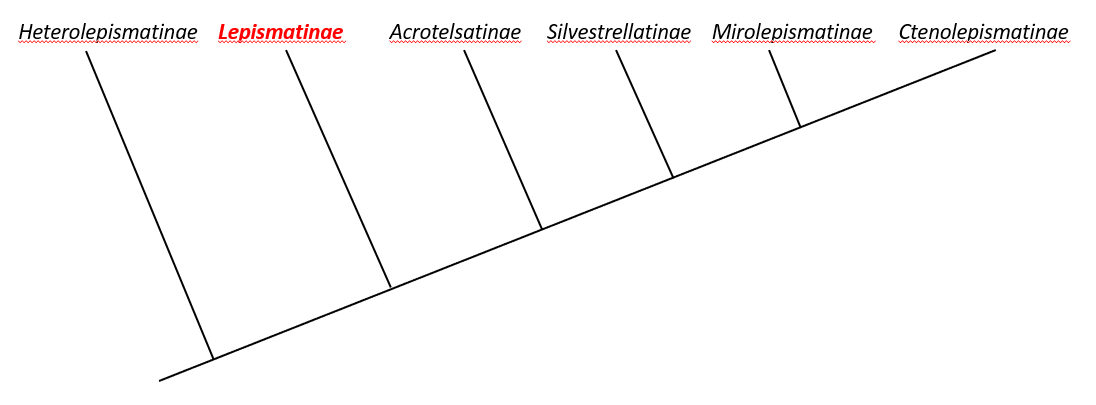


Cladogram showing the phylogenetic relationships between the subfamilies of the **family** **Lepismatidae**, based on morphological traits (adapted from Mendes, 1991). All Lepismatidae studied in this work belong to the subfamily Lepismatinae. These relationships are on discussion since further evidence supports a more plesiomorphic position for Acrotelsatinae. Molecular studies of some subfamilies are being carried out at the moment, but not yet published.


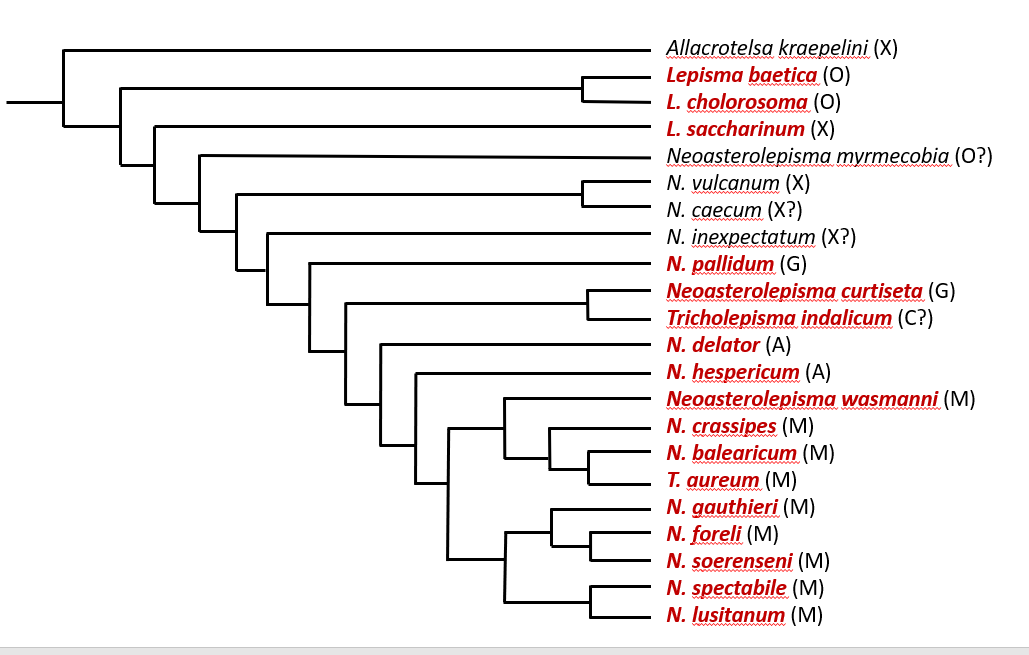


Cladogram based on morphological traits including most species of Southwestern European Lepismatinae. Those included in this study are marked with red. This cladogram was presented by Molero-Baltanás et al. (2017) and here only few modifications are added, mainly to correct the classification of some species. X= xenomyrmecophile (usually non-associated to ants); O = occasional myrmecophile; G = generalist strict myrmecophile; C = *Camponotus* specialist; A= *Aphaenogaster* specialist; M = *Messor* specialist. The ? sign indicates that the number of samples collected of these species is low and their classification has no statistical support.

REFERENCES:

Cucini, C., Carapelli, A., Brunetti, C., Molero-Baltanás, R., Gaju-Ricart, M., Nardi, F. 2021. Characterization of the complete mitochondrial genome of *Neoasterolepisma foreli* (Insecta: Zygentoma: Lepismatidae) and the phylogeny of basal Ectognatha. Mitochondrial DNA Part B. 6. 119-121. 10.1080/23802359.2020.1848480.

Koch, M. 2003. Towards a phylogenetic system of the Zygentoma. *Entomologische Abhandlungen. Staatliches Museum für Tierkunde Dresden* **61**(2): 122–125.

Mendes, L.F. 1991. On the phylogeny of the genera of Lepismatidae (Insecta: Zygentoma). pp. 3–13 *in* Veeresh, G.K., Rajagopal, D. & Viraktamath, C.A. (eds) *Advances in management and conservation of soil fauna.* New Delhi Bombay Calcutta: Oxford & IBH Publishing Co. Pvt. Ltd.

Mendes, L.F. 2002. On the status of the "Protrinemurid" and "Atelurid" Thysanurans (Zygentoma: Insecta). *Boletim da Sociedade Portuguesa de Entomologia* **199**(VII-17): 201–212.

Molero-Baltanás, R., Bach De Roca, C., Tinaut, A., Pérez, J.D., Gaju-Ricart, M. 2017 Symbiotic relationships between silverfish (Zygentoma: Lepismatidae, Nicoletiidae) and ants (Hymenoptera: Formicidae) in the Western Palaearctic. A quantitative analysis of data from Spain. My*rmecological News* **24**:107–22.
