# Supplementary material for: Chemical and behavioural strategies along the spectrum of host specificity in ant-associated silverfish
Source: BMC Zool. 2022 May 11;7:23. doi: 10.1186/s40850-022-00118-9 (PMC10127367; doi:10.1186/s40850-022-00118-9)
Supplement: Supplementary file 2 — Additional file 2. Coordinates of nests where ants and silverfish were collected for chemical analyses. [file 40850_2022_118_MOESM2_ESM.docx]

**Coordinates of the nests used in chemical analysis of ants and myrmecophile silverfish**

| **Code** | **Date** | **Locality** | **Coordinate decimal** | **Country** |
| --- | --- | --- | --- | --- |
| S102 | 07/04/2017 | Córdoba (NE of the city) | 37.9075°N  4.7706°W | Spain |
| S103 | 07/04/2017 | Lucena (Córdoba), Campo Aras | 37.3696°N  4.4716°W | Spain |
| S104 | 12/04/2017 | La Tahá (Granada), Pitres to Capilerilla | 36.9391°N  3.3306°W | Spain |
| S105 | 13/04/2017 | Yegen (Granada) | 36.9794°N  3.1242°W | Spain |
| S107 | 08/05/2017 | Córdoba (NW of the city) | 37.9128°N  4.8197°W | Spain |
| S108 | 03/05/2017 | Córdoba (SE of the city) | 37.8971°N  4.7273°W | Spain |
| S109 | 03/05/2017 | Córdoba (SE of the city) | 37.8971°N  4.7273°W | Spain |
| S110 | 03/05/2017 | Córdoba (SE of the city) | 37.8965°N  4.7293°W | Spain |
| S111 | 03/05/2017 | Córdoba (SE of the city) | 37.8966°N  4.7316°W | Spain |
| S112 | 09/05/2017 | Córdoba (NE of the city) | 37.9090°N  4.7709°W | Spain |
| S113 | 14/05/2017 | Córdoba (SE of the city) | 37.8966°N  4.7316°W | Spain |
| S114 | 09/05/2017 | Córdoba (NE of the city) | 37.9075°N  4.7707°W | Spain |
| S115 | 14/05/2017 | Córdoba (SE of the city) | 37.8971°N  4.7273°W | Spain |
| S116 | 16/05/2017 | Córdoba (NE of the city) | 37.9076°N  4.7708°W | Spain |
| S117 | 16/05/2017 | Córdoba (NE of the city) | 37.9076°N  4.7708°W | Spain |
| S118 | 20/05/2017 | Lucena (Córdoba), Sierrezuela | 37.4274°N  4.5096°W | Spain |
| S119 | 27/05/2017 | Chirivel (Almería) | 37.6027°N  2.2248°W | Spain |
| S120 | 27/05/2017 | Chirivel (Almería) | 37.6027°N  2.2248°W | Spain |
| S121 | 27/05/2017 | Chirivel (Almería) | 37.6027°N  2.2249°W | Spain |
| S122 | 26/05/2017 | Archena (Murcia) | 38.1223°N  1.3082°W | Spain |
| S123 | 01/11/2017 | Cerro Muriano (Córdoba) | 38.0027°N  4.7591°W | Spain |
| S124 | 01/11/2017 | Cerro Muriano (Córdoba) | 38.0026°N  4.7594°W | Spain |
| S125 | 22/11/2017 | Córdoba, Las Quemadas (SE of the city) | 37.8971°N  4.7273°W | Spain |
| S126 | 22/11/2017 | Córdoba, Las Quemadas (SE of the city) | 37.8973°N  4.7273°W | Spain |
| S127 | 22/11/2017 | Córdoba, Las Quemadas (SE of the city) | 37.8973°N  4.7273°W | Spain |
| S128 | 06/12/2017 | Córdoba, Santa Ana de la Albaida (NW of the city) | 37.9072°N  4.8222°W | Spain |
| S129 | 06/12/2017 | Córdoba, Santa Ana de la Albaida (NW of the city) | 37.9072°N  4.8223°W | Spain |
| S130 | 25/05/2018 | Jubrique (Málaga), road to Estepona | 36.5618°N  5.1978°W | Spain |
| S131 | 29/03/2018 | Mazarrón, road to Águilas (Murcia) | 37.5292°N  1.5205°W | Spain |
| S132 | 27/03/2018 | Monte Carmolí (Murcia) | 37.6897°N  0.8443°W | Spain |
| S133 | 27/03/2018 | Monte Carmolí (Murcia) | 37.6897°N  0.8438°W | Spain |
| S134 | 27/03/2018 | Monte Carmolí (Murcia) | 37.6897°N  0.8442°W | Spain |
| S135 | 14/04/2018 | Llíber (Alicante) | 38.7388°N  0.0202°E | Spain |
| S136 | 20/04/2018 | Las Virtudes (Ciudad Real) | 38.6039°N  3.4667°W | Spain |
| S137 | 14/04/2018 | Llíber (Alicante) | 38.7388°N  0.0202°E | Spain |
| S138 | 08/04/2018 | Córdoba W suburb | 37.8710°N  4.8445°W | Spain |
| S139 | 20/04/2018 | Las Virtudes (Ciudad Real) | 38.6038°N  3.4667°W | Spain |
| S140 | 27/03/2018 | Monte Carmolí (Murcia) | 37.6896°N  0.8442°W | Spain |
| S141 | 15/04/2018 | Lucena, Sierrezuela | 37.4270°N  4.5097°W | Spain |
| S142 | 29/04/2018 | Vélez de Benaudalla | 36.8264°N  3.5180°W | Spain |
| S143 | 12/05/2018 | Córdoba, Mirabueno | 37.9064°N  4.7714°W | Spain |
| S144 | 27/05/2018 | Ronda, road to San Pedro de Alcántara (Málaga) | 36.6048°N  5.0709°W | Spain |
| S145 | 24/05/2018 | Rubí (Barcelona) | 41.5094°N  2.0249°E | Spain |
| S146 | 24/05/2018 | Rubí (Barcelona) | 41.5094°N  2.0249 °E | Spain |
| S147 | 18/04/2018 | Córdoba | 37.9789°N  4.7687°W | Spain |
| S148 | 27/05/2018 | Ronda, road to San Pedro de Alcántara (Málaga) | 36.6047°N  5.0709W | Spain |
| S149 | 25/05/2018 | Jubrique (Málaga), road to Estepona | 36.5618°N  5.1976°W | Spain |
|  |  |  |  |  |
| S201 | 21/05/2019 | Laroque-des-Albères | 42.5308°N 2.9383°W | France |
| S202 | 21/05/2019 | Laroque-des-Albères | 42.5328°N  2.9391°W | France |
| S203 | 21/05/2019 | Laroque-des-Albères | 42.5307°N  2.9370°W | France |
| S204 | 21/05/2019 | Laroque-des-Albères | 42.5302 °N 2.9356°W | France |
| S205 | 4/6/2018 | Montpelier | 43.6525°N  3.8962°W | France |
| S206 | 4/6/2018 | Montpelier | 43.6507°N  3.8971°W | France |
|  |  |  |  |  |
| S301 | 9/8/2018 | Poperinge | 50.8853°N  2.7090°E | Belgium |
| S302 | 2/5/2017 | Middelkerke | 51.1760°N  2.7936°E | Belgium |
| S303 | 10/7/2017 | Dinant | 50.2704°N 4.9138°E | Belgium |
|  |  |  |  |  |
