# Supplementary material for: Chemical and behavioural strategies along the spectrum of host specificity in ant-associated silverfish
Source: BMC Zool. 2022 May 11;7:23. doi: 10.1186/s40850-022-00118-9 (PMC10127367; doi:10.1186/s40850-022-00118-9)
Supplement: Supplementary file 18 — Additional file 18. Details of survival tests. [file 40850_2022_118_MOESM18_ESM.pdf]

Details of survival tests silverfish

Messor specialist tests (N=40)

With *Neoasterolepisma foreli*

Number of experiment (N=12)	SURVIVAL (15’)	Number of ants	Number of Silv	Number of Silv died	Ant species
S103	100%	3	1	0	*M. barbarus*
S120	100%	4	3	0	*M. barbarus*
S132	100%	4	4	0	*M. barbarus*
S133a	100%	8	6	0	*M. barbarus*
S135a	100%	8	6	0	*M. barbarus*
S136a	100%	2	1	0	*M. barbarus*
S142	100%	10	3	0	*M. barbarus*
S145a	100%	7	10	0	*M. barbarus*
S035	100%	3	2	0	*M. barbarus*
S023	100%	2	2	0	*M. barbarus*
S024	100%	3	1	0	*M. barbarus*
S134	100%	6	2	0	*M. timidus*

With *N. lusitanum*

Number of experiment (N=8)	SURVIVAL (15’)	Number of ants	Number of Silv	Number of Silv died	Ant species
S114b	100%	2	1	0	*M. barbarus*
S129b	100%	2	2	0	*M. barbarus*
S138a	100%	10	6	0	*M. barbarus*
S149	100%	5	7	0	*M. barbarus*
S038	100%	3	1	0	*M. barbarus*
S051	100%	4	3	0	*M. barbarus*
S056	100%	1	1	0	*M. barbarus*
S252	100%	2	2	0	*M. barbarus*

With *N. spectabile*

Number of experiment (N=20)	SURVIVAL (15’)	Number of ants	Number of Silv	Number of Silv died	Ant species
S104	33,33%	7	6	2	*M. barbarus*
S114a	100%	2	2	0	*M. barbarus*
S119	100%	1	1	0	*M. barbarus*
S123	50%	1	4	2	*M. barbarus*
S128	100%	7	5	0	*M. barbarus*
S129a	100%	4	10	0	*M. barbarus*
S133c	100%	2	2	0	*M. barbarus*
S135b	100%	4	8	0	*M. barbarus*
S136b	100%	3	8	0	*M. barbarus*

S002+ S003	100%	6	6	0	*M. barbarus*
S004 + S005	100%	3	3	0	*M. barbarus*
S016 + S017	100%	2	2	0	*M. barbarus*
S026	100%	1	1	0	*M. barbarus*
S037	100%	3	1	0	*M. barbarus*
S041	100%	12	5	0	*M. barbarus*
S043	100%	5	4	0	*M. barbarus*
S054	100%	9	9	0	*M. barbarus*
S059	75%	4	4	1	*M. barbarus*
S060	75%	4	4	1	*M. barbarus*
S138b	100%	10	1	0	*M. barbarus*

Aphaenogaster **specialist tests (N= 13)**

With *N. delator*

**Number of experiment (N = 12)**	**SURVIVAL (15')**	**Number of ants**	**Number of Silv**	**Number of Silv died**	**Ant species**
S107	0%	2	2	2	*A. gibbosa*
S102	100%	2	2	0	*A. senilis*
S111	100%	5	2	0	*A. senilis*
S117	100%	5	3	0	*A. senilis*
S125	50%	4	2	1	*A. senilis*
S127	50%	9	6	3	*A. senilis*
S139	100%	5	3	0	*A. senilis*
S141	100%	4	1	0	*A. senilis*
S018 + S019	0%	8	3	3	*A. senilis*
S032	60%	5	5	2	*A. senilis*
S402	50%	6	2	1	*A. senilis*
S403	100%	5	1	0	*A. senilis*

With *N. hespericum*

**Number of experiment (N=1)**	**SURVIVAL (15')**	**Number of ants**	**Number of Silv**	**Number of Silv. died**	**Ant species**
S022	100%	2	1	0	*A. senilis*

(But there were 44 aggressions, some of them effective. The silverfish could have died in a longer experiment and was not used for long-term test).

Generalist tests (N=34)

G1. With *N. curtiseta* (N=21)

G1.A. *N. curtiseta* with *Aphaenogaster*

Number of experiment (N=2)	SURVIVAL (15´)	Number of ants	Number of Silv	Number of Silv. died	Ant species
S121	100%	6	2	0	*A. iberica*
S122	100%	2	1	0	*A. iberica*

But one silverfish in the test S121 died few hours after 15' test

G.1.B. N. curtiseta with *Camponotus*

Number of experiment (N=11)	SURVIVAL (15')	Number of ants	Number of Silv	Number of Silv. died	Ant species
S253	100%	3	1	0	*C. cruentatus*
S118	100%	1	1	0	*C. pilicornis*
S124	50%	2	4	2	*C. pilicornis*
S130	0%	1	1	1	*C. pilicornis*
S144	100%	2	2	0	*C. pilicornis*
S147	100%	1	2	0	*C. pilicornis*
S148	50%	1	2	0	*C. pilicornis*
S033	0%	1	1	1	*C. pilicornis*
S036	100%	2	3	0	*C. pilicornis*
S052	0%	1	1	1	*C. pilicornis*
S053	100%	1	1	0	*C. sylvaticus*

Survival reduces in smallest recipients

G.1.C. N. curtiseta with *Iberoformica subrufa*

Number of experiment (N=3)	SURVIVAL (15')	Number of ants	Number of Silv	Number of Silv. died
S055	66%	3	3	1
S064	0%	3	1	1
S112a	100%	2	1	0

G.1.D. N. curtiseta with *Messor barbarus*

Number of experiment (N=3)	SURVIVAL (15')	Number of ants	Number of Silv	Number of Silv. died
S008	0%	2	1	1
S020	100%	4	5	0
S058	0%	3	1	1

G.1.E. N. curtiseta with *Cataglyphis hispanica*

Number of experiment (N=1)	SURVIVAL (15')	Number of ants	Number of Silv	Number of Silv. died
S040	0%	4	9	9

G.1.F. N. curtiseta with *Tapinoma nigerrimum*

Number of experiment(N=1)	SURVIVAL (15')	Number of ants	Number of Silv	Number of Silv. died
S115	100%	2	1	0

G.2. With *Proatelurina pseudolepisma* (N=13)

G.3.A. *Proatelurina* with *Camponotus pilicornis*

Number of experiment (N=1)	SURVIVAL (15')	Number of ants	Number of Silv	Number of Silv. died
S105	0%	2	1	1

G.2.B. Proatelurina with *Lasius*

Number of experiment (N=2)	SURVIVAL (15')	Number of ants	Number of Silv	Number of Silv. died	Ant species
S027	0%	3	1	1	*Lasius niger* complex
S112b	100%	2	1	0	*Lasius grandis*

G.2.C. Proatelurina with *Messor barbarus*

Number of experiment (N=1)	SURVIVAL (15')	Number of ants	Number of Silv	Number of Silv. died
S057	0%	3	1	1

G.2.D. Proatelurina with *Pheidole pallidula*

Number of experiment (N=4)	SURVIVAL (15')	Number of ants	Number of Silv	Number of Silv. died
S042	100%	5	1	0
S150	0%	2	2	2
S401	100%	8	1	0
S404	0%	7	1	1

G.2.E. Proatelurina with *Tetramorium forte/ruginodis*

Number of experiment (N=2)	SURVIVAL (15')	Number of ants	Number of Silv	Number of Silv. died
S108	0%	8	1	1
S110	0%	5	1	1

Facultative silverfish tests

F.1. With *Lepisma baetica/chlorosoma* (N=6)

F.1.A. L. baetica with *Tetramorium forte/ruginodis*

Number of experiment (N=5)	SURVIVAL (15')	Number of ants	Number of Silv	Number of Silv. died

S109	0%	3	1	1
S113	100%	2	1	0
S116	100%	3	2	0
S143	100%	5	3	0
S405	100%	8	2	0

F.1.B. L.baetica with *Messor barbarus*

**Number of experiment (N=1)**	**SURVIVAL (15')**	**Number of ants**	**Number of Silv**	**Number of Silv. died**
S030	0%	1	1	1

Non-myrmecophile (xenomyrmecophile) tests (N=9; N =7 discarding species of other orders of Hexapoda)

**Number of experiment**	**SURVIVAL (15')**	**Number of ants**	**Number of Silv (or another)**	**Number of Silv. (or another) died**
S266 *Aphaenogaster senilis / Ctenolepisma nicoletii*	0%	2	1	1
S006 *Messor barbarus / C. ciliatum*	0%	3	1	1
S028 *Messor barbarus / C. ciliatum*	0%	5	1	1
S031 *Messor barbarus / C. ciliatum*	0%	3	1	1
S257 *Messor barbarus / C. targionii*	0%	2	1	1
S264 *Messor barbarus / C. nicoletii*	0%	2	1	1
S050 *Pheidole pallidula / C. nicoletii*	0%	5	1	1
S007 *Messor barbarus / Diplura Campodeidae*	0%	3	1	1
S101 *Messor barbarus / Microcoryphia Machilidae*	0%	1	1	1

In smaller recipients, silverfish (or another insect) were killed more quickly.
