# Supplementary material for: Chemical and behavioural strategies along the spectrum of host specificity in ant-associated silverfish
Source: BMC Zool. 2022 May 11;7:23. doi: 10.1186/s40850-022-00118-9 (PMC10127367; doi:10.1186/s40850-022-00118-9)
Supplement: Supplementary file 20 — Additional file 20. Exemplary chromatograms of silverfish and corresponding host species. NMDS plots based on the Bray-Curtis distances of the shared CHC peaks are also given for ant-silverfish pairs. [file 40850_2022_118_MOESM20_ESM.docx]

Exemplary chromatograms of silverfish and corresponding host species. NMDS plots based on the Bray-Curtis similarities of the shared CHC peaks are also given for ant-silverfish pairs.

Silverfish species are represented in the NMDS plots by grey colored circles (moulted individuals by blue colored circles) around a letter code. Host ants are depicted by a letter code without colored circle. The letter code refers to the host colony (see Table 3).

Test results of PERMANOVA (test differences in host and silverfish CHC composition, number of unique permutations: if number >999, 999 permutations were run), PERMDISP (test differences in CHC profile variation between host and silverfish) and Mean BC similarity ± SD of silverfish to the host ant are given under the NMDS plots.


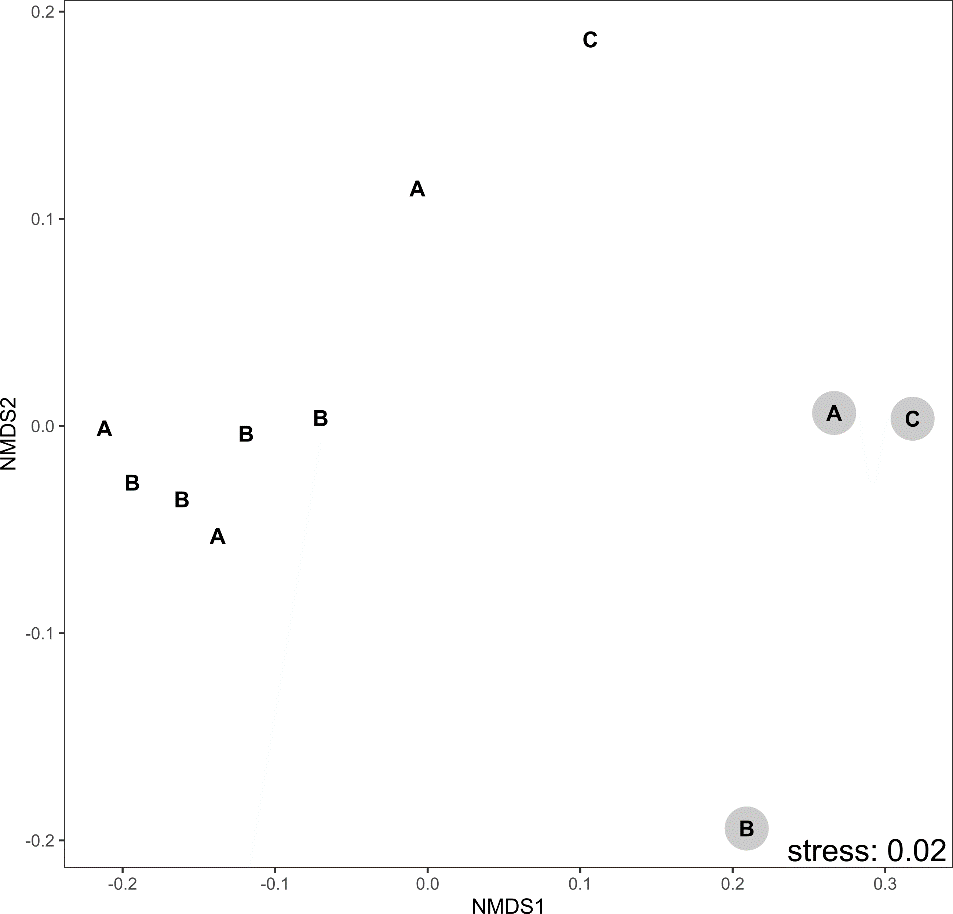

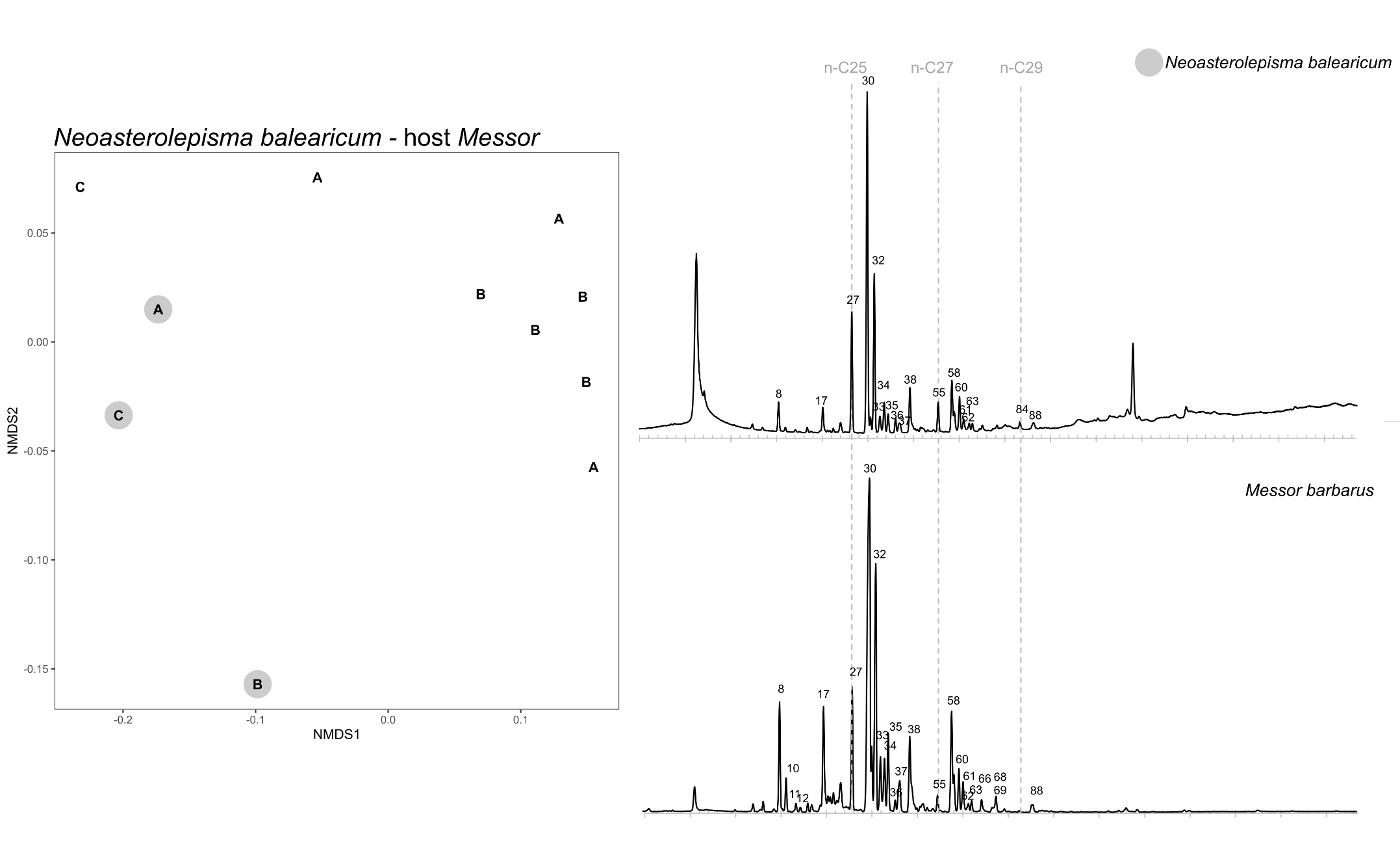
***Messor* specialists**

*N. balearicum*


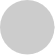


PERMANOVA, Pseudo-F = 13.9, *P* = 0.049 (40 permutations)

PERMDISP, F = 0.0, *P =* 0.912

BC similarity to the ant host ± SD = 0.57 ± 0.05

*Messor barbarus*

*Neoasterolepisma balearicum –* host *Messor*


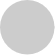

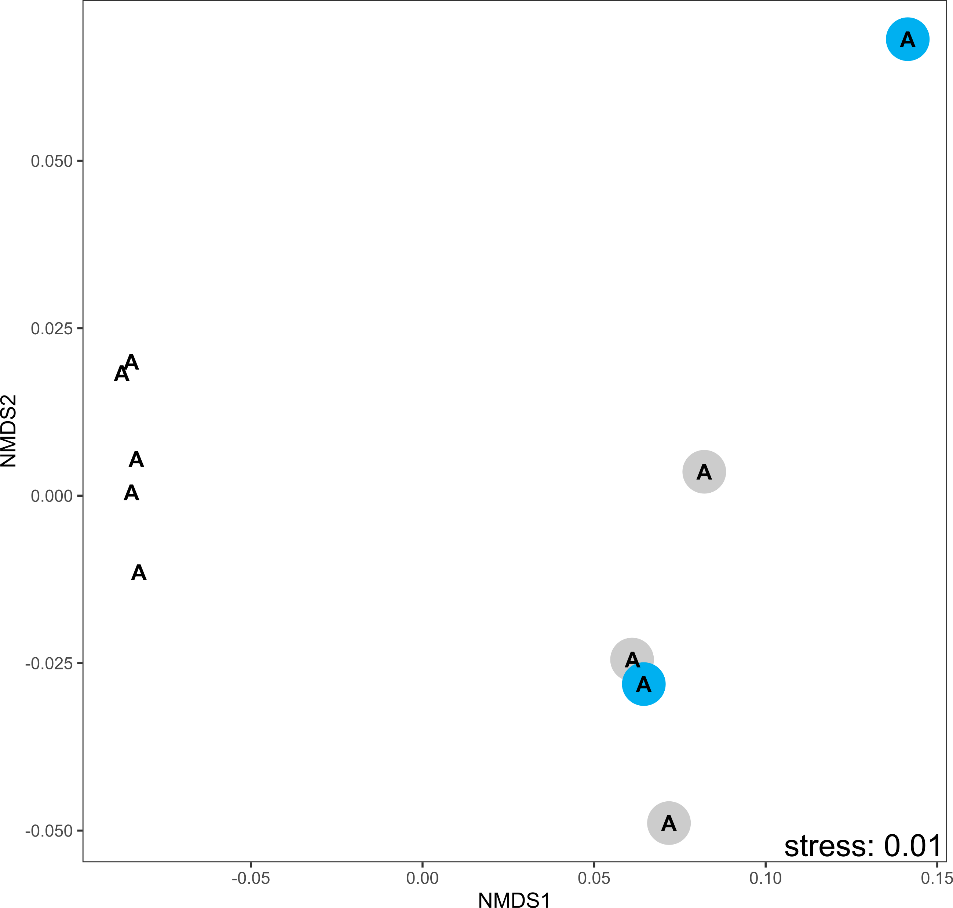

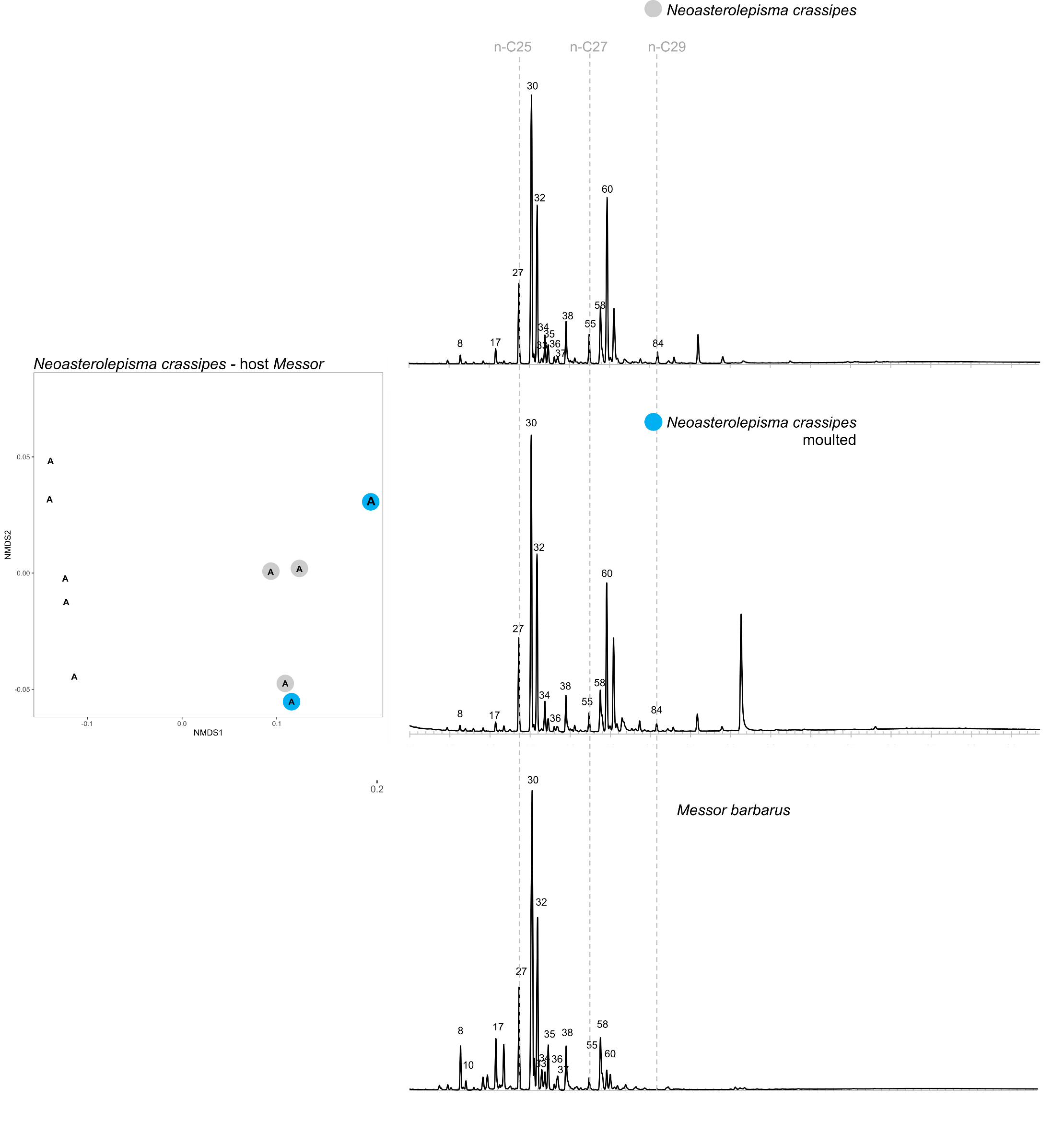

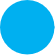

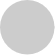


Associated silverfish vs host species

PERMANOVA, Pseudo-F = 54.1, *P* = 0.035 (56 permutations)

PERMDISP, F = 3.1, *P =* 0.128

BC similarity to the ant host ± SD = 0.71 ± 0.01

Moulted silverfish vs host species

BC similarity to the ant host ± SD = 0.66 ± 0.08

*Neoasterolepisma crassipes* – host *Messor*

*N. crassipes*

moulted

*N. crassipes*

*Messor barbarus*


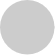

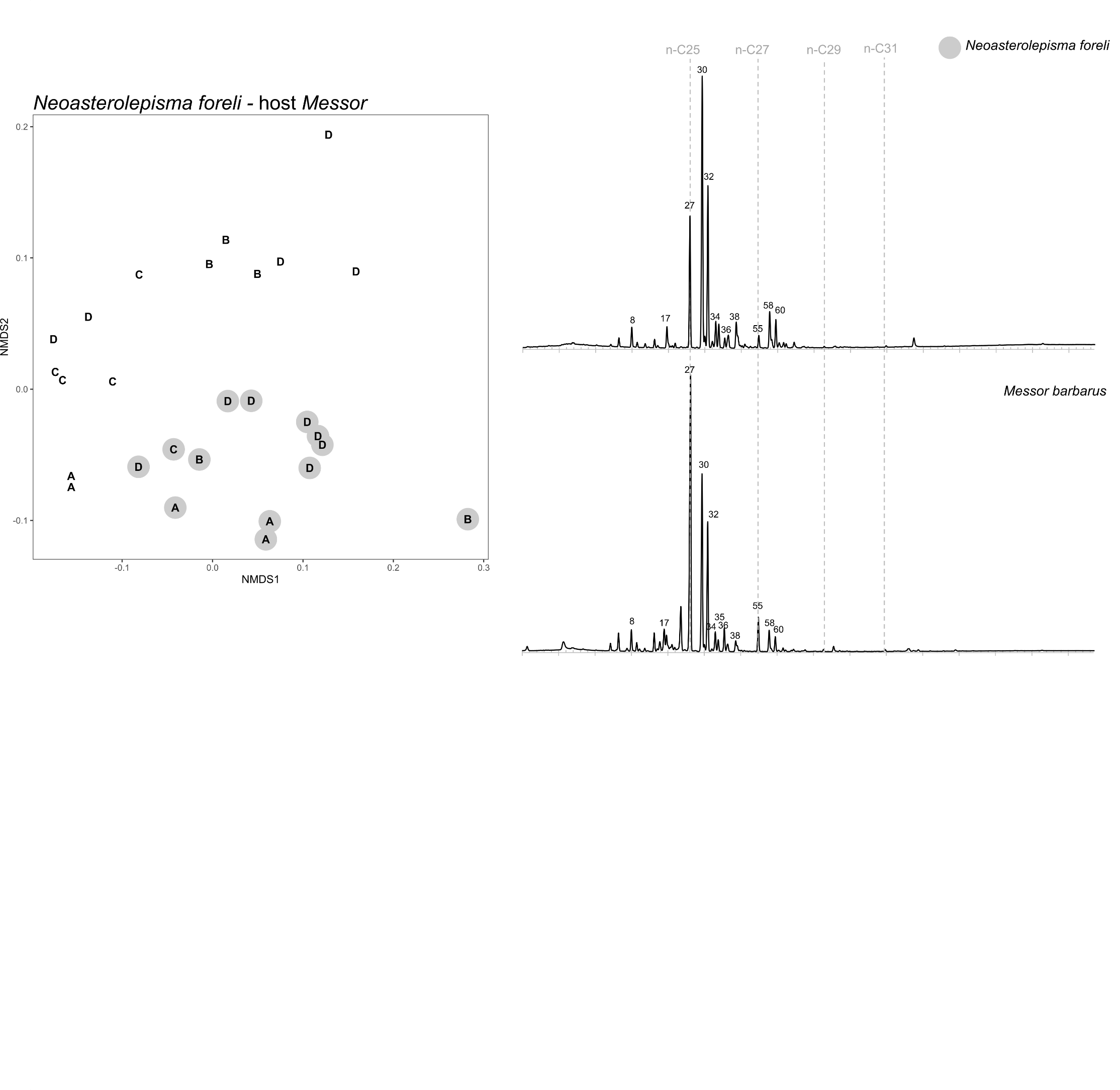

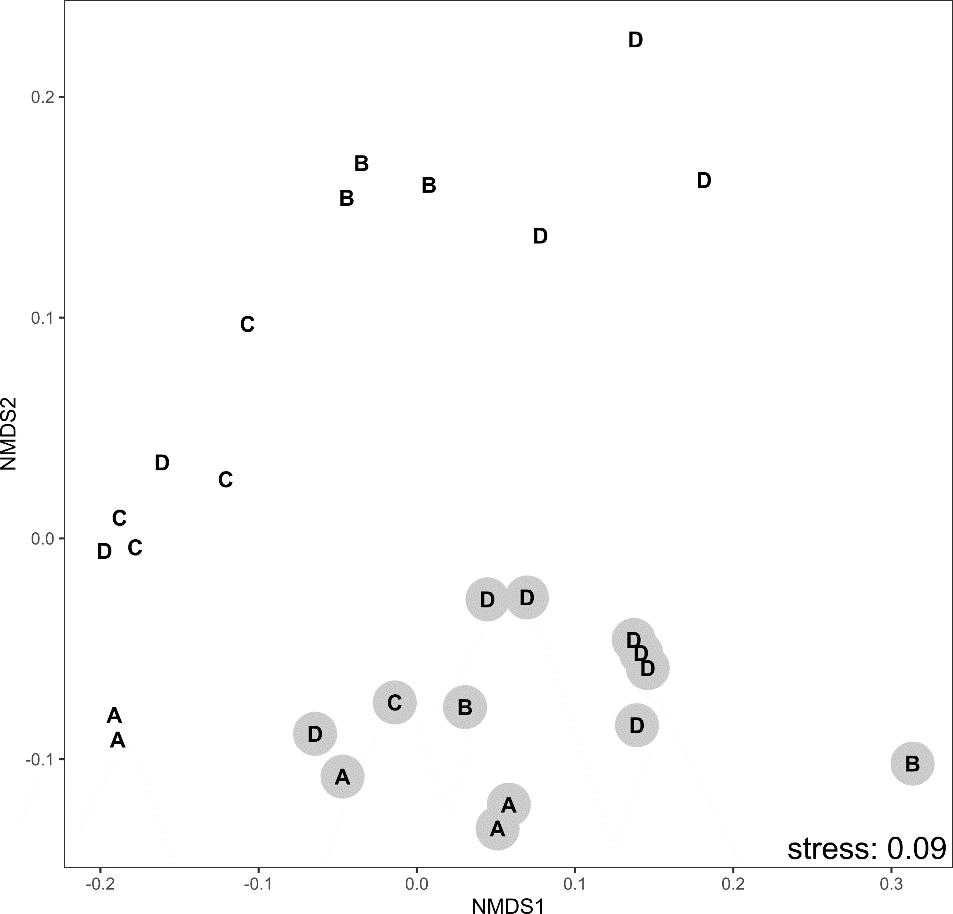


*Neoasterolepisma foreli* – host *Messor*

*Messor barbarus*

*N. foreli*

PERMANOVA, Pseudo-F = 12.6, *P* = 0.001 (999 permutations)

PERMDISP, F = 3.4, *P =* 0.075

BC similarity to the ant host ± SD = 0.72 ± 0.06


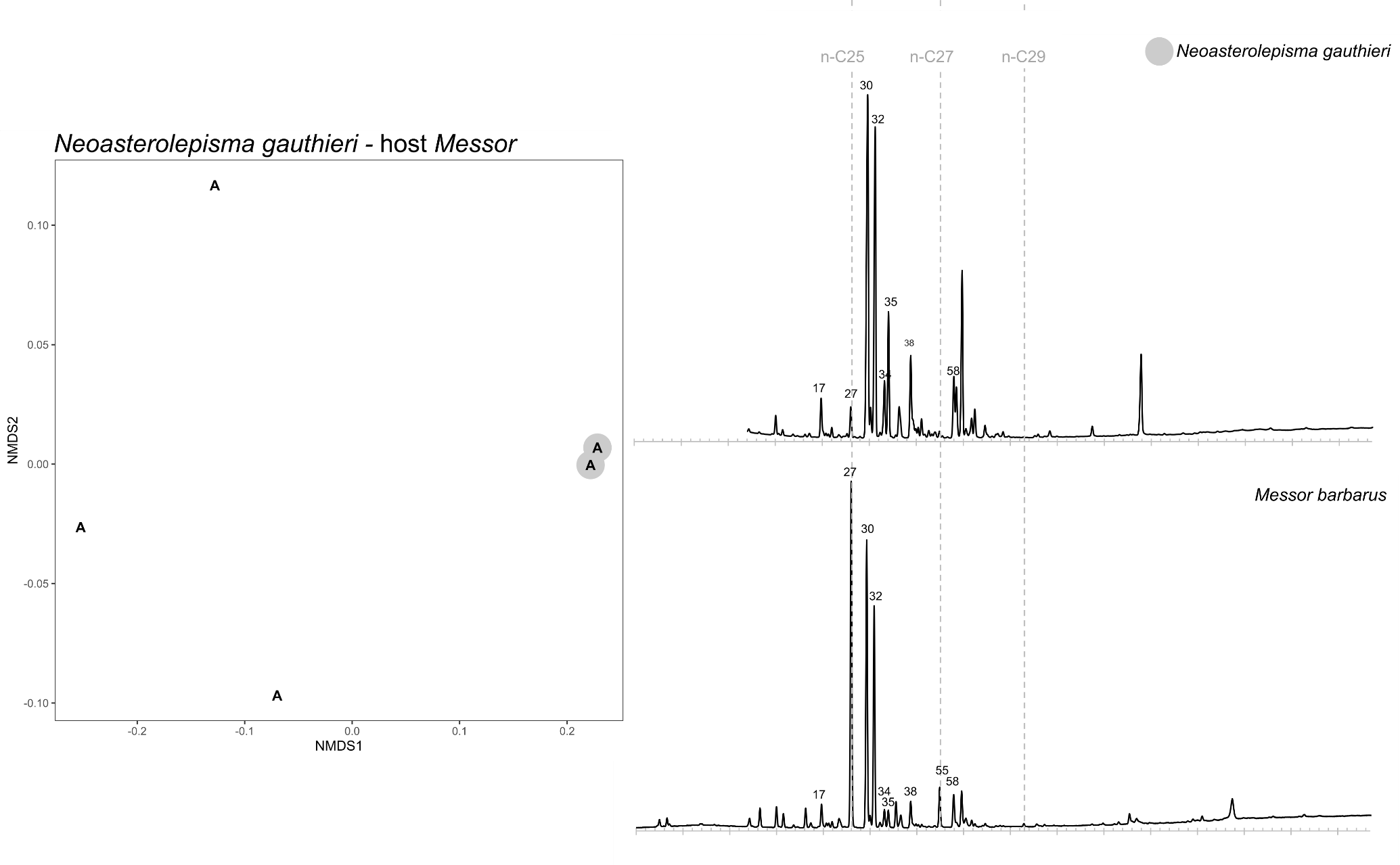

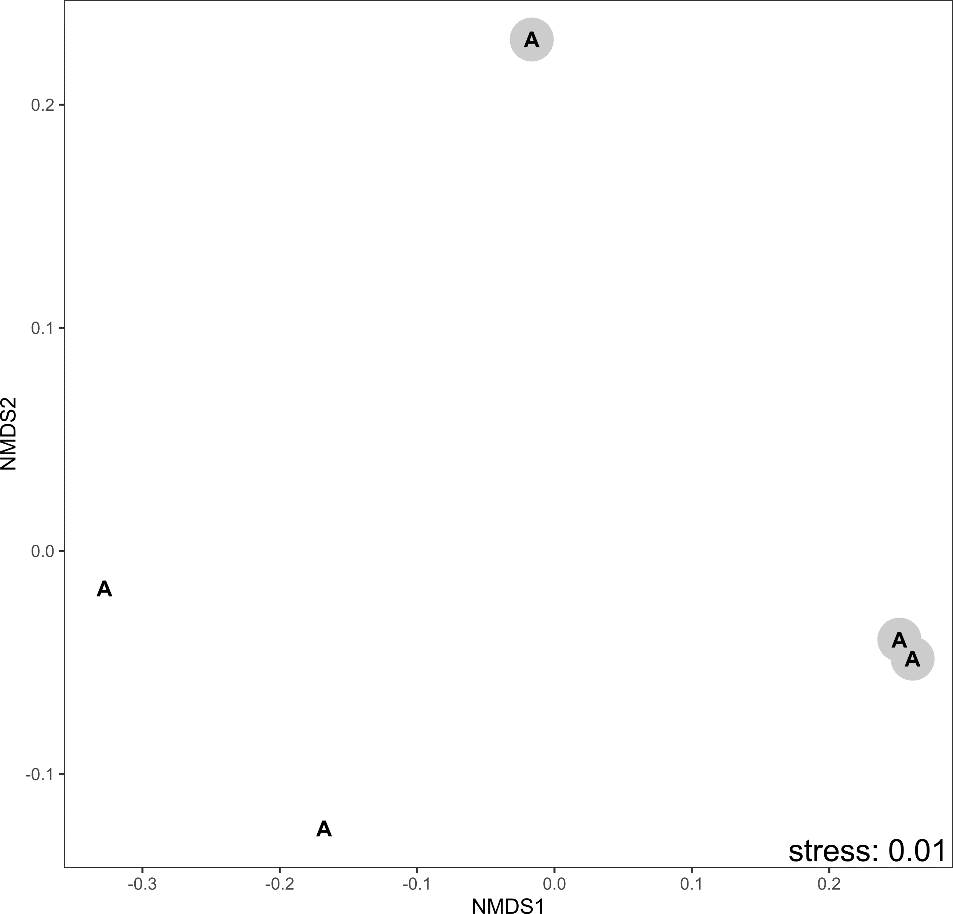


*N. gauthieri*

*calvum*

*Neoasterolepisma gauthieri calvum* – host *Messor*


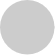


PERMANOVA, Pseudo-F = 5.8, *P* = 0.189 (10 permutations)

PERMDISP, F = 0.1, *P =* 0.729

BC similarity to the ant host ± SD = 0.53 ± 0.07

*Messor barbarus*

*Tricholepisma aureum* – host *Messor*


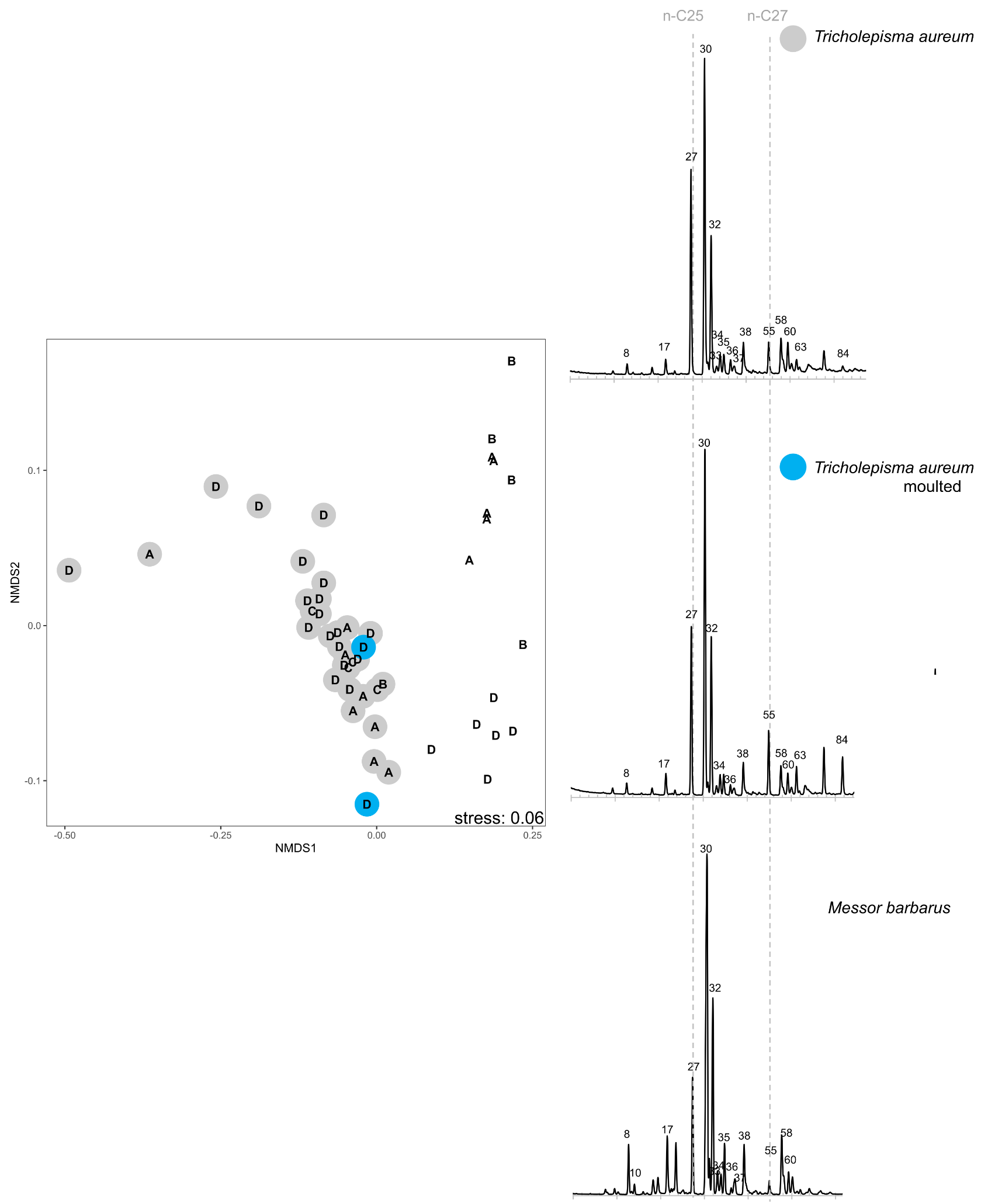


*T. aureum*


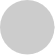


Moulted silverfish (colony D) vs associated silverfish (colony D)

PERMANOVA, Pseudo-F = 2.0, *P* = 0.126 (190 permutations)

PERMDISP, F = 0.5, *P =* 0.510

Associated silverfish vs host species

PERMANOVA, Pseudo-F = 46.7, *P* = 0.001 (999 permutations)

PERMDISP, F = 3.1, *P =* 0.128

BC similarity to the ant host ± SD = 0.68 ± 0.10

only colony D: BC similarity to the ant host colony D ± SD = 0.68 ± 0.11

Moulted silverfish (colony D) vs host species (only colony D)

PERMANOVA, Pseudo-F = 15.8, *P* = 0.103 (28 permutations)

PERMDISP, F = 0.0, *P =* 0.974

BC similarity to the ant host colony D ± SD = 0.77 ± 0.01

*Messor barbarus*

*T. aureum*

moulted


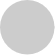

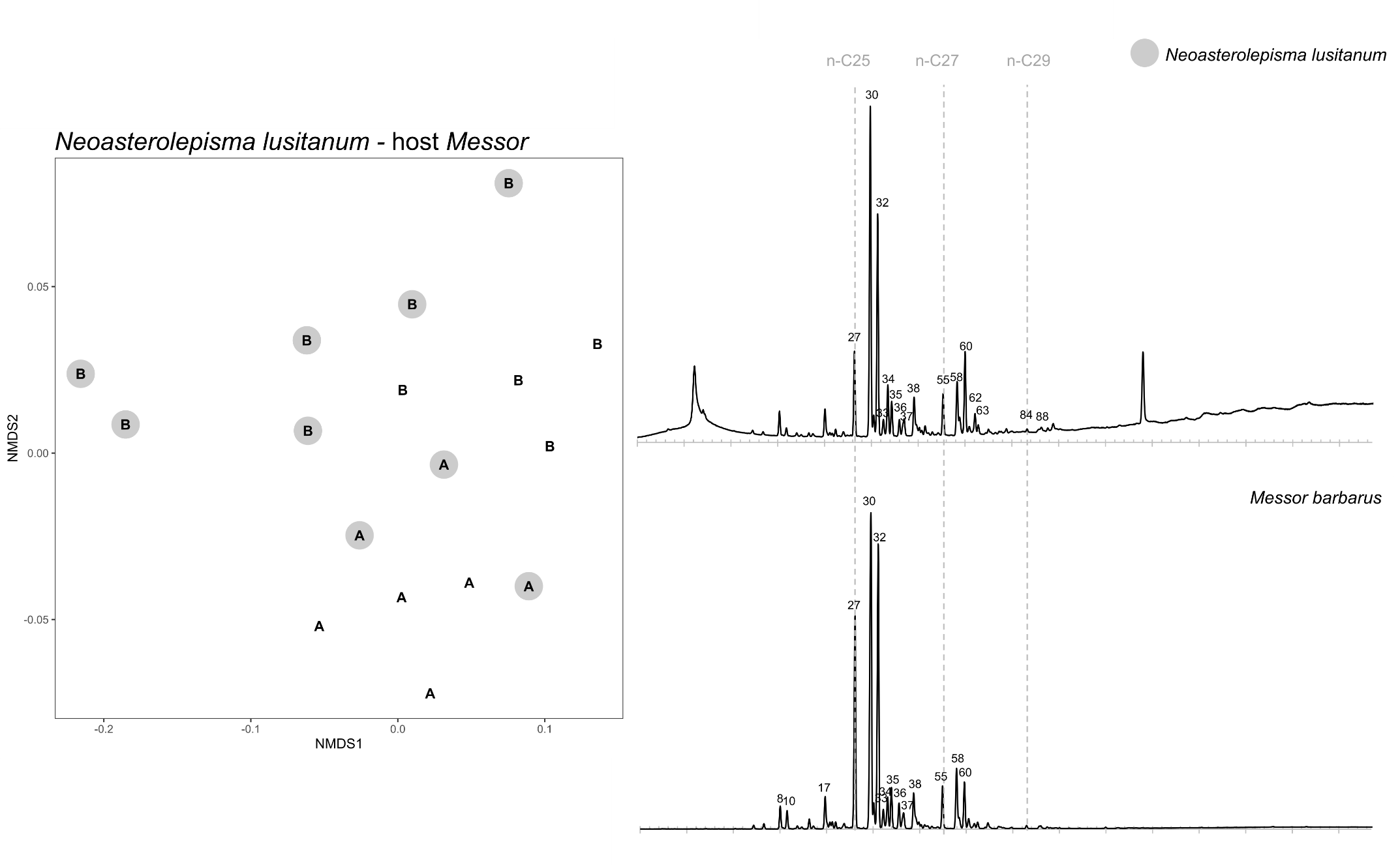

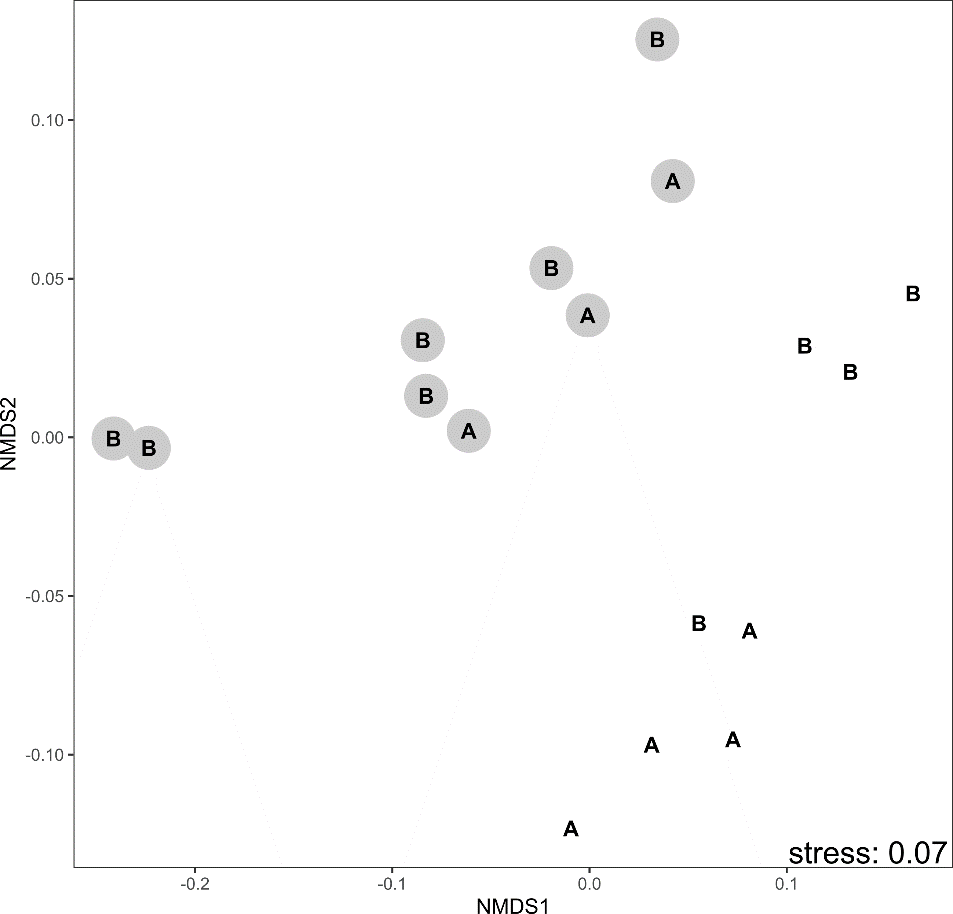


*Neoasterolepisma lusitanum* – host *Messor*

*Messor barbarus*

*N. lusitanum*

PERMANOVA, Pseudo-F = 10.3, *P* = 0.001 (999 permutations)

PERMDISP, F = 0.5, *P =* 0.510

BC similarity to the ant host ± SD = 0.77 ± 0.08


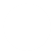

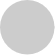

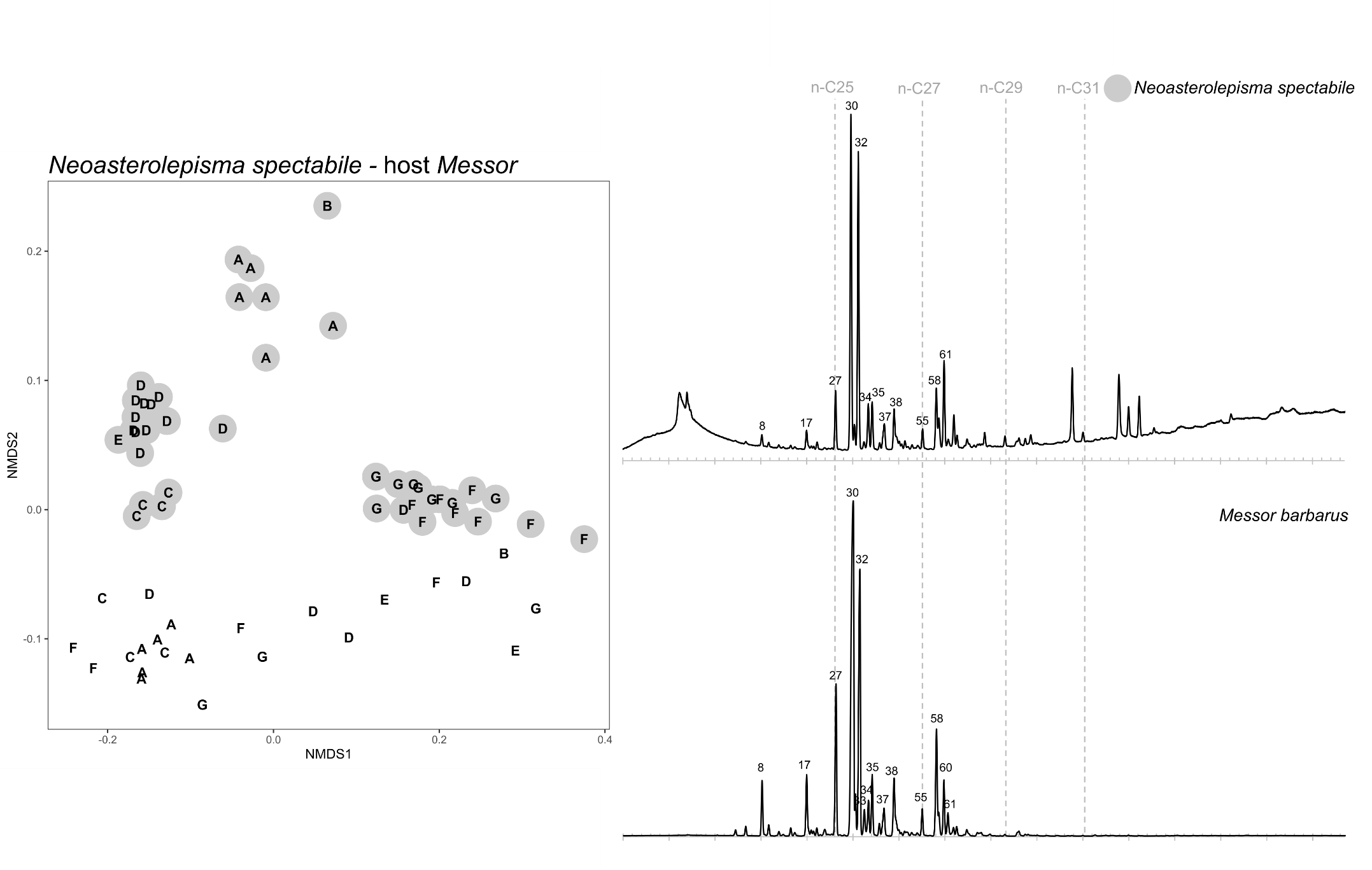

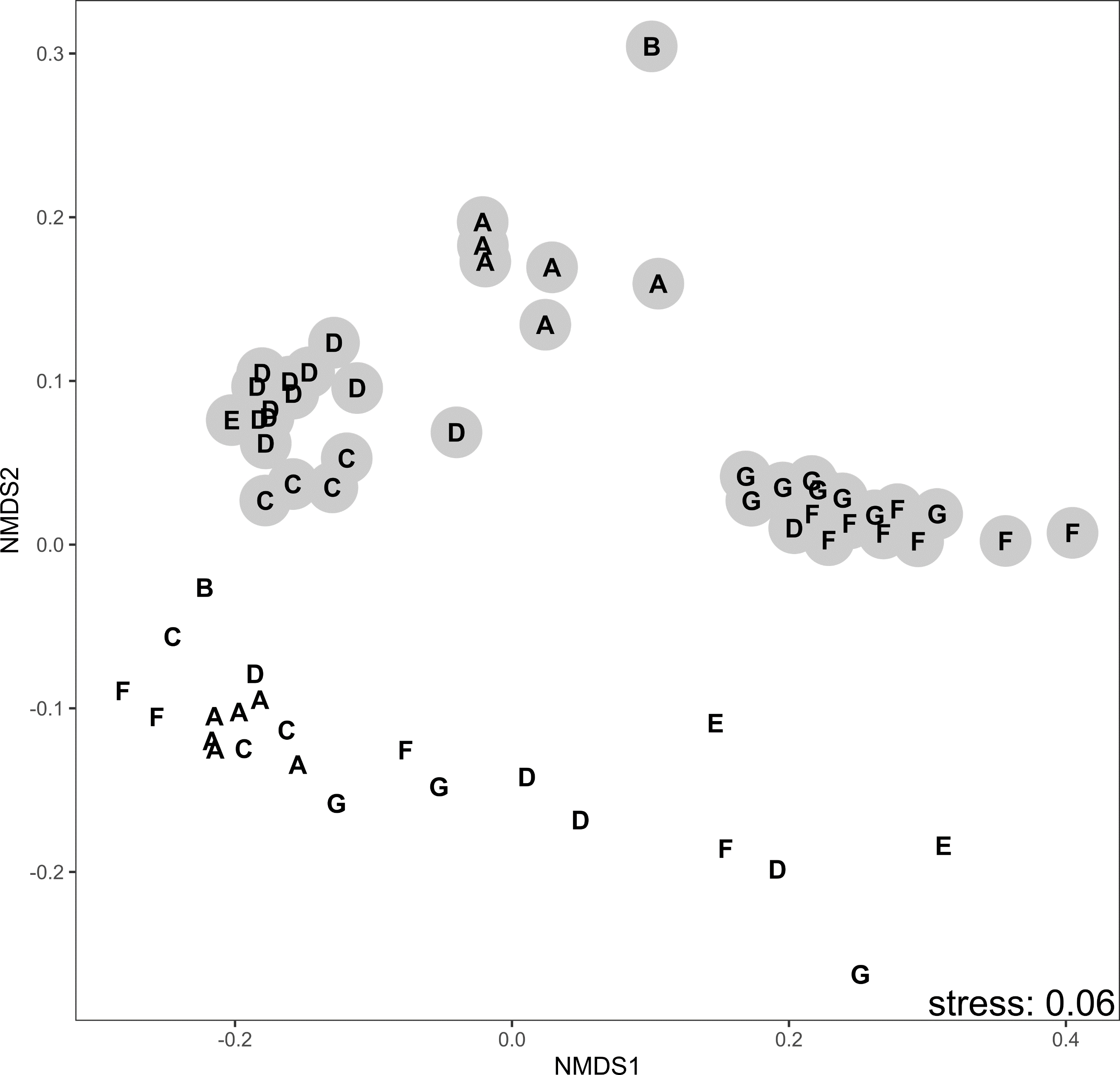


PERMANOVA, Pseudo-F = 19.0, *P* = 0.001 (999 permutations)

PERMDISP, F = 3.1, *P =* 0.083

BC similarity to the ant host ± SD = 0.62 ± 0.08

*N. spectabile*

*Messor barbarus*

*Neoasterolepisma spectabile* – host *Messor*


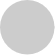

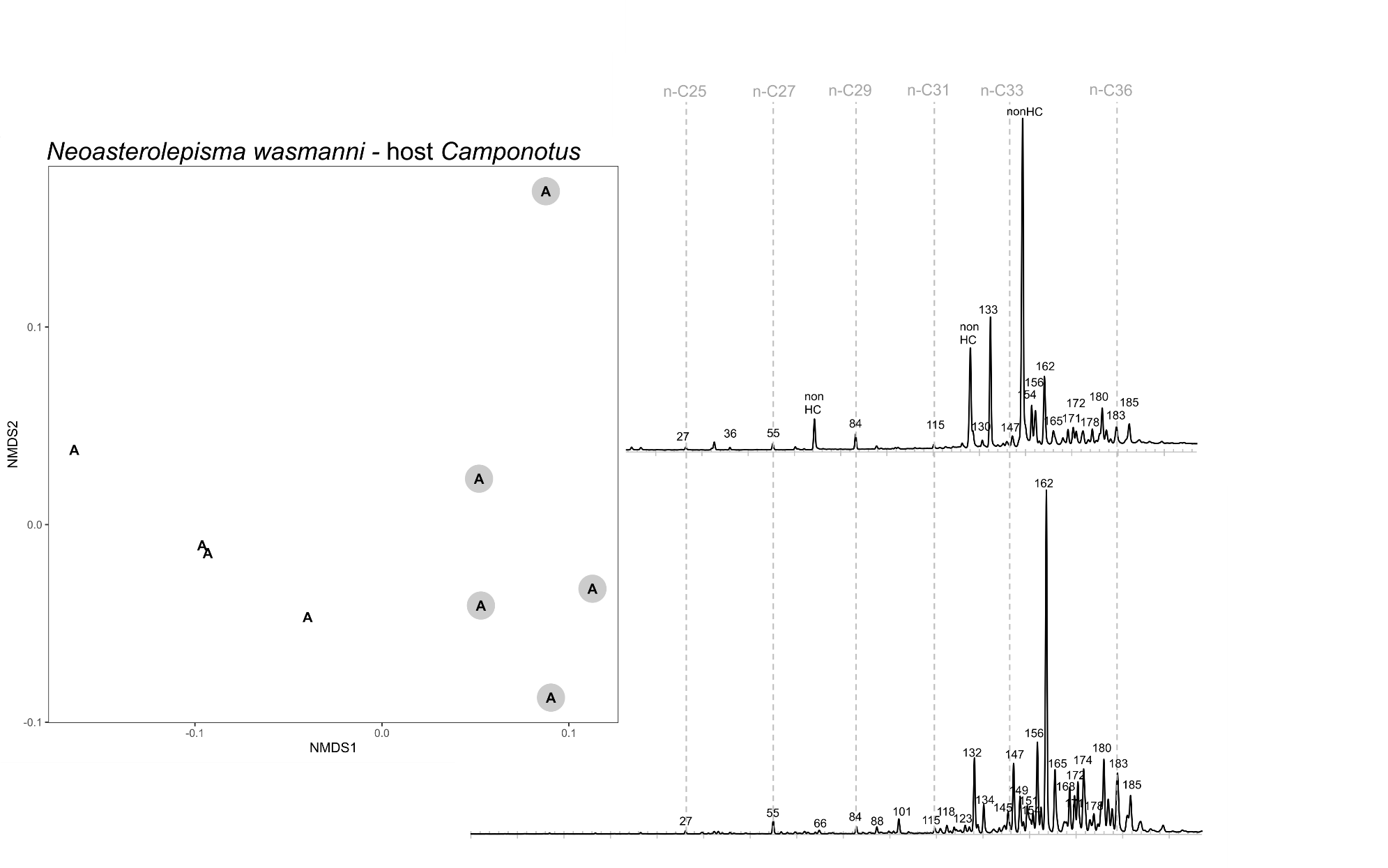

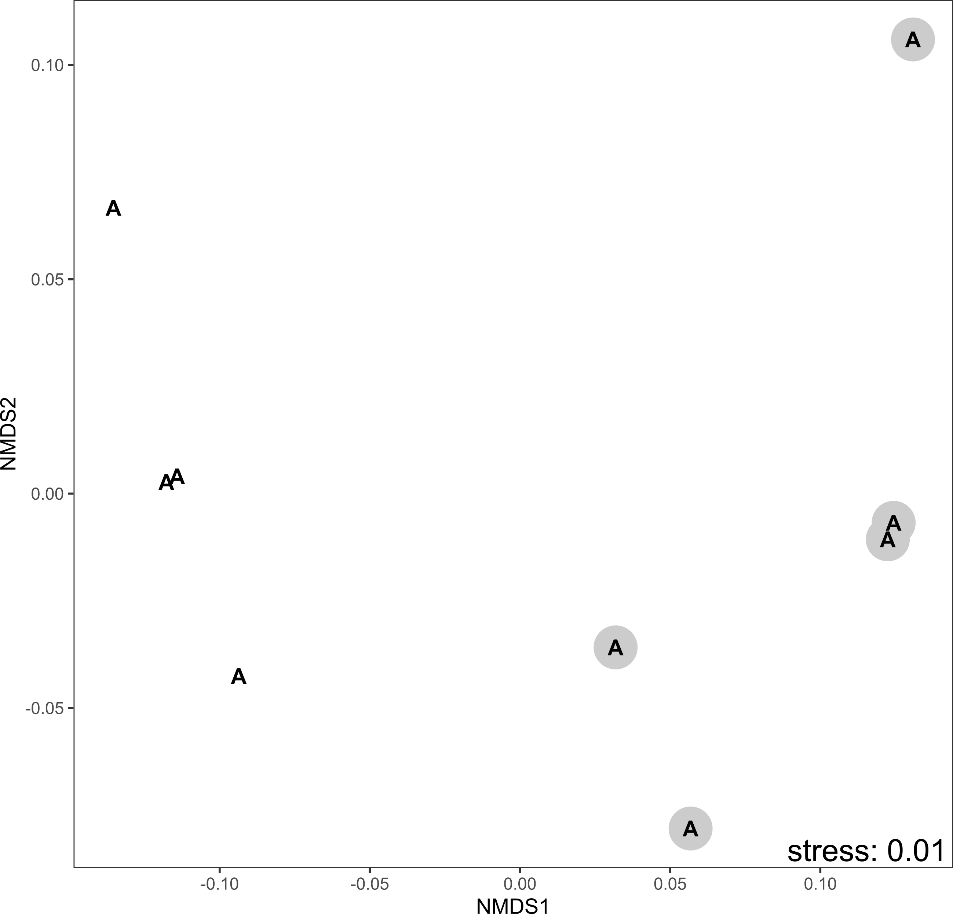


*N. wasmanni*

*Camponotus cruentatus*

PERMANOVA, Pseudo-F = 15.3, *P* = 0.016 (126 permutations)

PERMDISP, F = 1.5, *P =* 0.260

BC similarity to the ant host ± SD = 0.53 ± 0.03

*Neoasterolepisma wasmanni* – host *Camponotus*

***Camponotus* specialists**


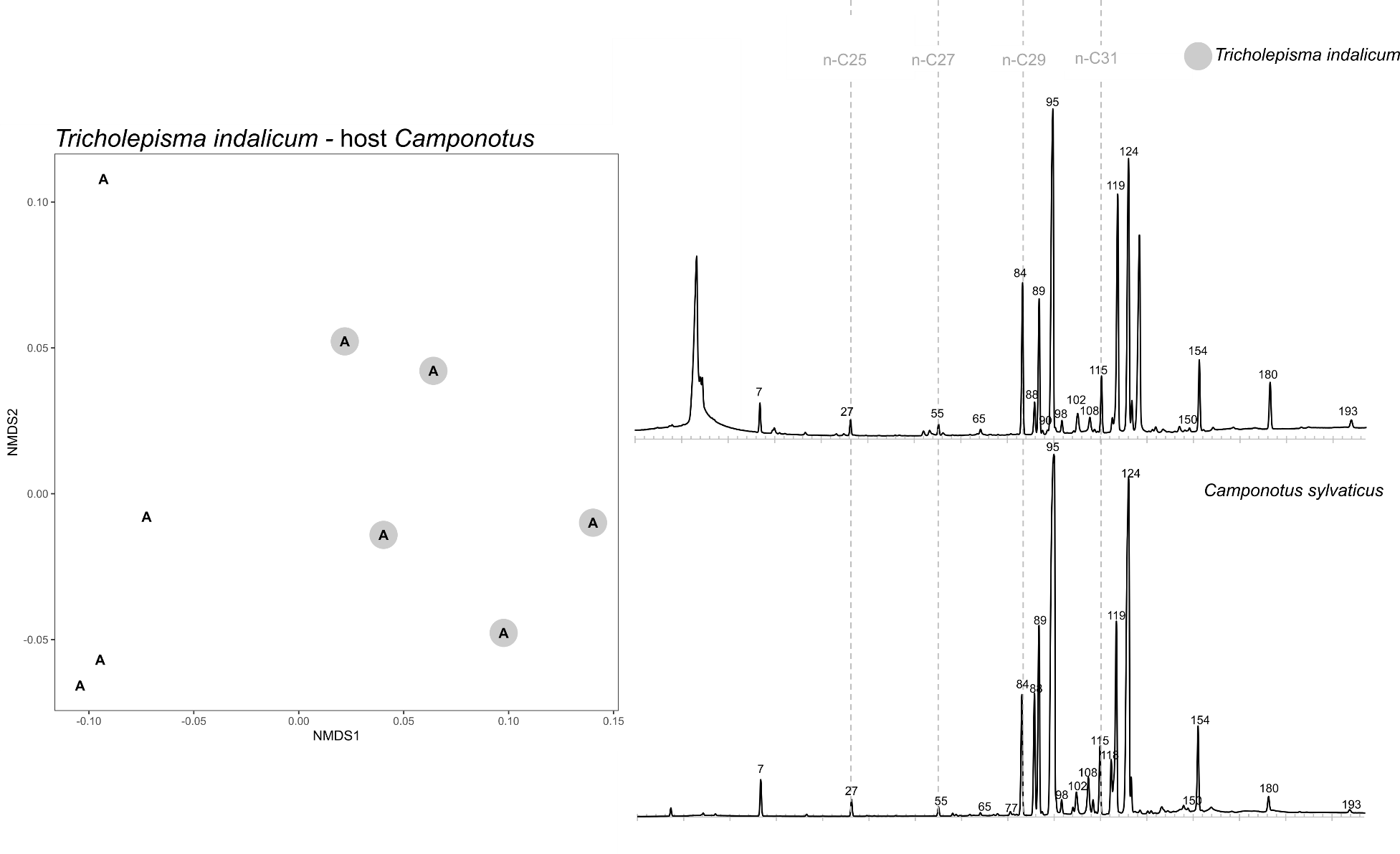


*Tricholepisma indalicum*


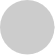


*Camponotus sylvaticus*


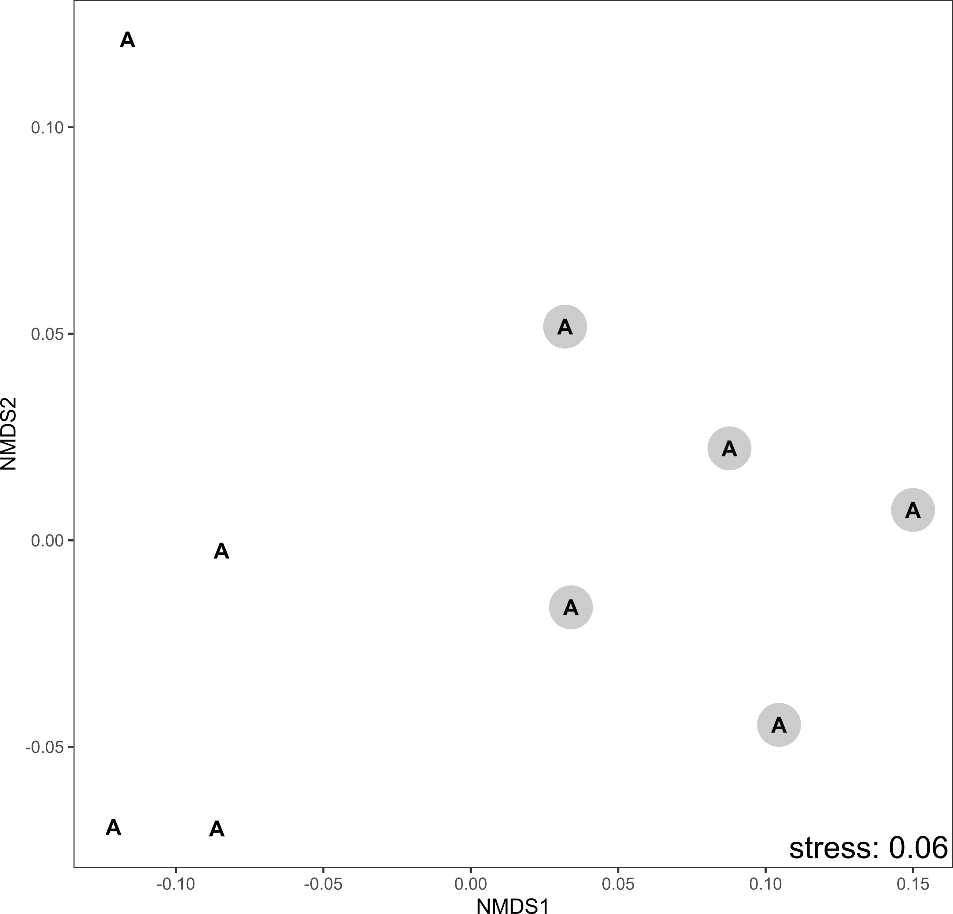


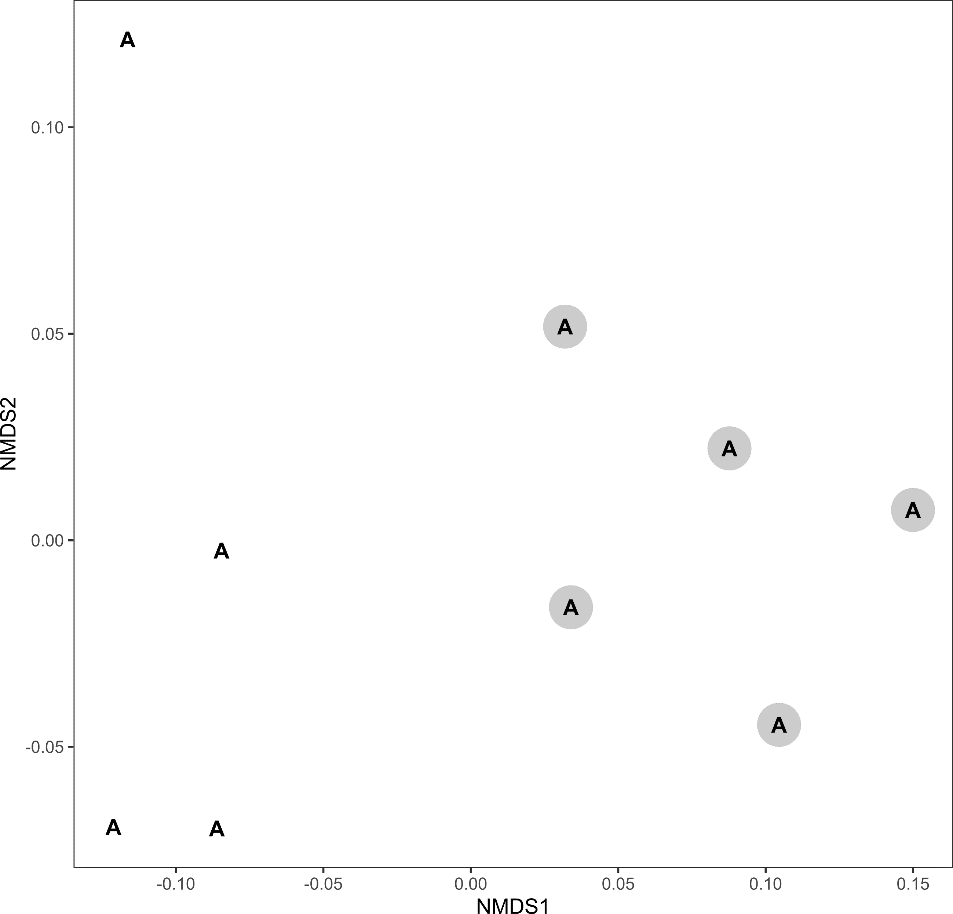


*Tricholepisma indalicum* – host *Camponotus*

PERMANOVA, Pseudo-F = 8.4, *P* = 0.016 (126 permutations)

PERMDISP, F = 0.0, *P =* 0.966

BC similarity to the ant host ± SD = 0.79 ± 0.03

***Aphaenogaster* specialists**


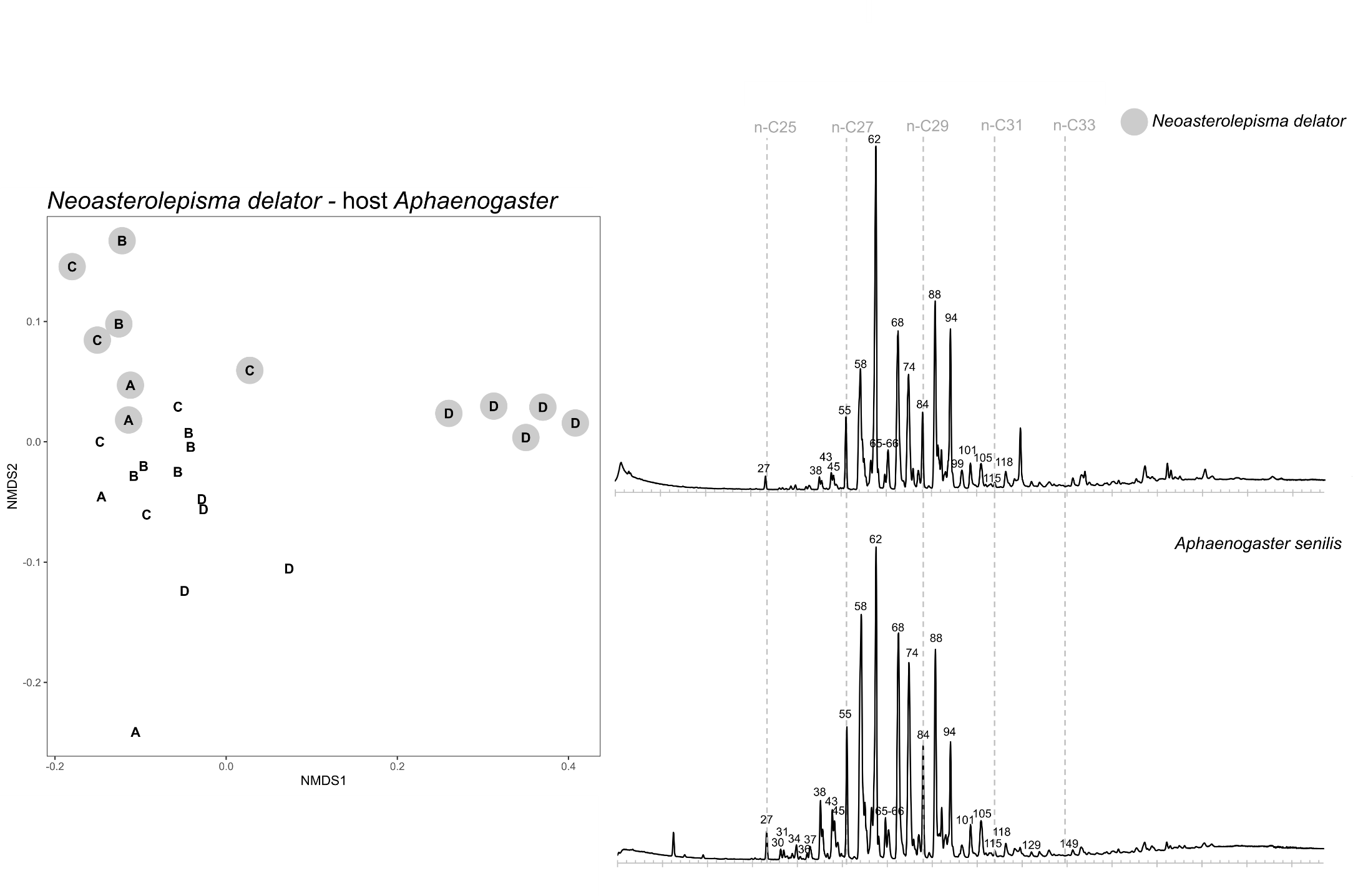


*N. delator*


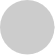


*Aphaenogaster*

*senilis*


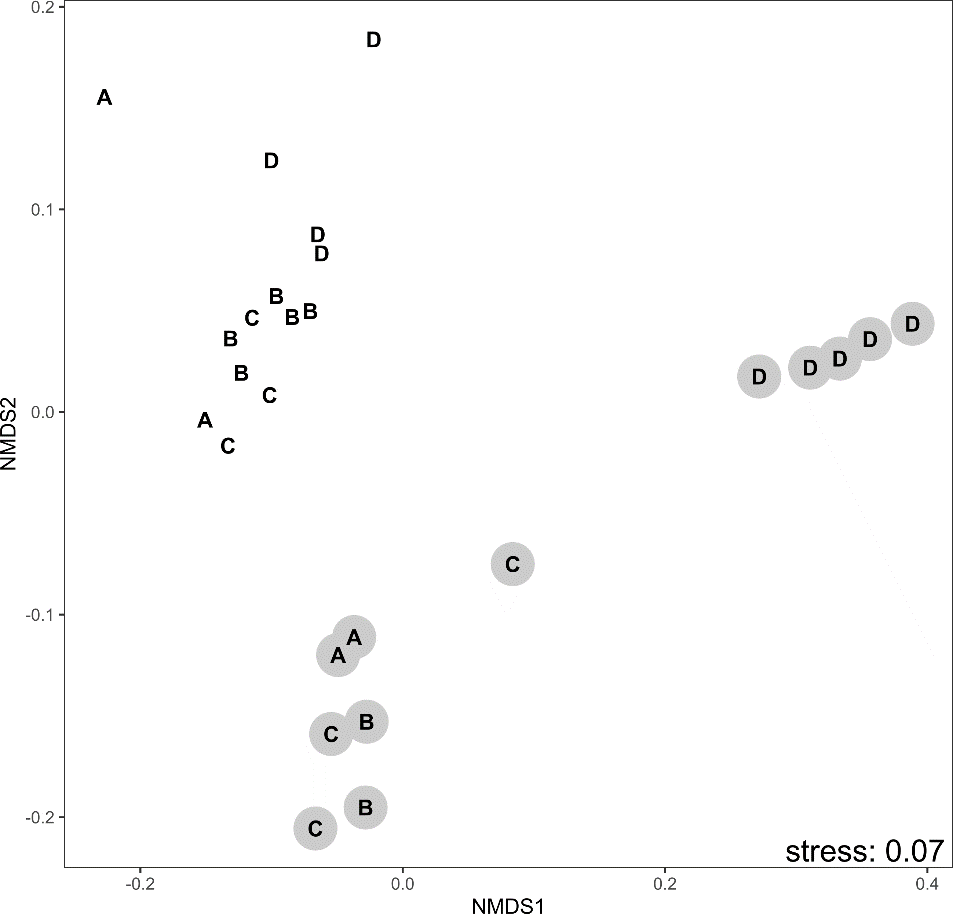


*Neoasterolepisma delator* – host *Aphaenogaster*

PERMANOVA, Pseudo-F = 14.9, *P* = 0.001 (999 permutations)

PERMDISP, F = 12.9, *P* < 0.001

BC similarity to the ant host ± SD = 0.62 ± 0.10


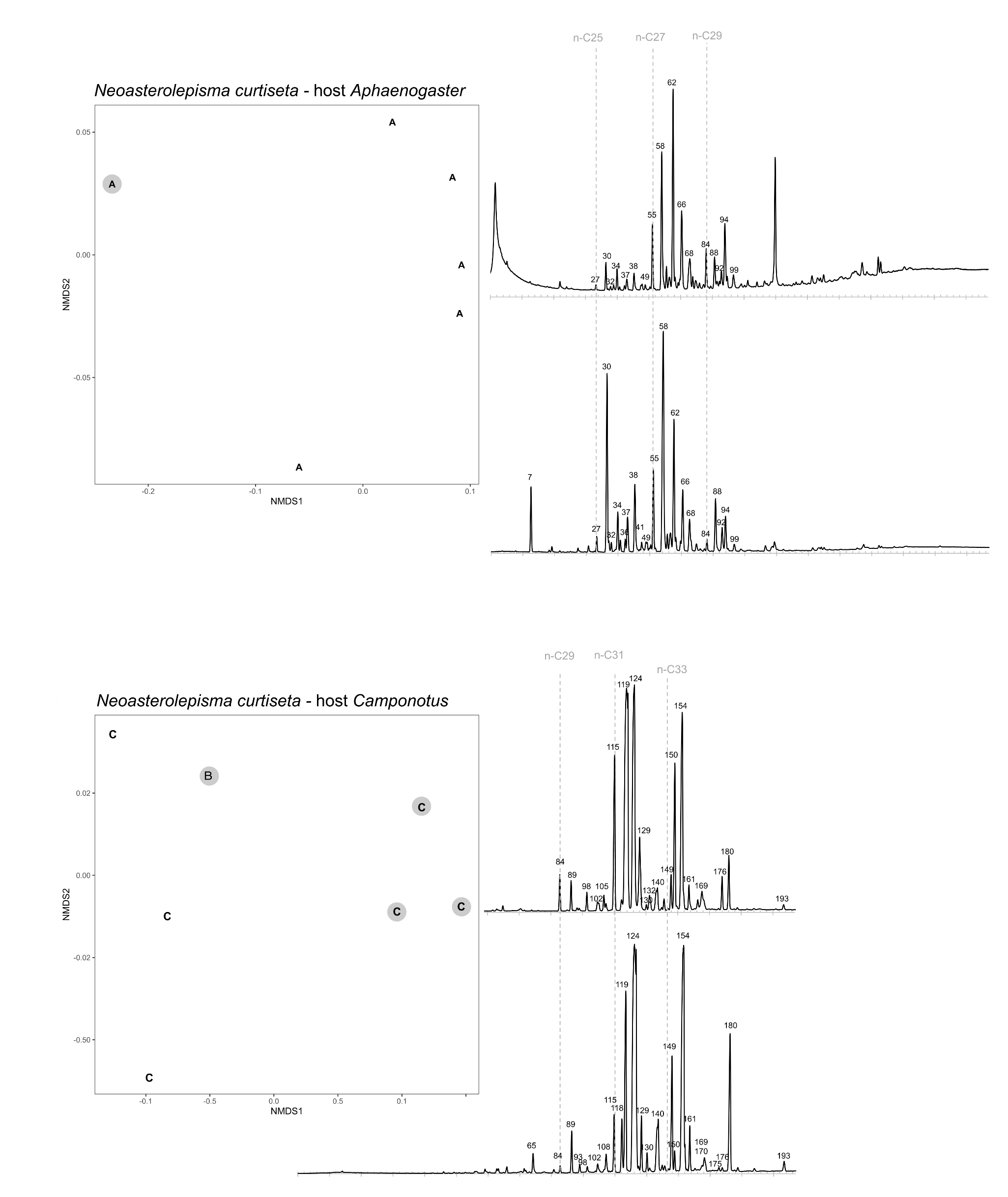

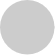

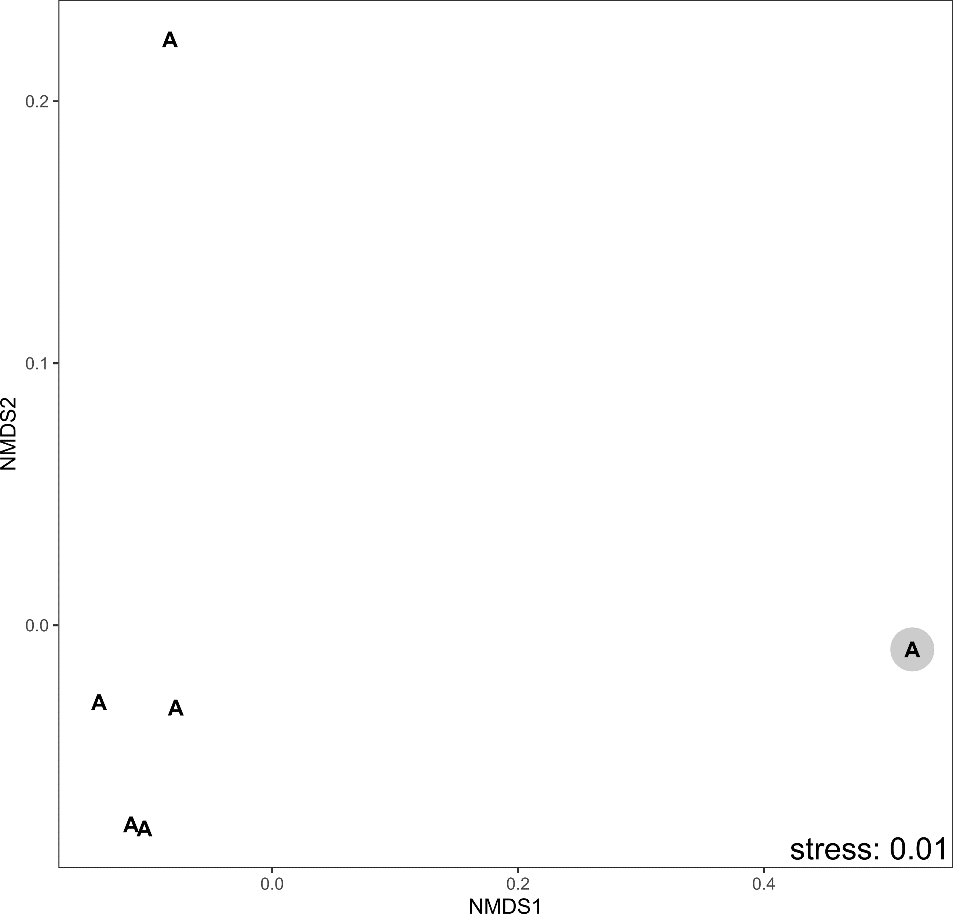
**Host generalists**

PERMANOVA, Pseudo-F = 15.7, *P* = 0.286 (6 permutations)

PERMDISP, F = 1.3, *P =* 0.325

BC similarity to the ant host = 0.36

*N. curtiseta*


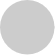

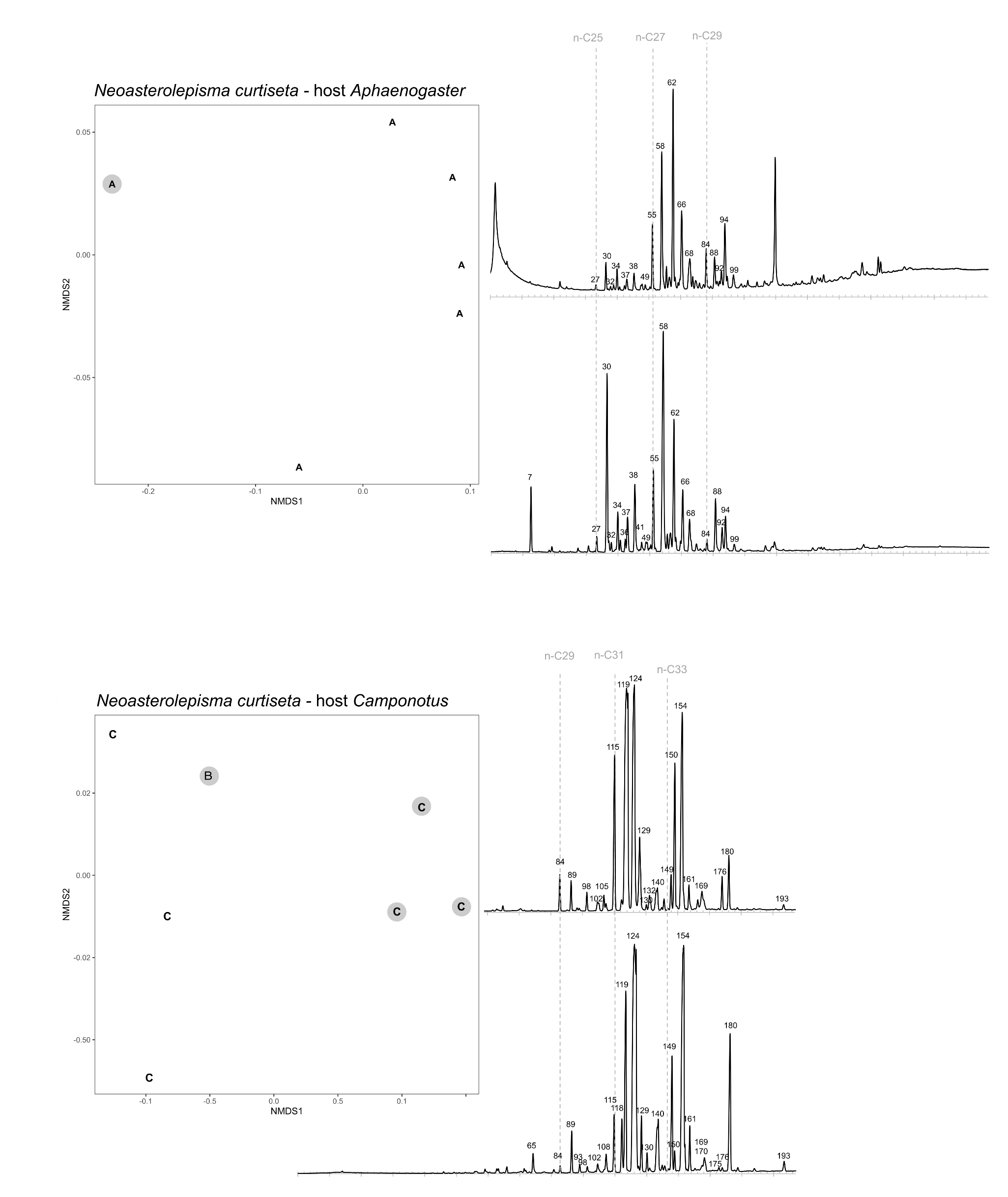

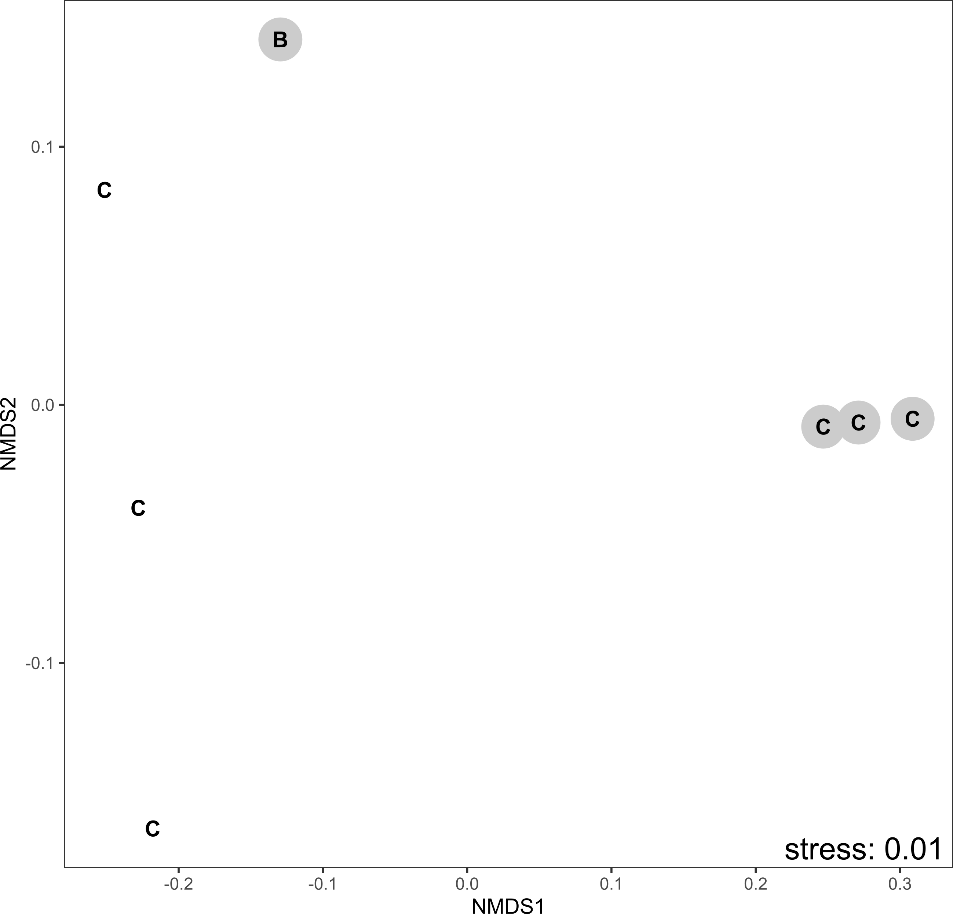


*Camponotus pilicornis*

*N. curtiseta*

*Aphaenogaster iberica*

PERMANOVA, Pseudo-F = 7.7, *P* = 0.056 (35 permutations)

PERMDISP, F = 0.1, *P =* 0.813

BC similarity to the ant host ± SD = 0.48 ± 0.03

*Neoasterolepisma curtiseta* – host *Camponotus*

*Neoasterolepisma curtiseta* – host *Aphaenogaster*


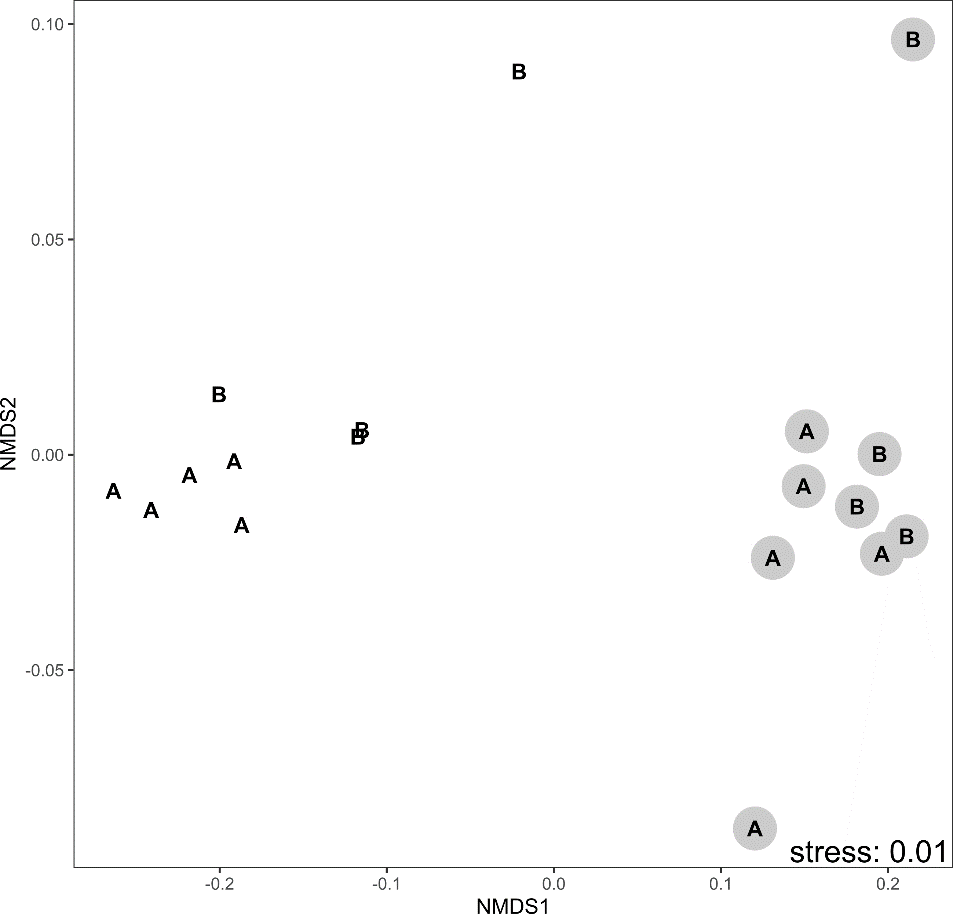

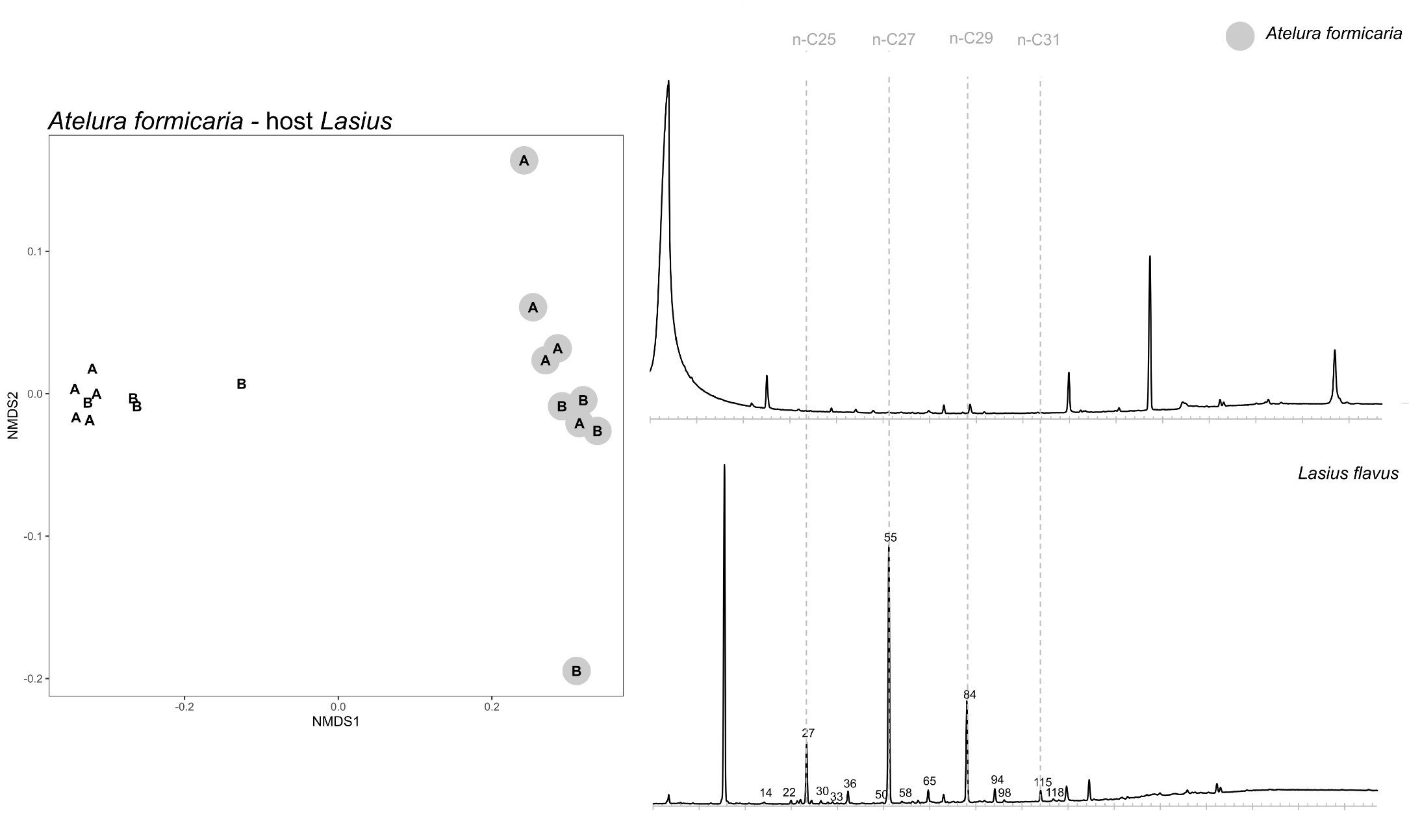

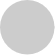


*Atelura formicaria*

*Lasius flavus*

PERMANOVA, Pseudo-F = 118.8, *P* = 0.001 (999 permutations)

PERMDISP, F = 0.2, *P =* 0.703

BC similarity to the ant host ± SD = 0.33 ± 0.02

*Atelura formicaria* – host *Lasius*


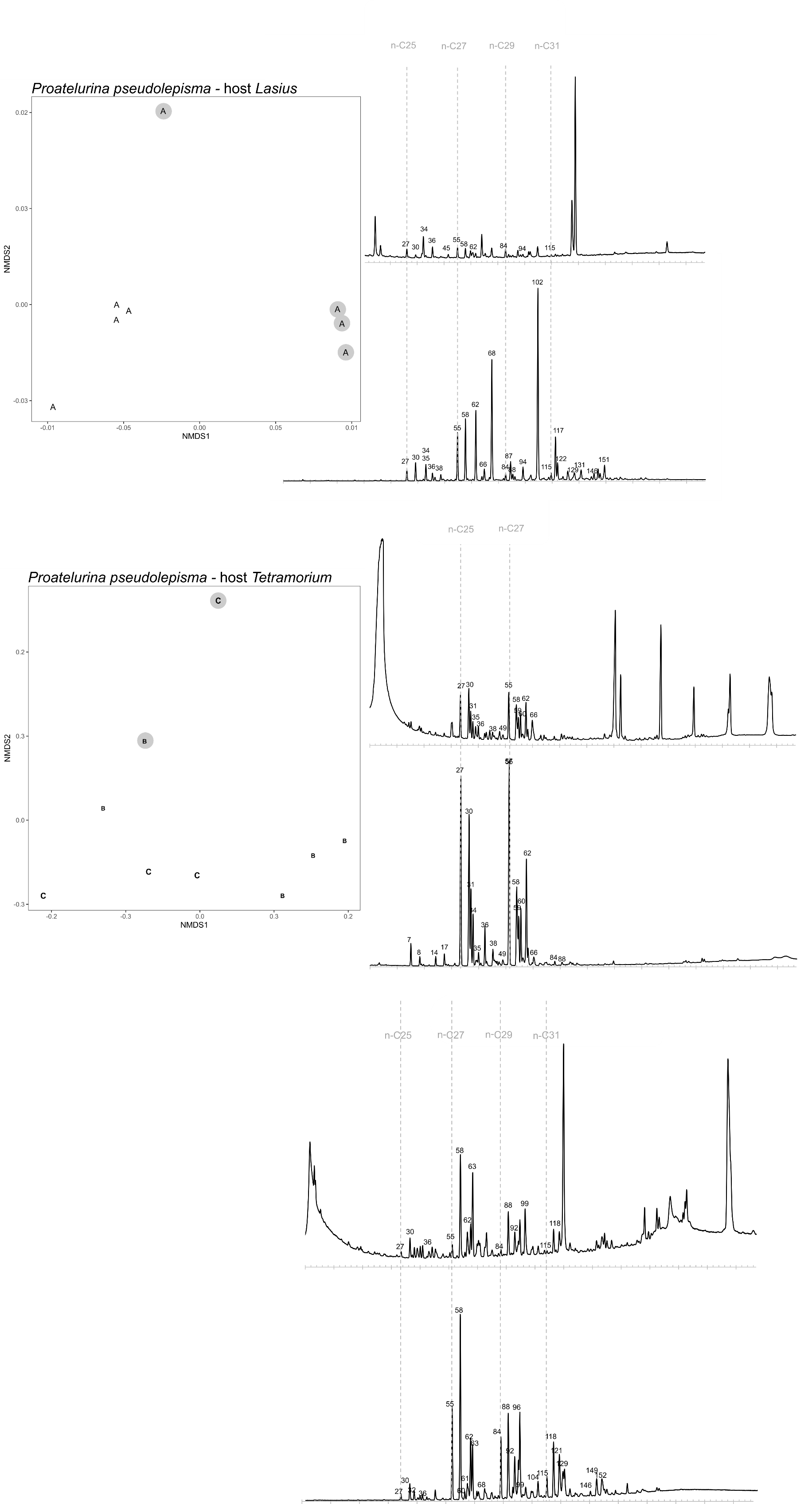

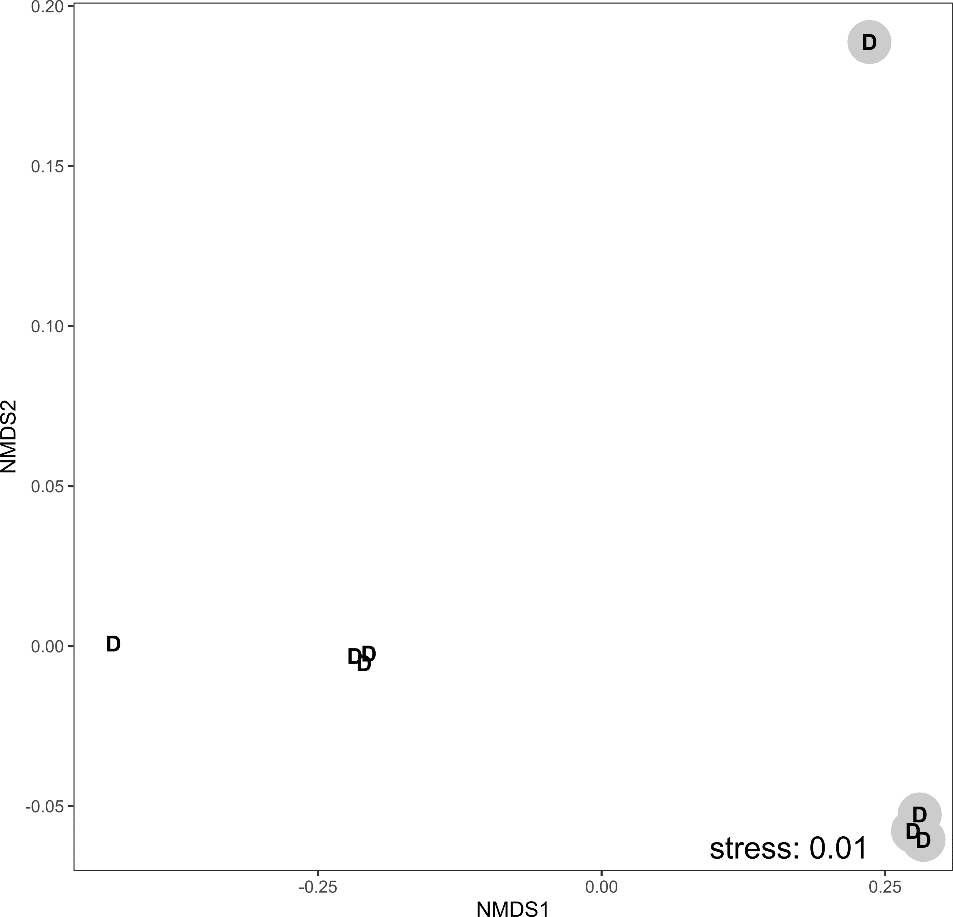


*Lasius niger* complex

*Proatelurina pseudolepisma –* host *Tetramorium*

*Proatelurina pseudolepisma –* host *Lasius*


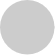


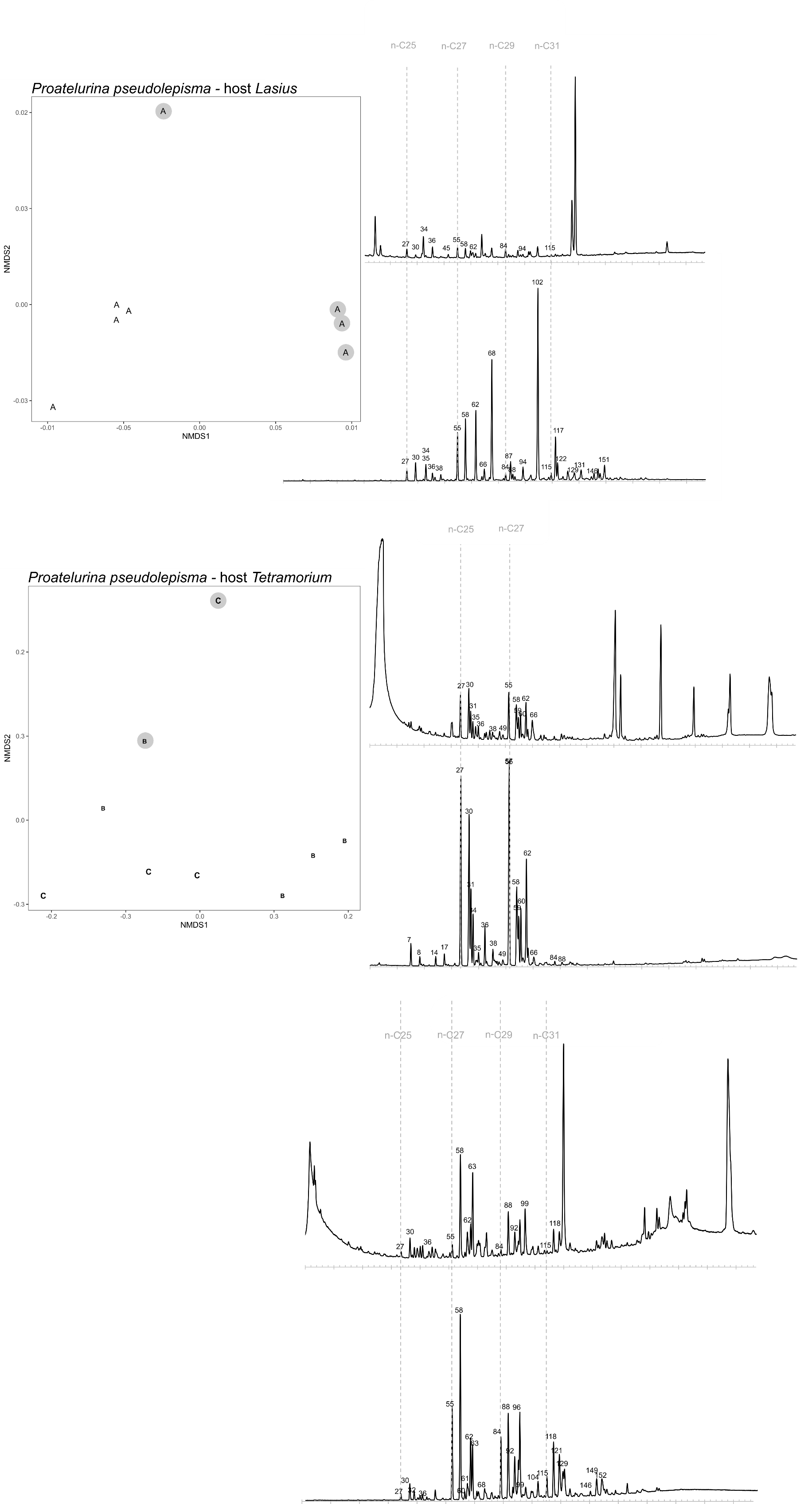


*Proatelurina pseudolepisma*


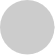


PERMANOVA, Pseudo-F = 7.7, *P* = 0.571 (20 permutations)

PERMDISP, F = 0.3, *P =* 0.608

BC similarity to the ant host ± SD = 0.72 ± 0.14


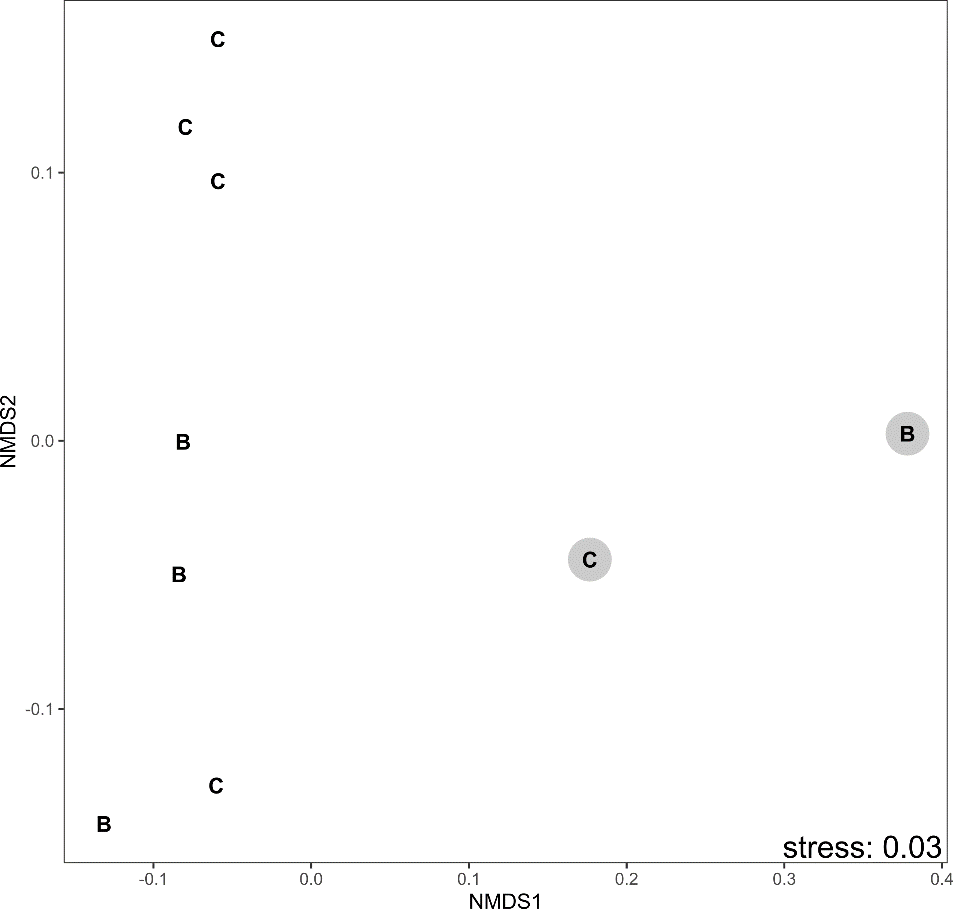


*Tetramorium forte*

*ruginodis*

*Proatelurina pseudolepisma –* host *Tetramorium*

*Proatelurina pseudolepisma*

PERMANOVA, Pseudo-F = 143.4, *P* = 0.042 (70 permutations)

PERMDISP, F = 0.3, *P =* 0.608

BC similarity to the ant host ± SD = 0.33 ± 0.01

**Facultatively associated species**

*Lepisma baetica ­*– host *Tetramorium*


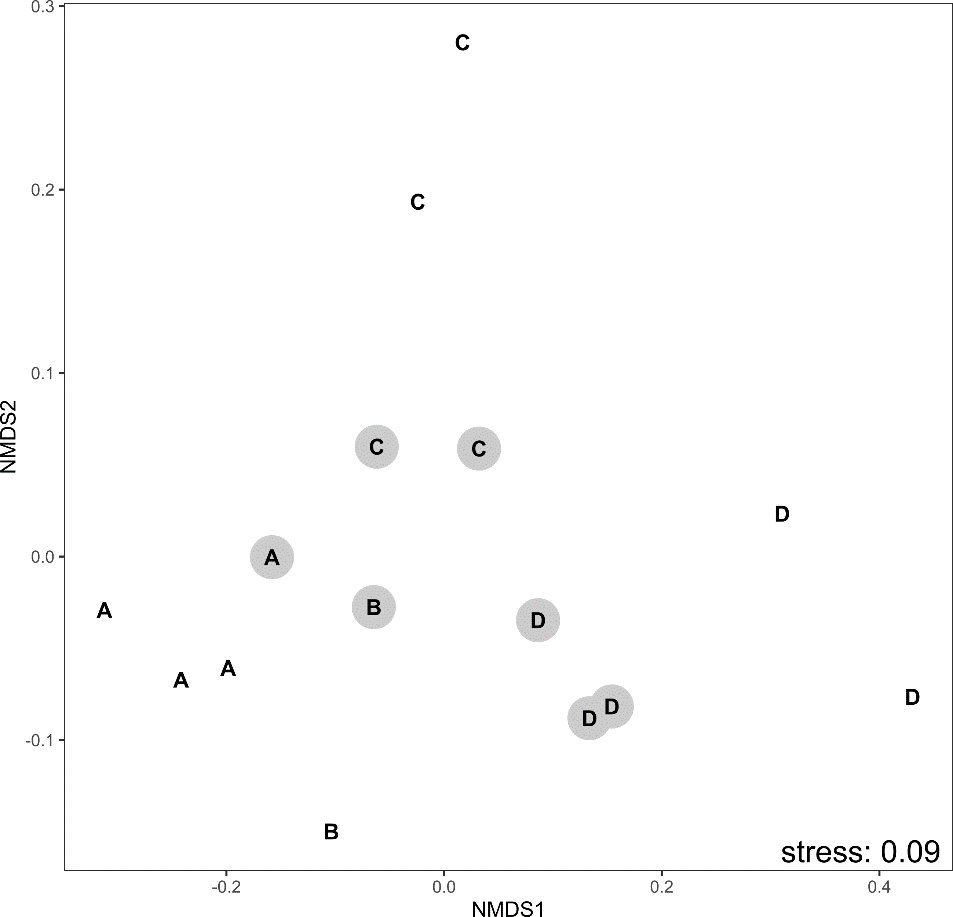

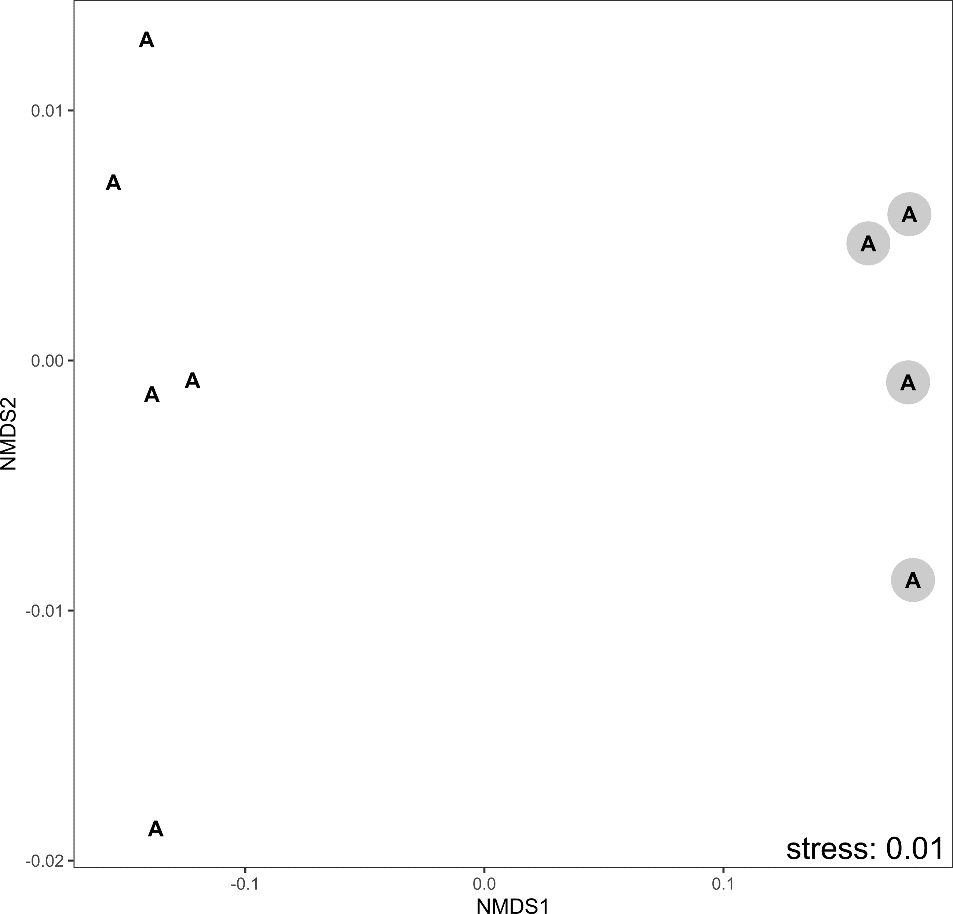
**
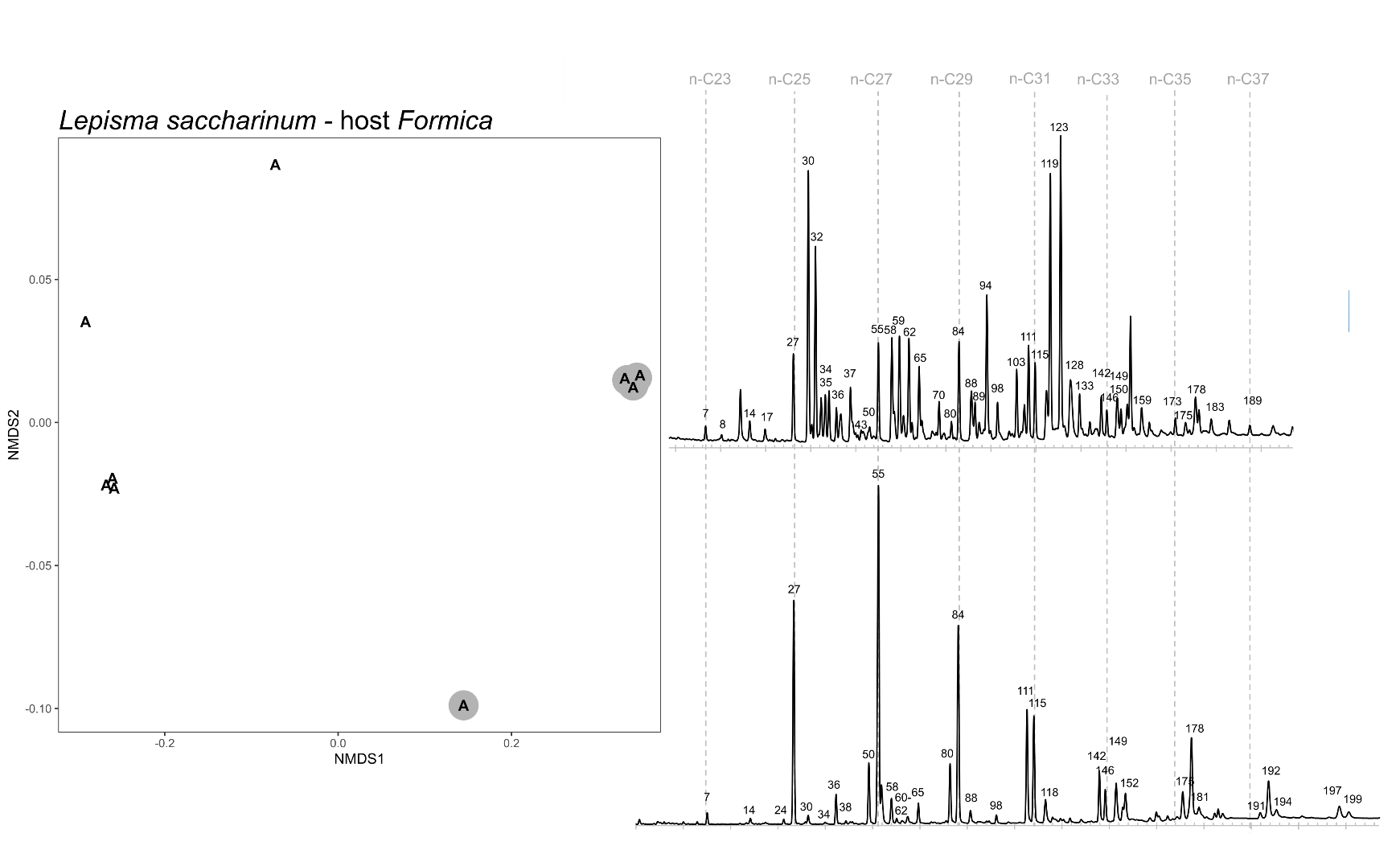
**
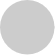
**Unassociated species**

Colony A and B: *Tetramorium forte ruginodis*

Colony C and D: *Tetramorium cf semilaeve*

PERMANOVA, Pseudo-F = 3.1, *P* = 0.025 (120 permutations)

PERMDISP, F = 5.2, *P =* 0.04

BC similarity to the ant host ± SD = 0.57 ± 0.06


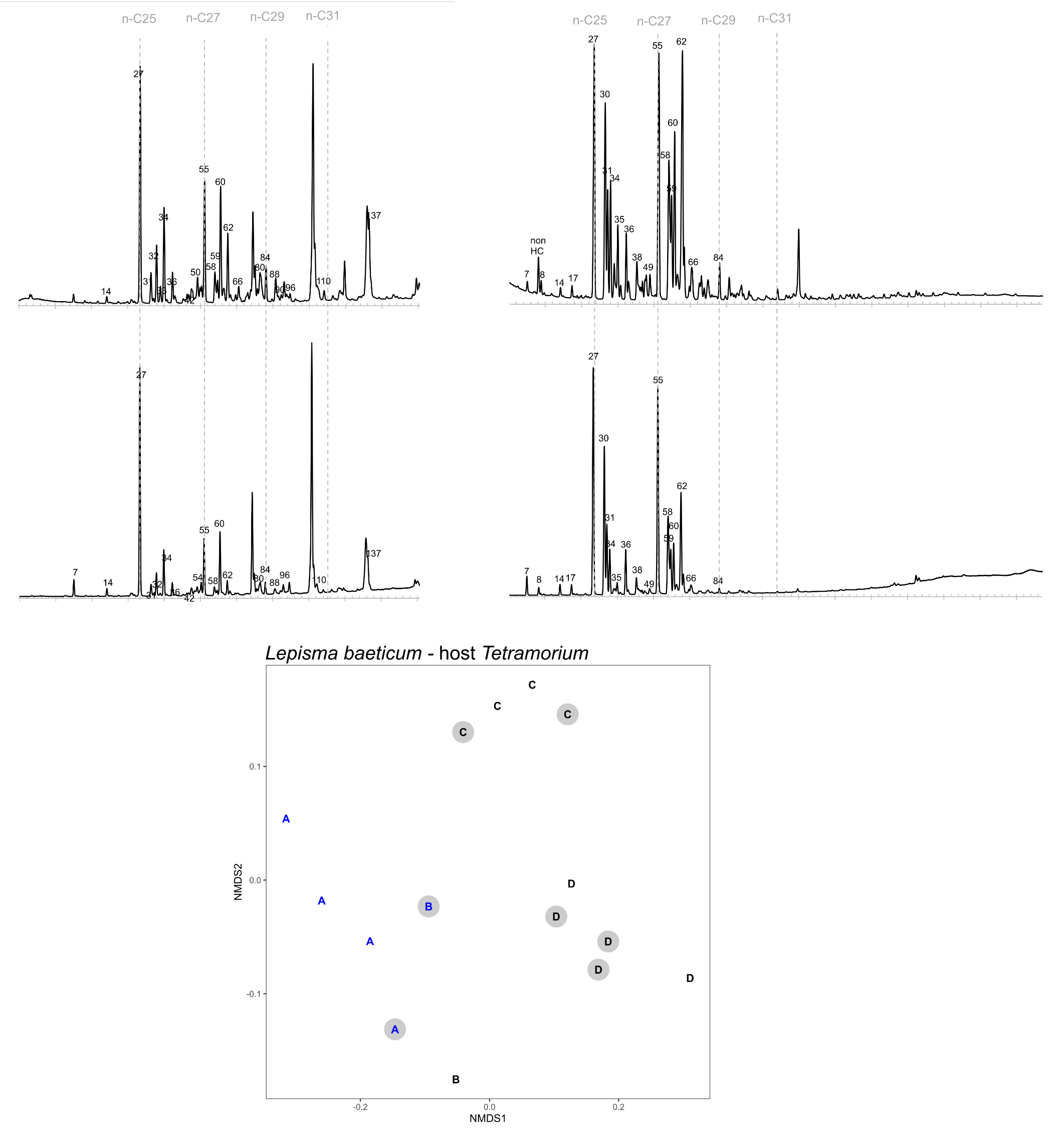


*Lepisma baetica*

*Lepisma baetica*

*Tetramorium semilaeve* cf

*Tetramorium forte ruginodis*


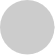

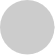


PERMANOVA, Pseudo-F = 59.9, *P* = 0.016 (126 permutations)

PERMDISP, F = 0.1, *P =* 0.766

BC similarity to the ant host ± SD = 0.25 ± 0.04

*Lepisma saccharinum*

*Formica rufa*

*Lepisma saccharinum* – host *Formica*


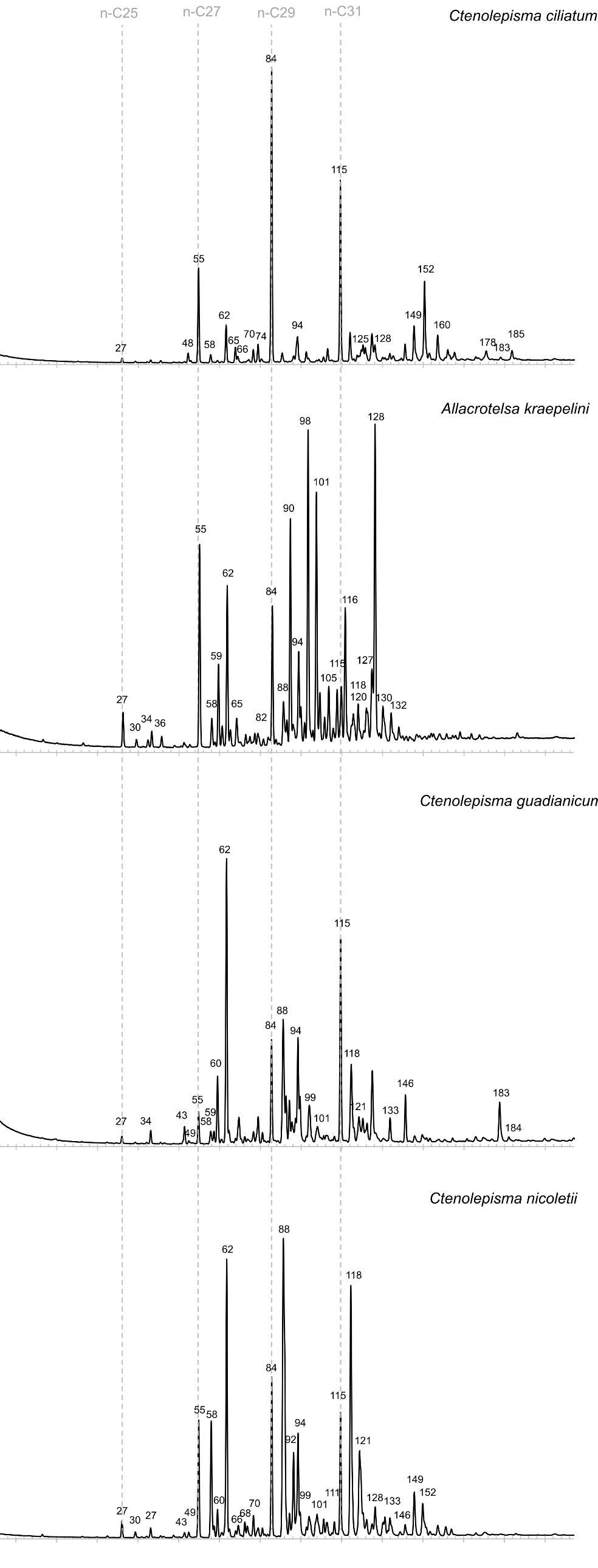

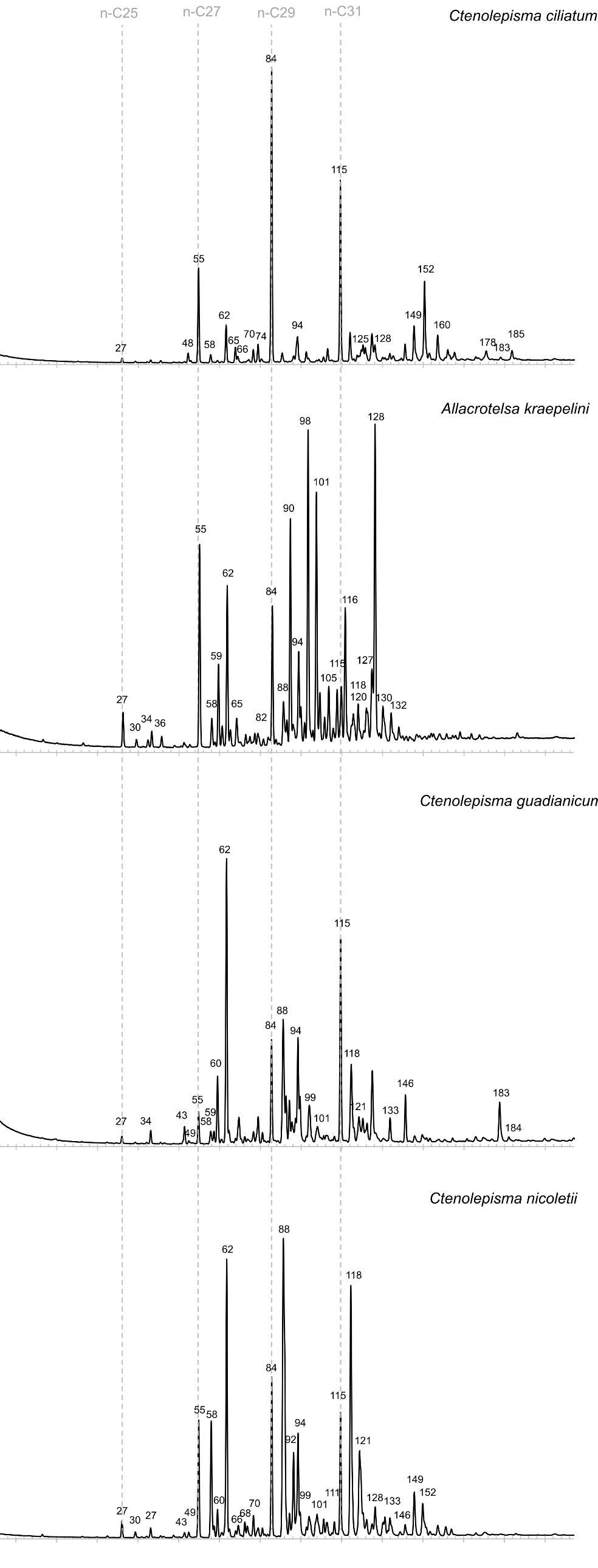


n-C31
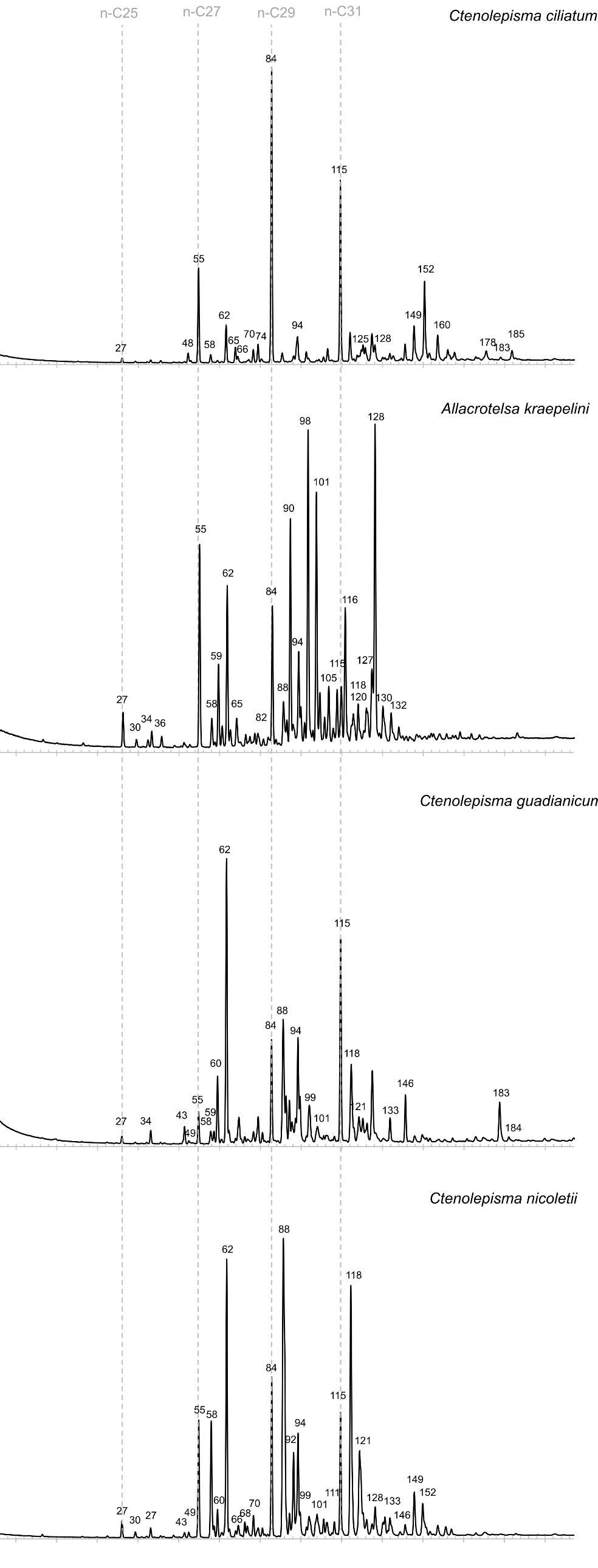

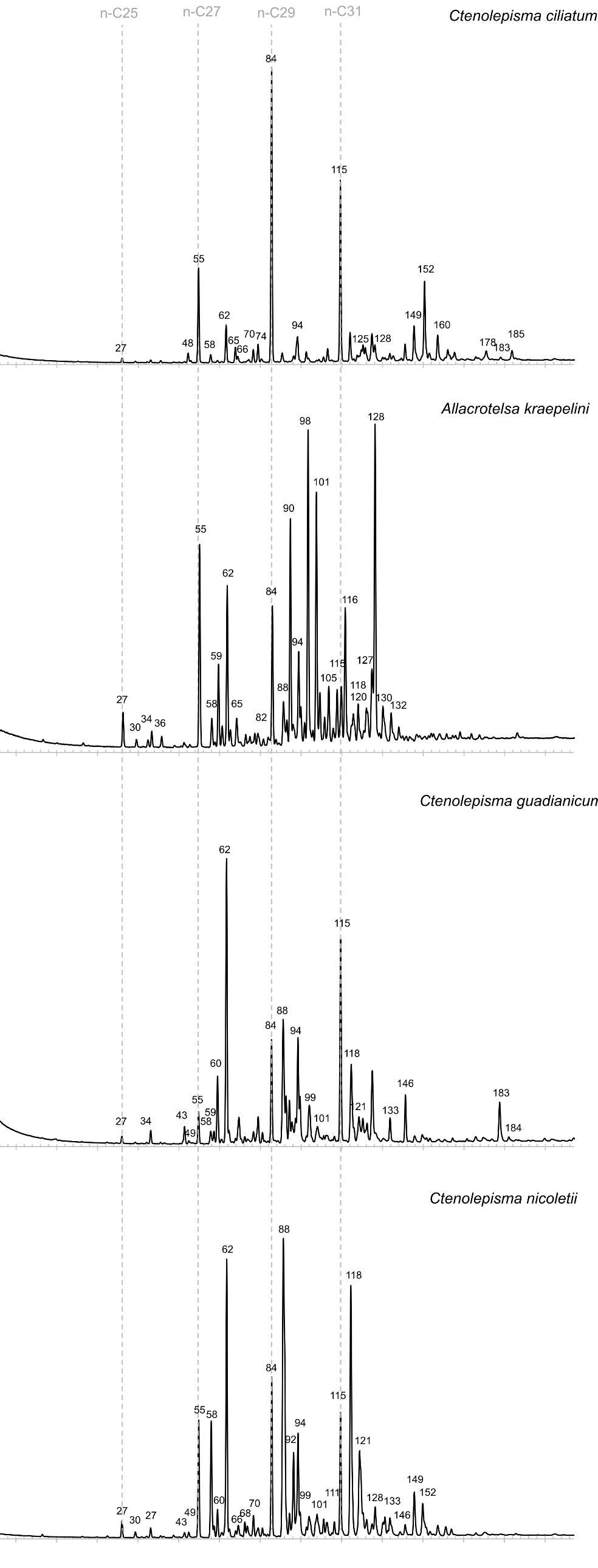
7
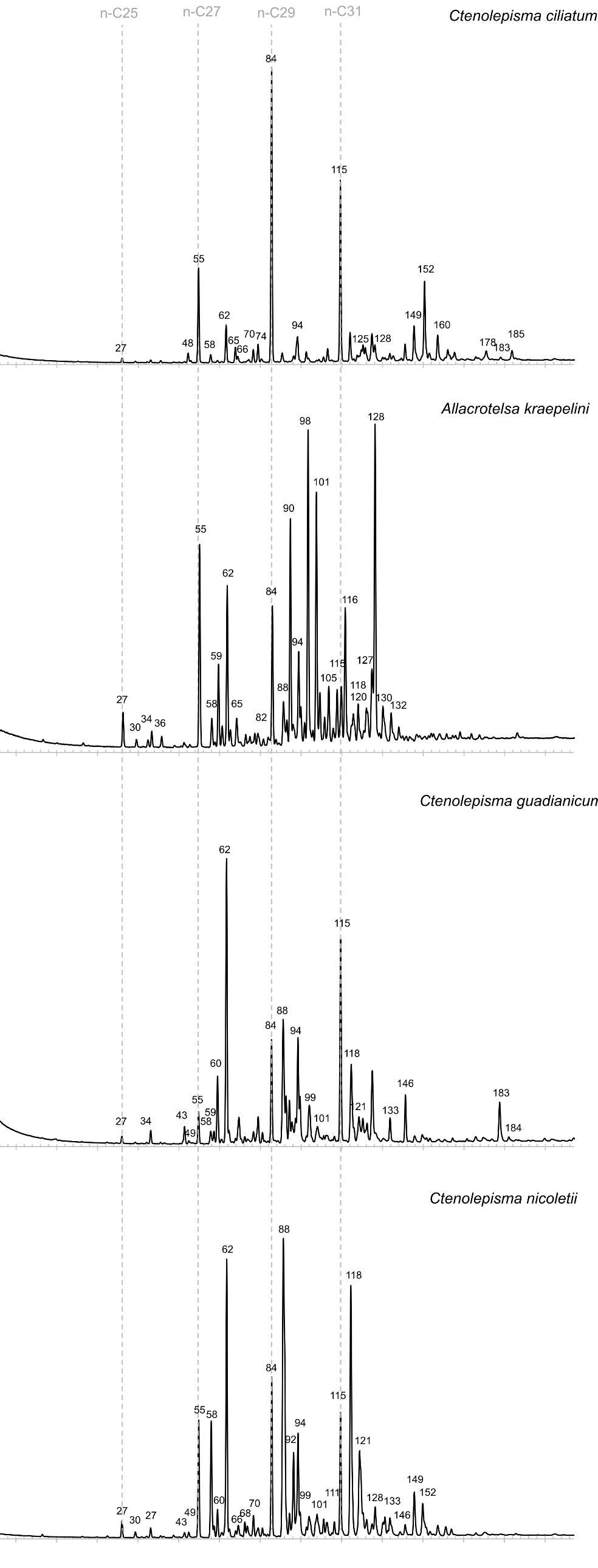
5

n-C25C29
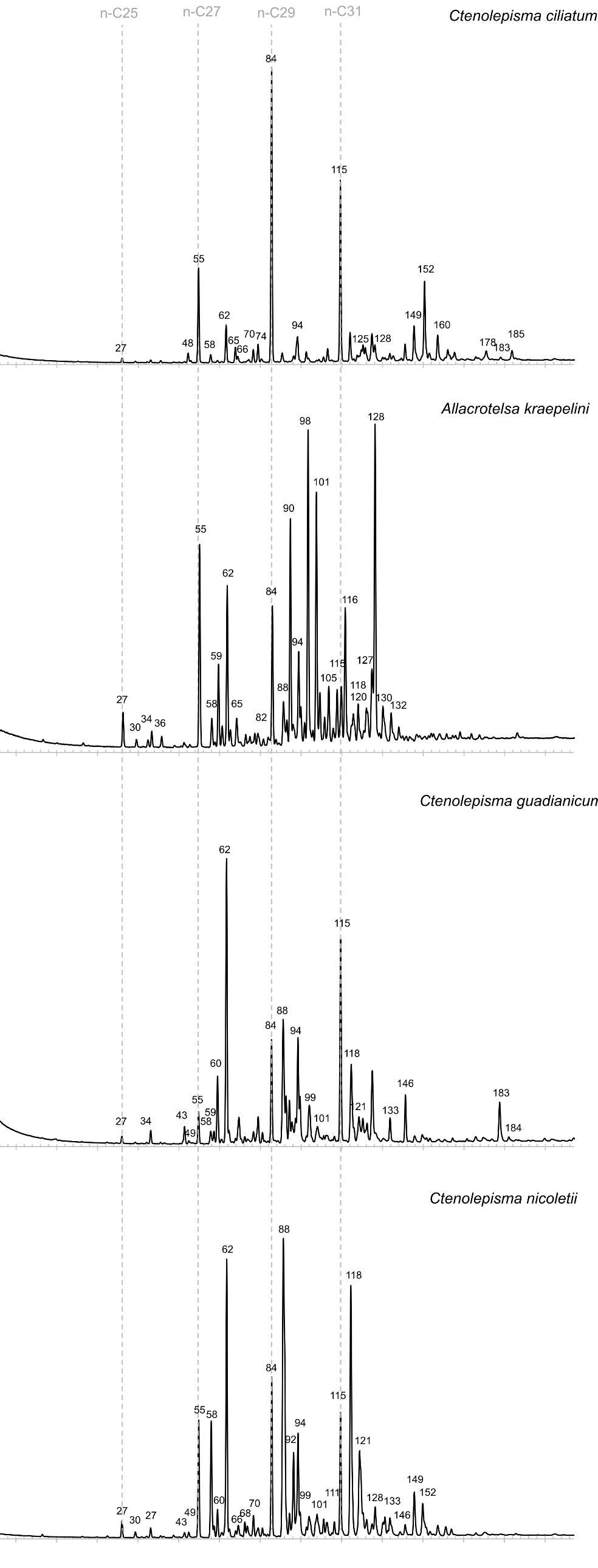

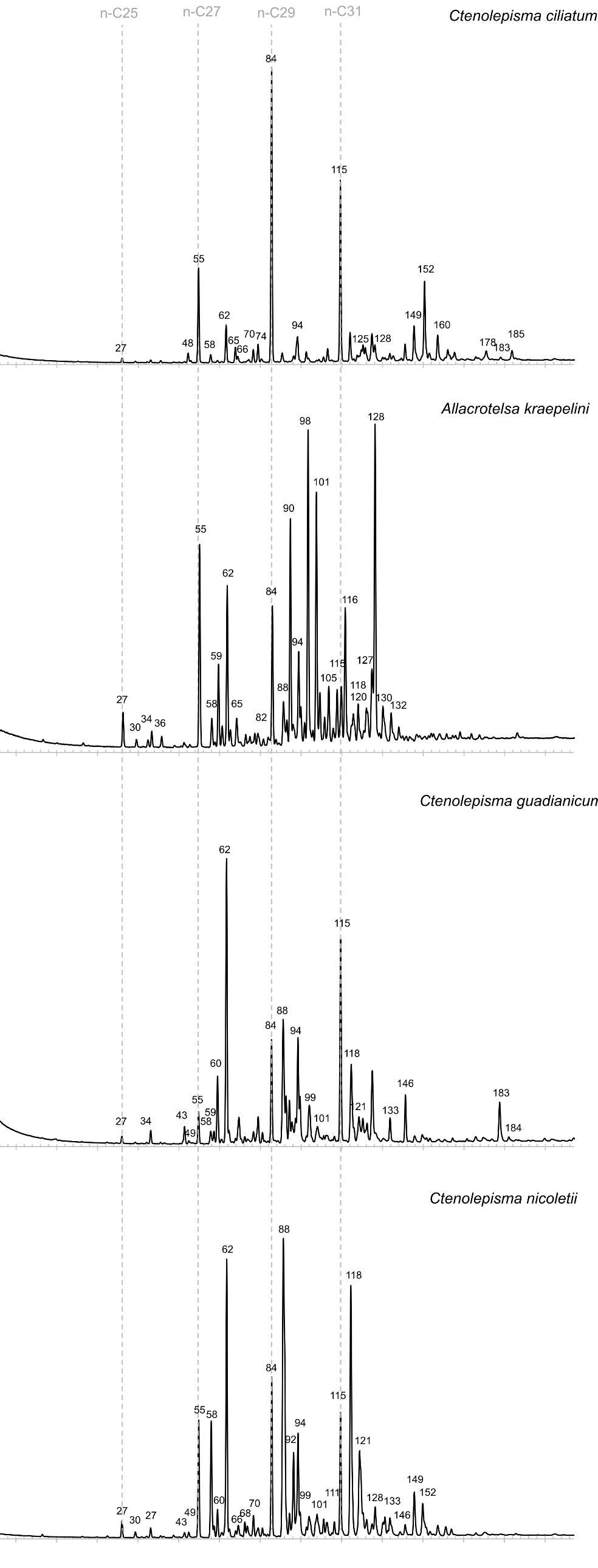
7
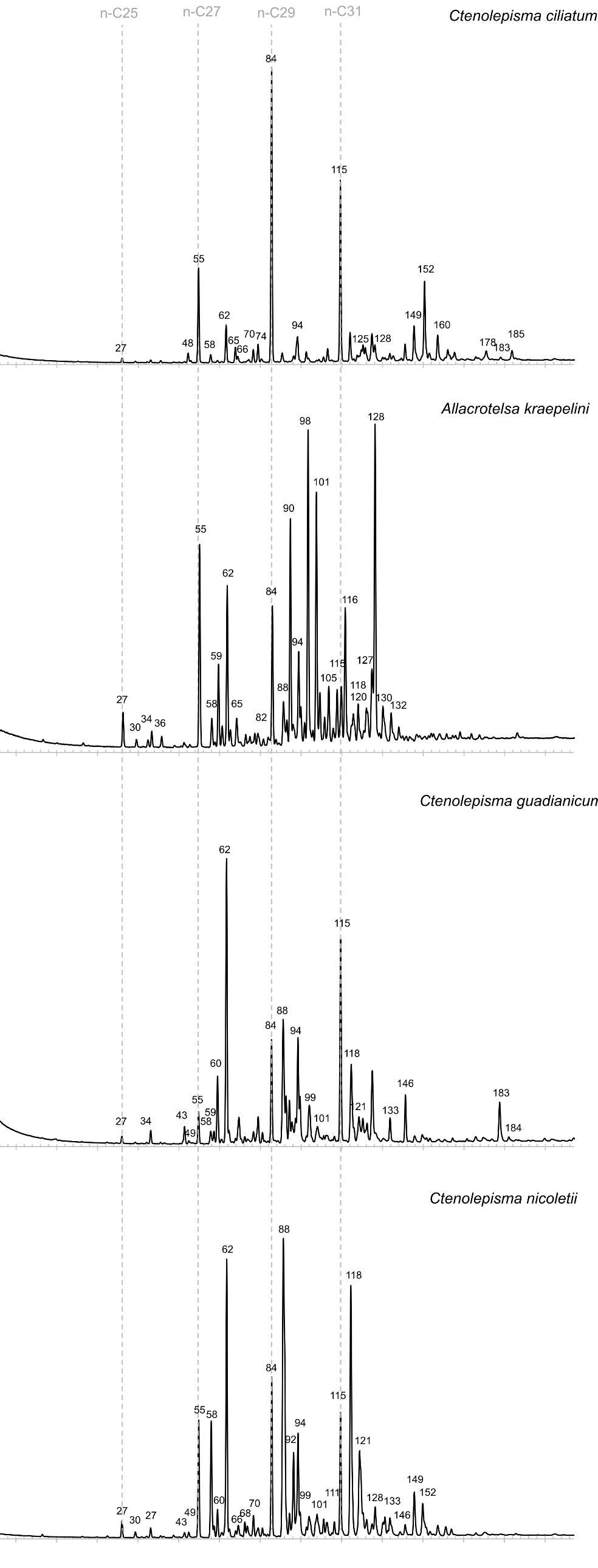
5

n-C29
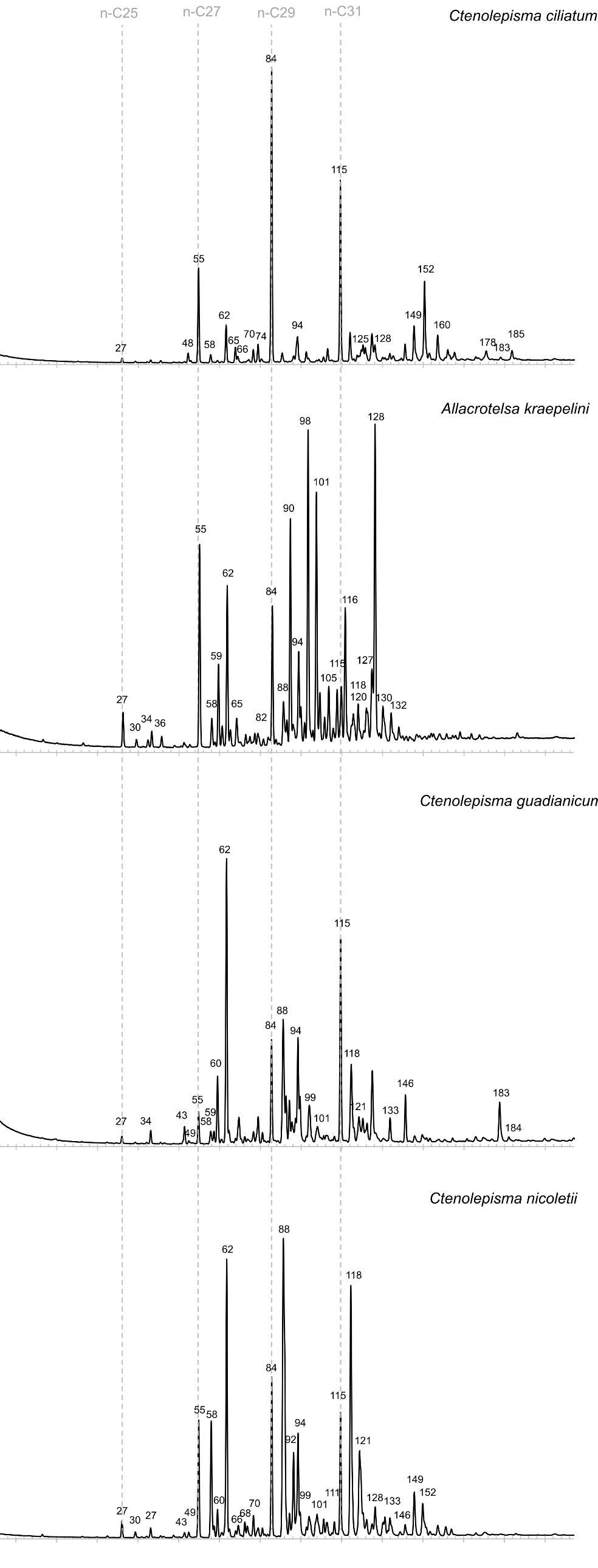

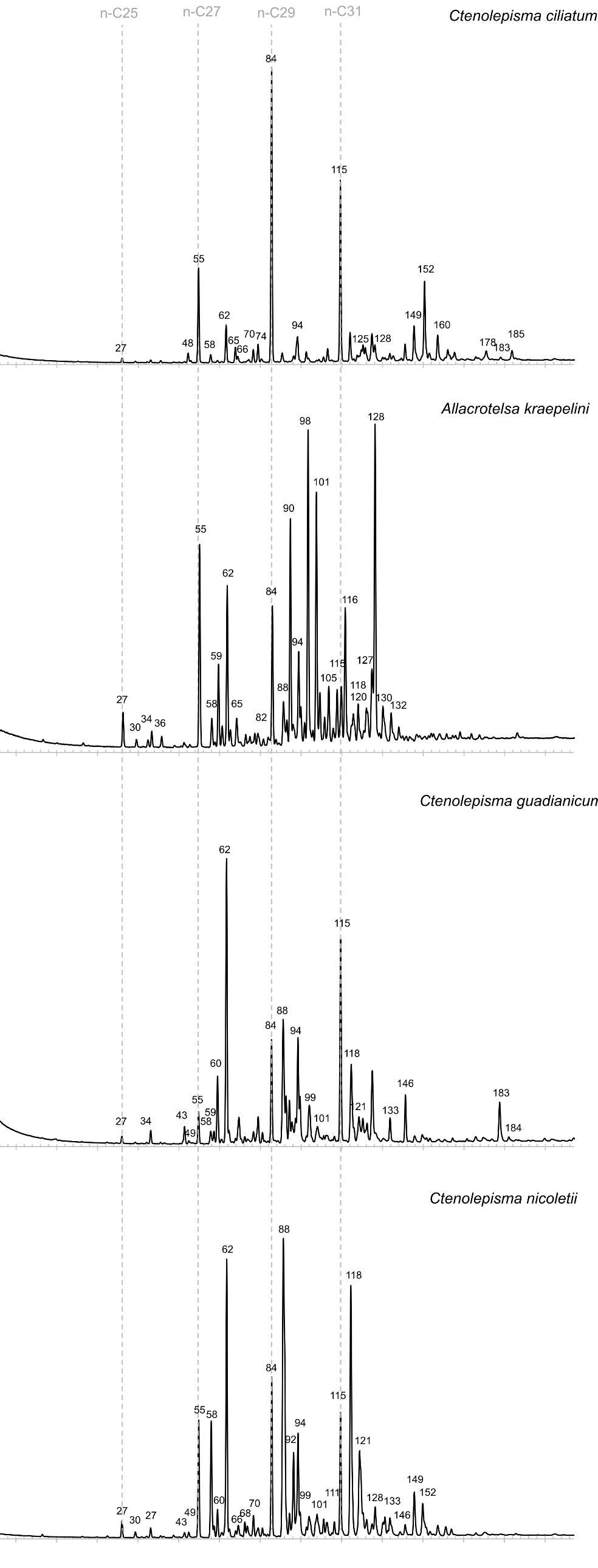
7
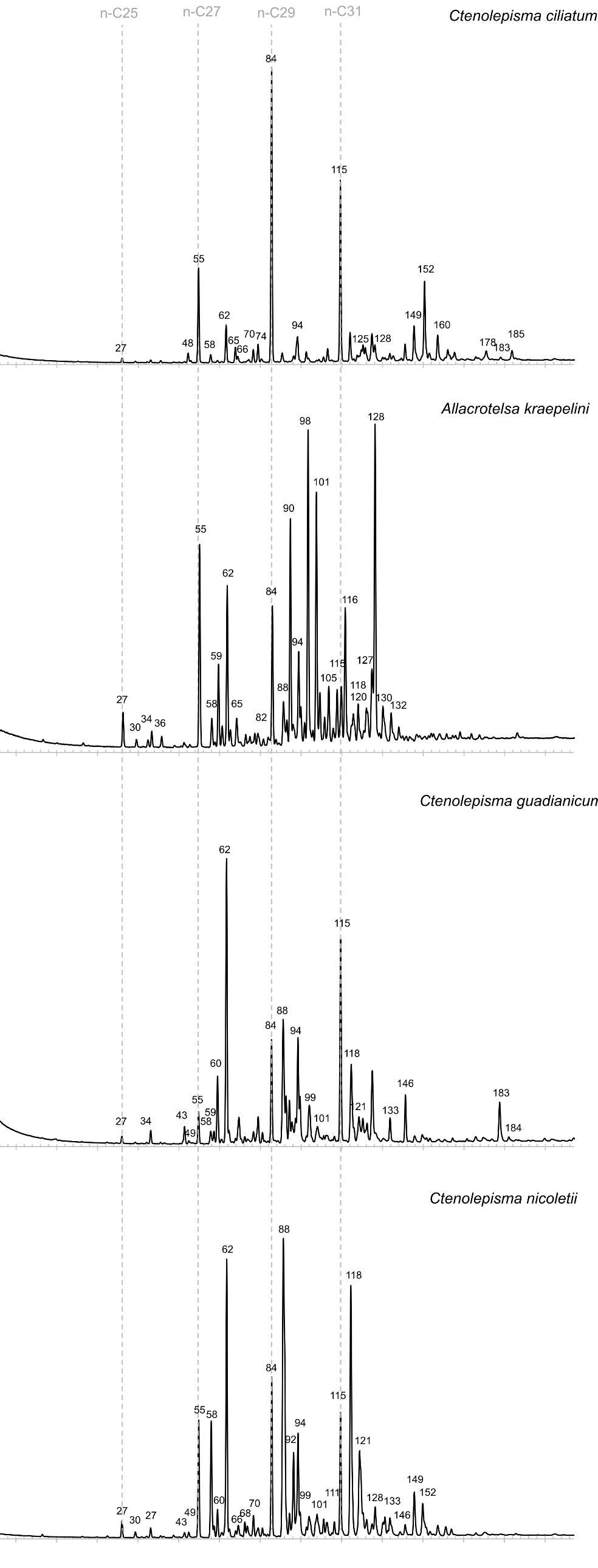
5

n-C27C29
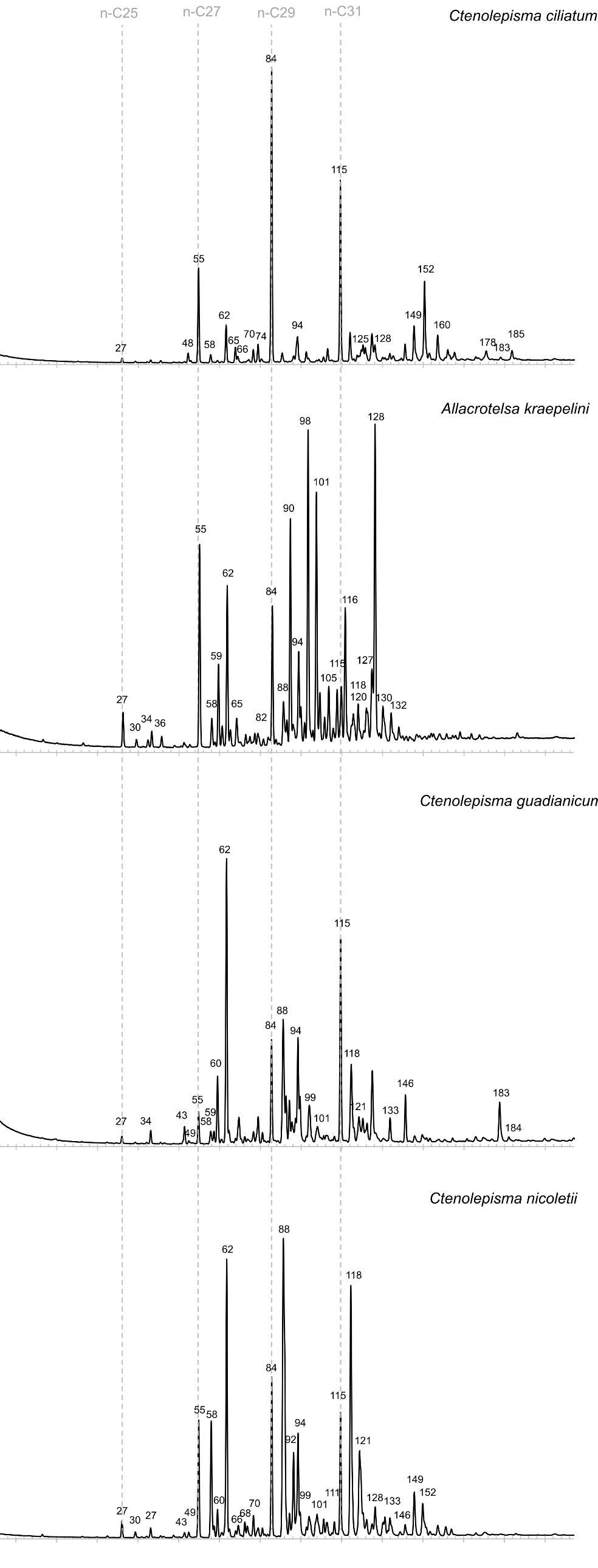

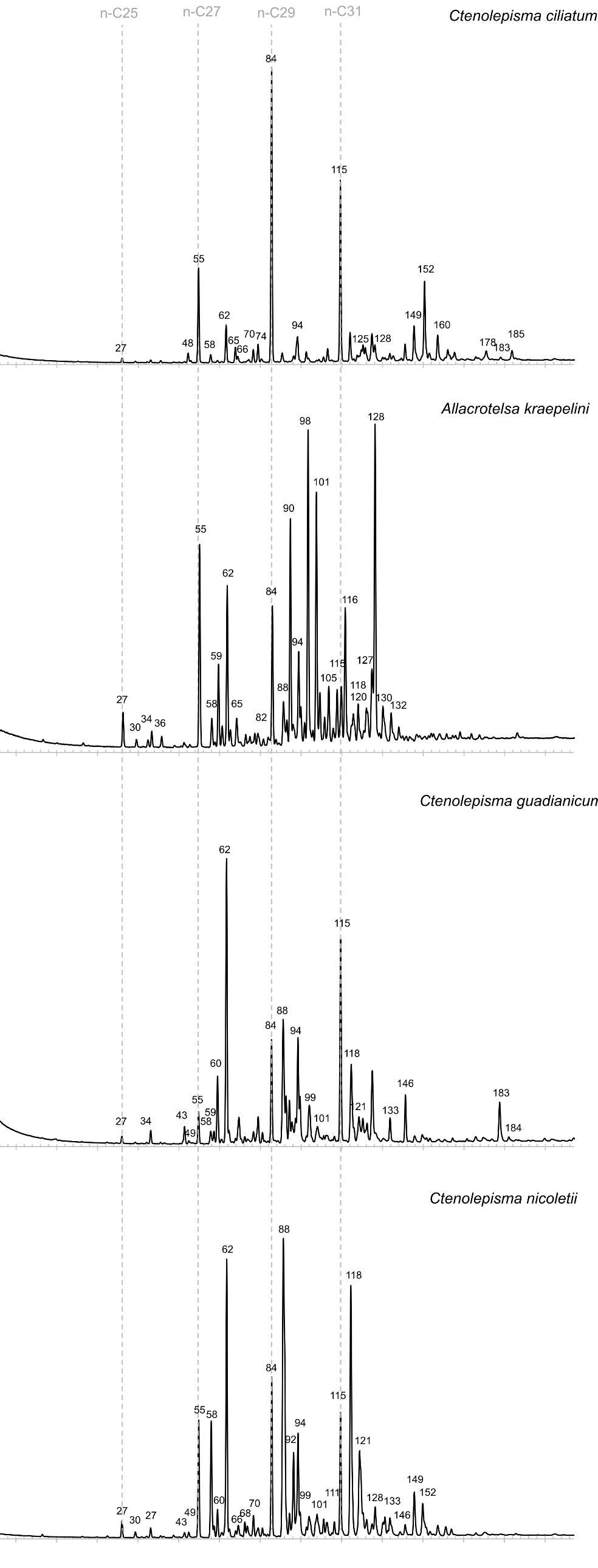
7
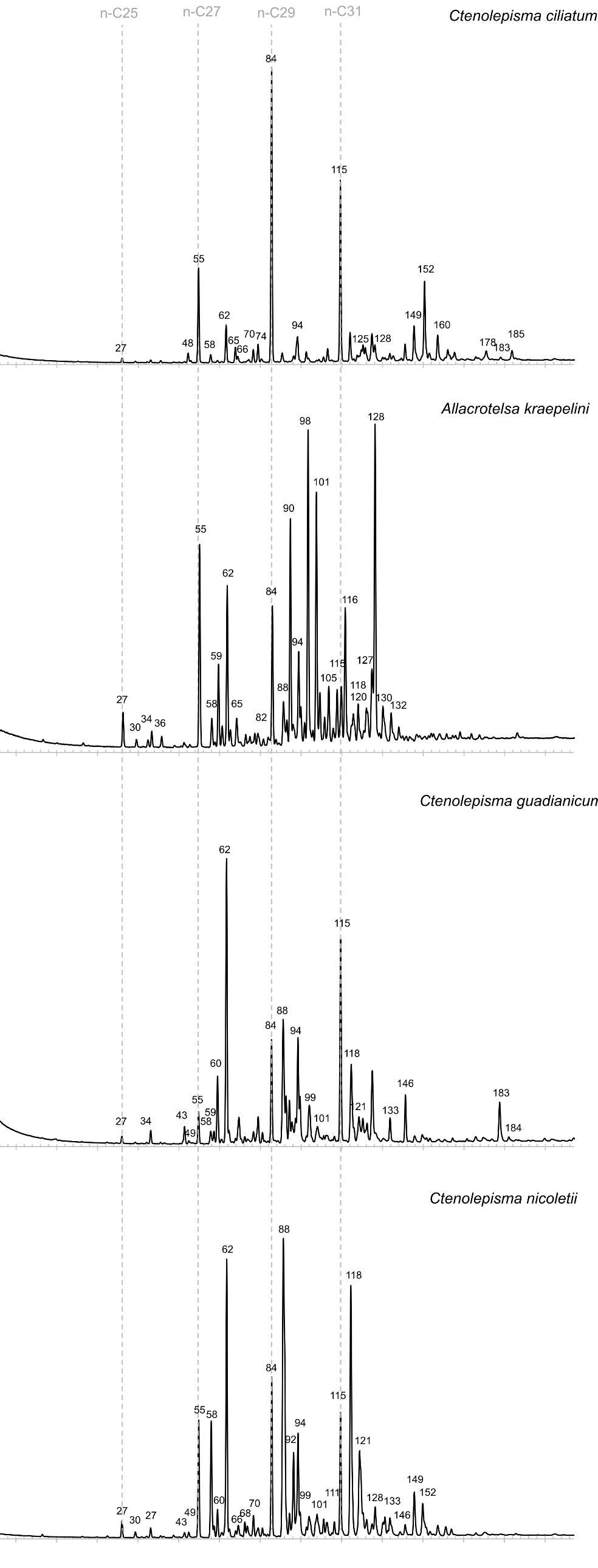
5

n-C25C29
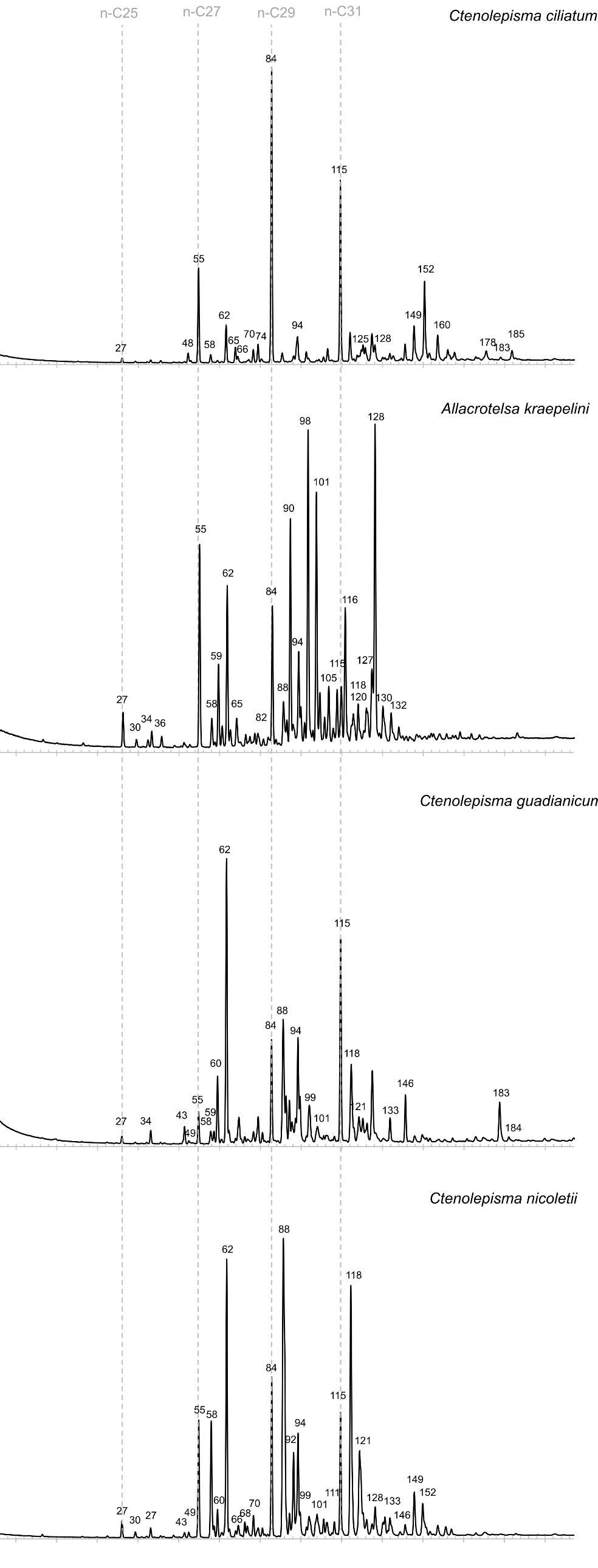

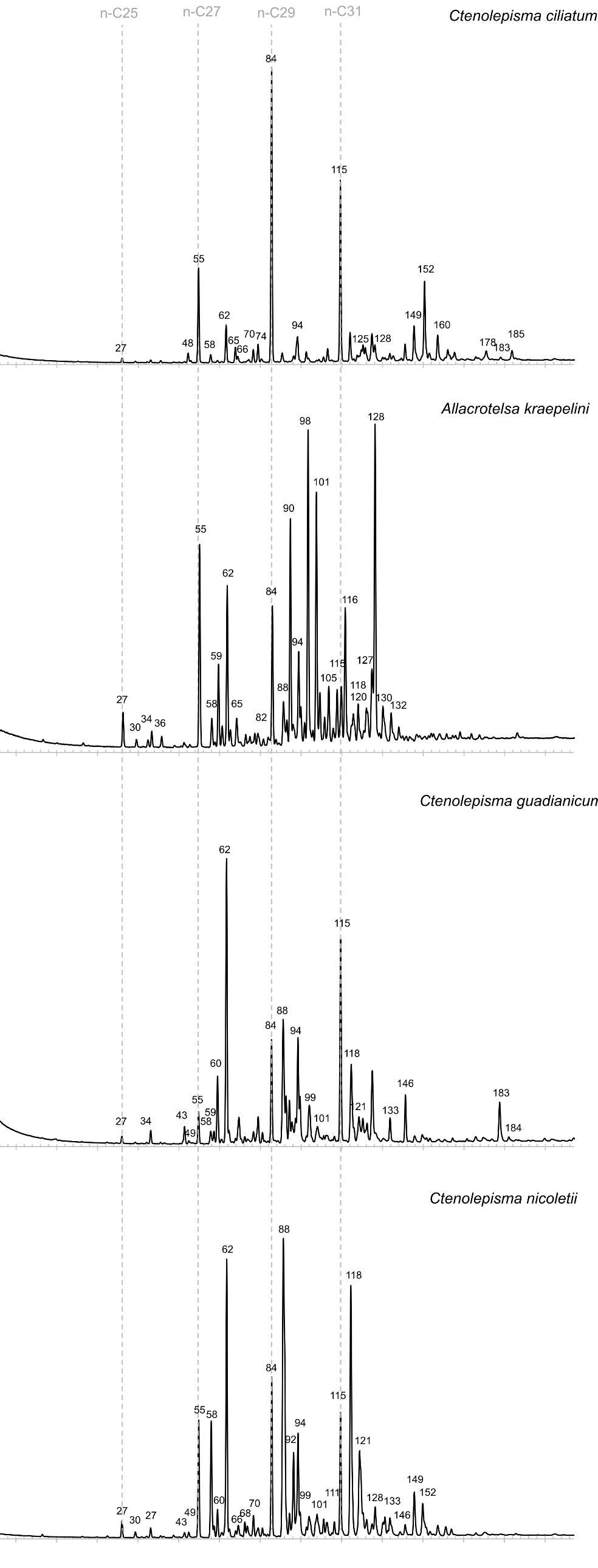
7
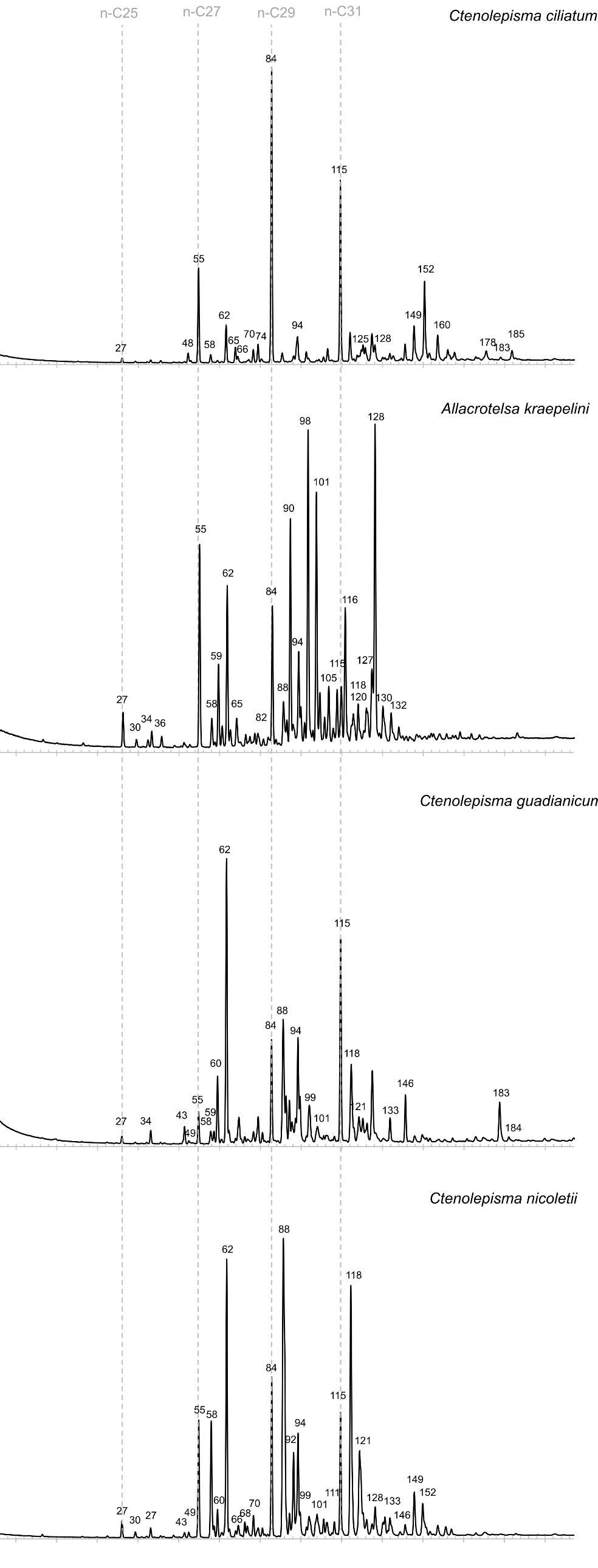
5

n-C29
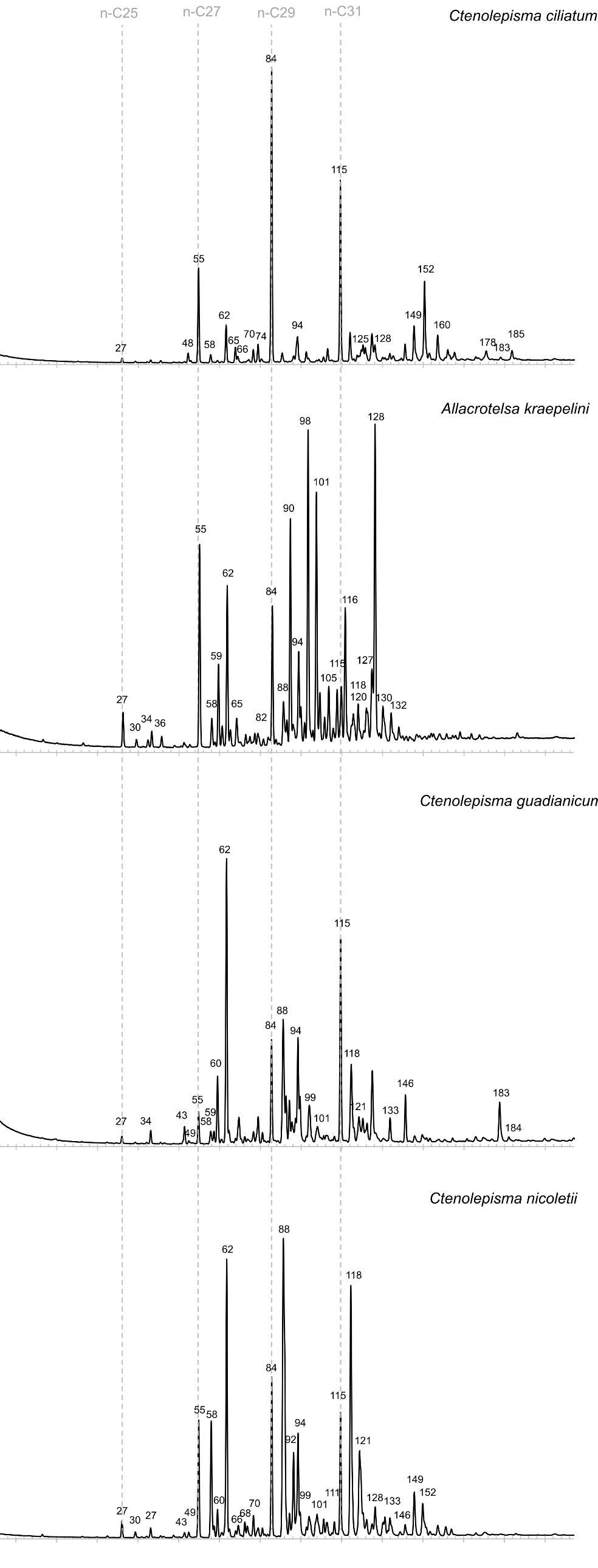

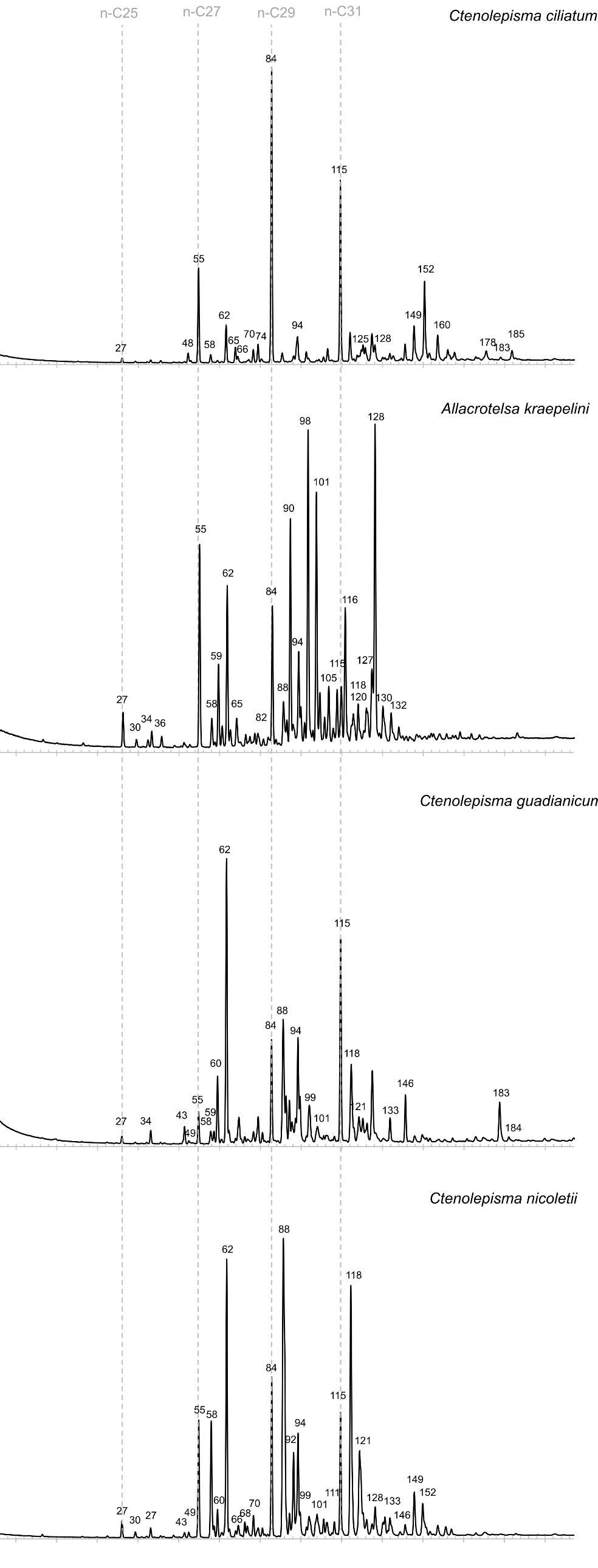
7
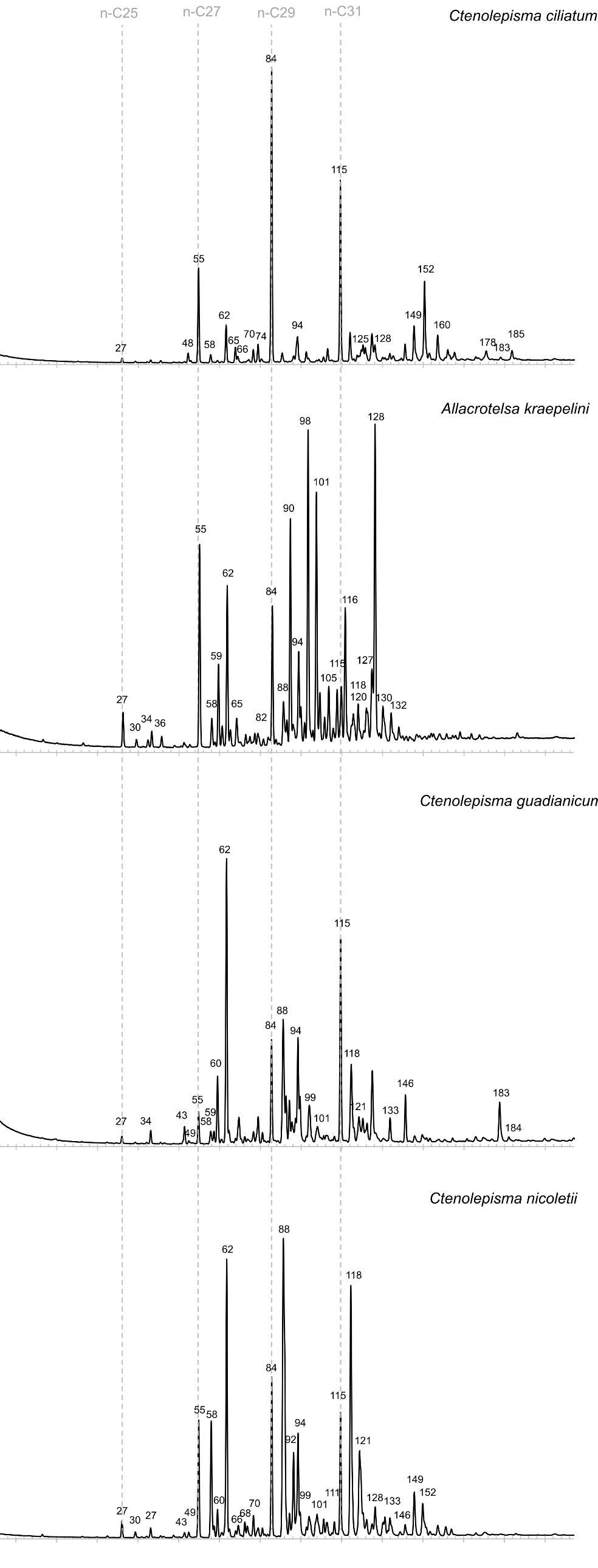
5

n-C31
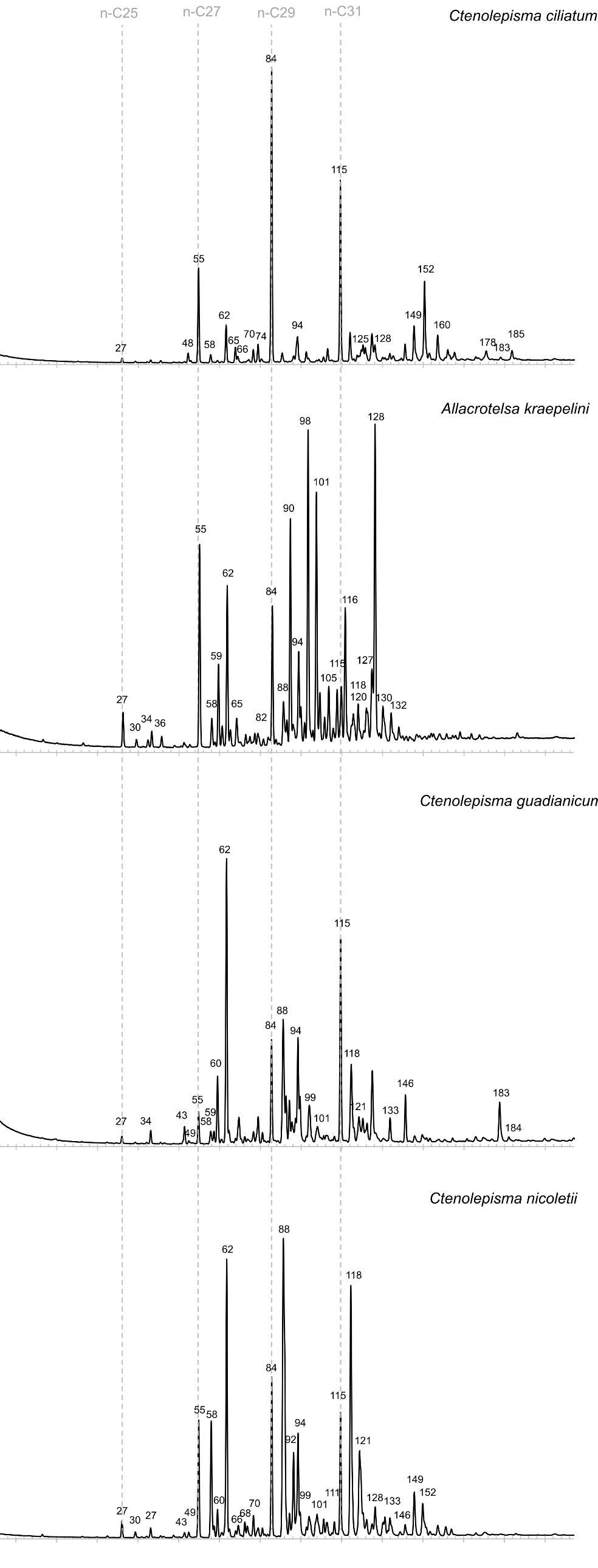

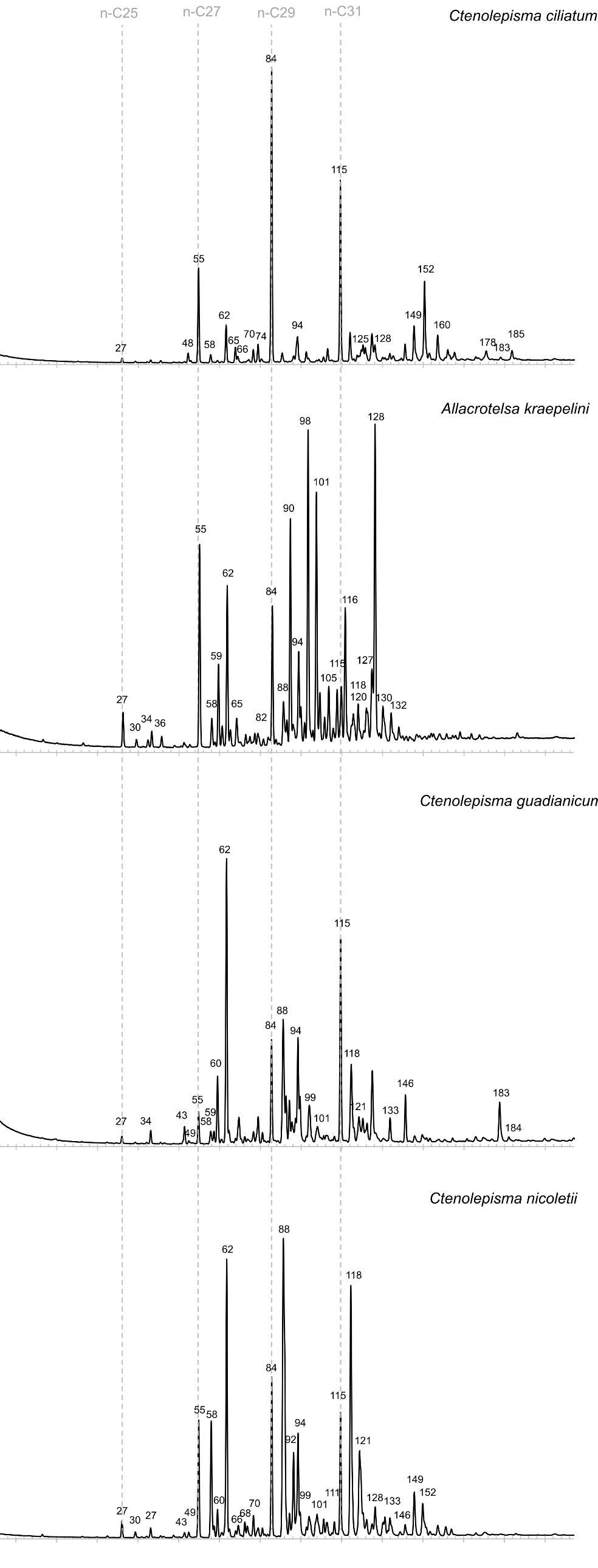
7
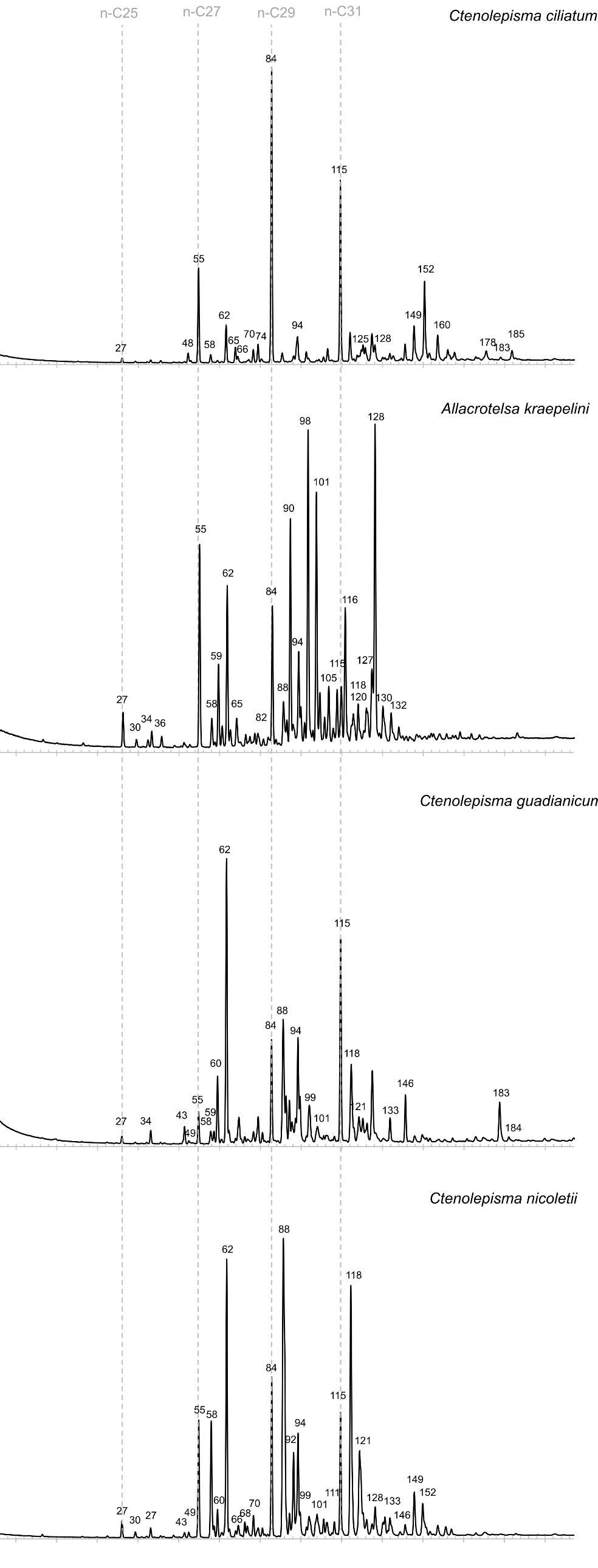
5

n-C27C29
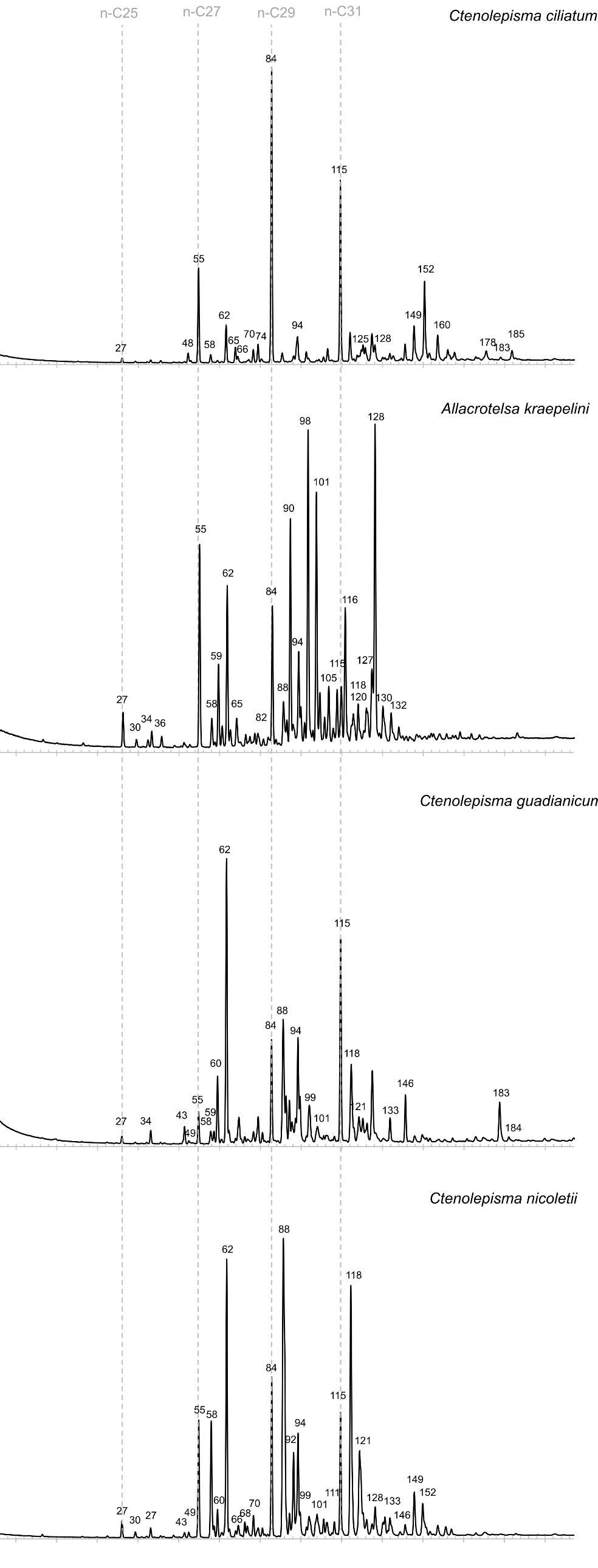

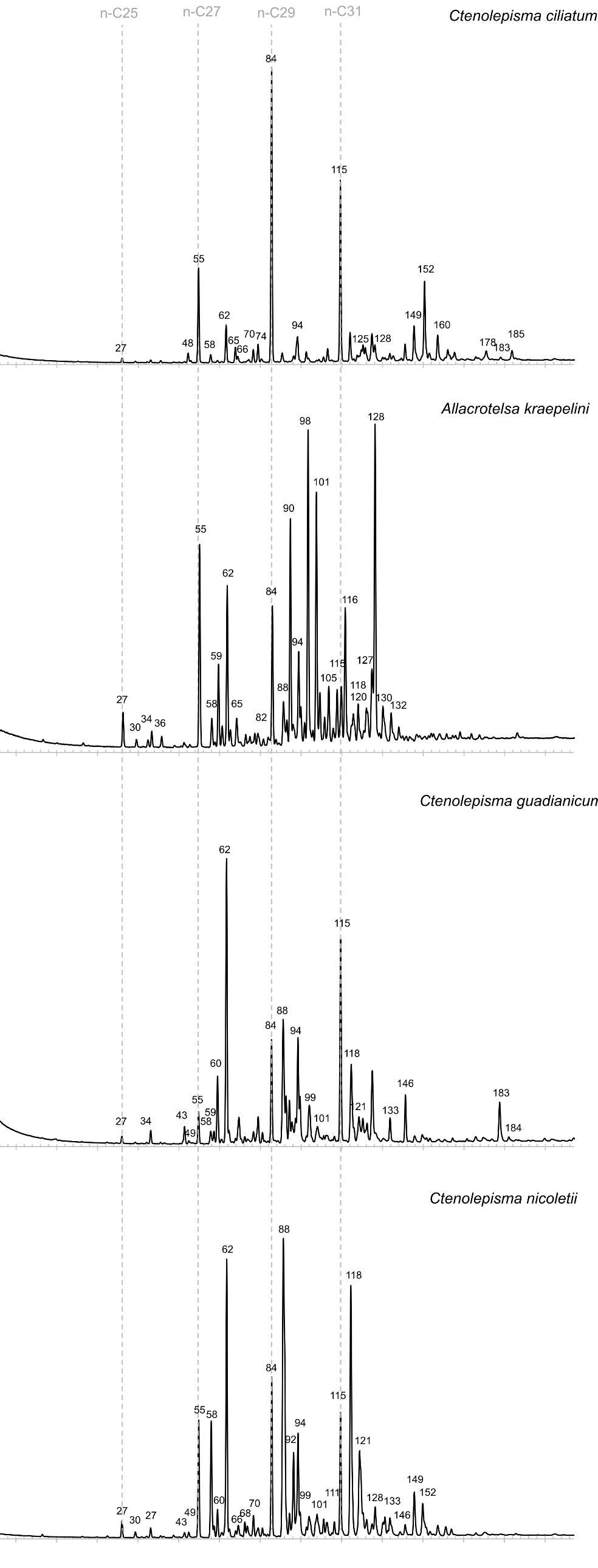
7
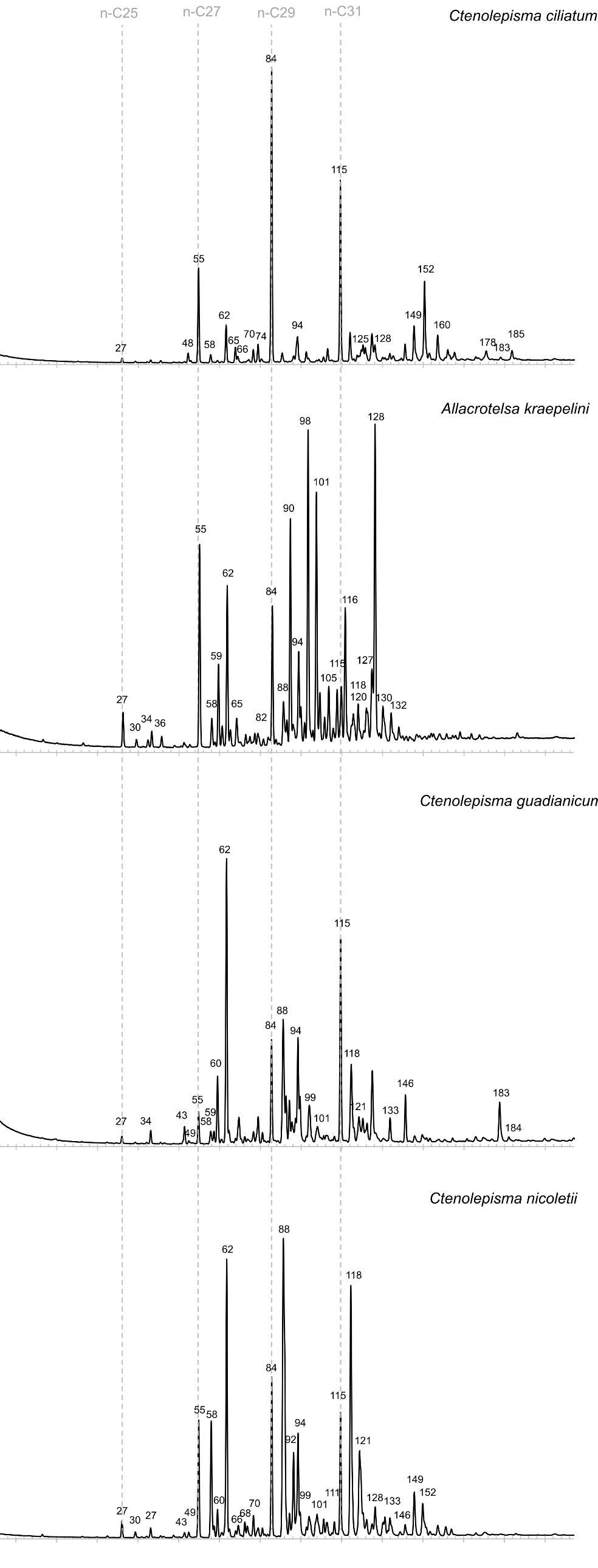
5

*Ctenolepisma ciliatum*

*Allacrotelsa kraepelini*


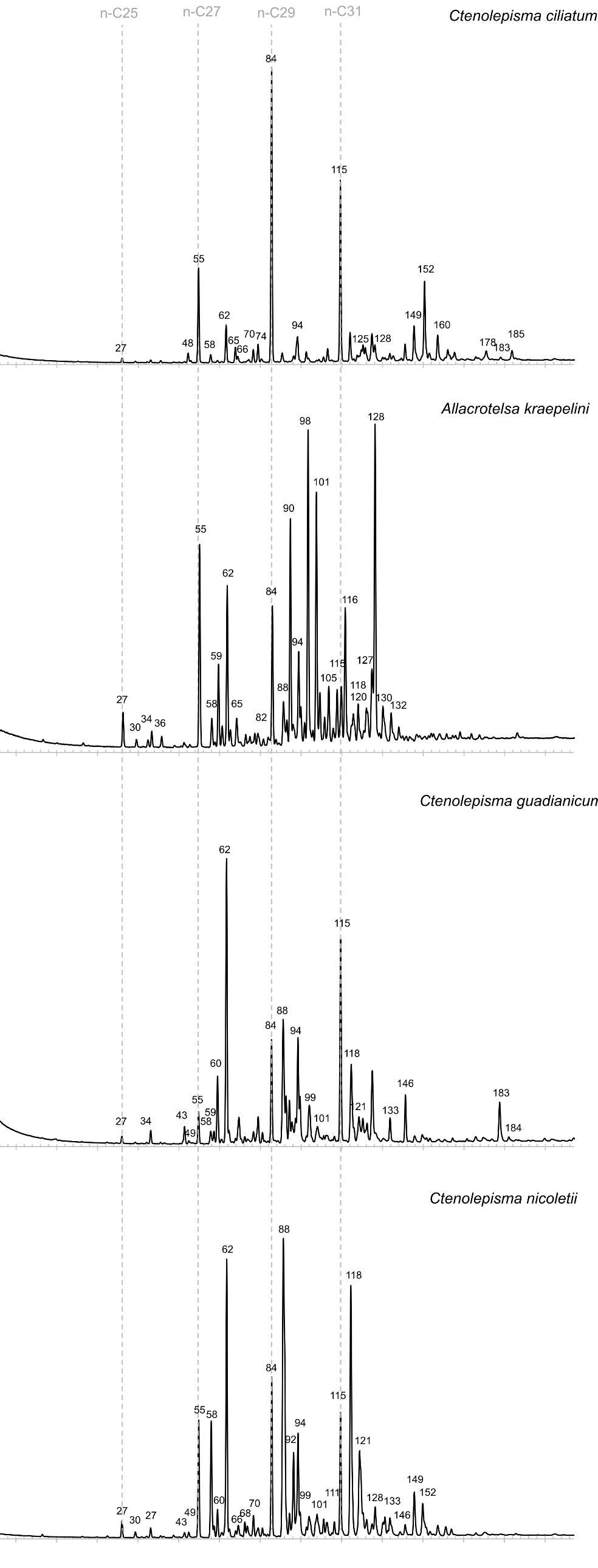

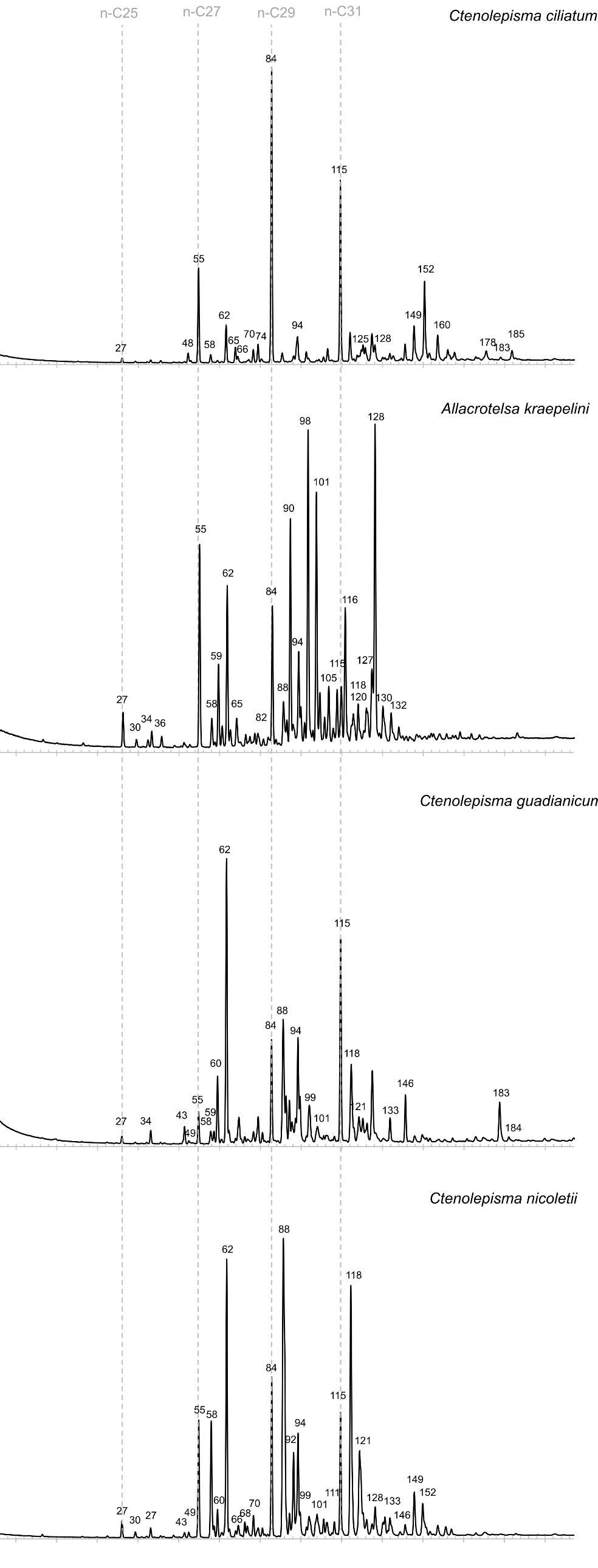


n-C25C29
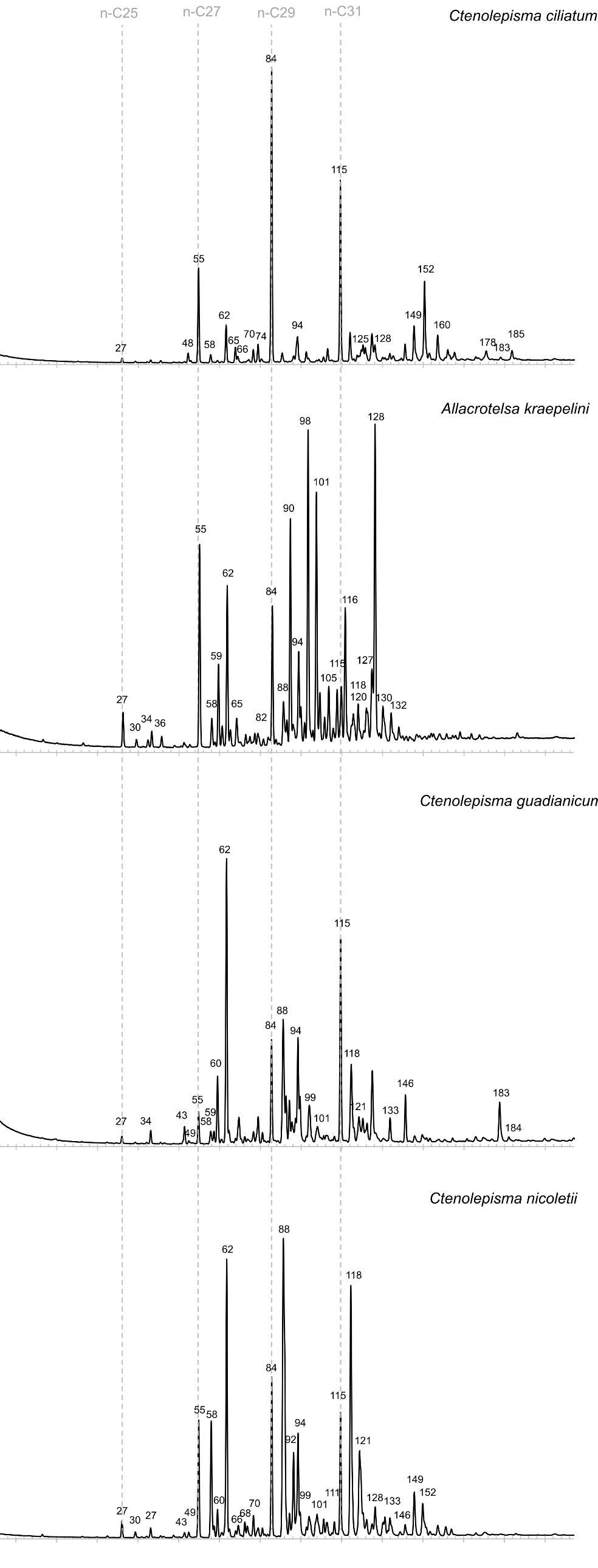

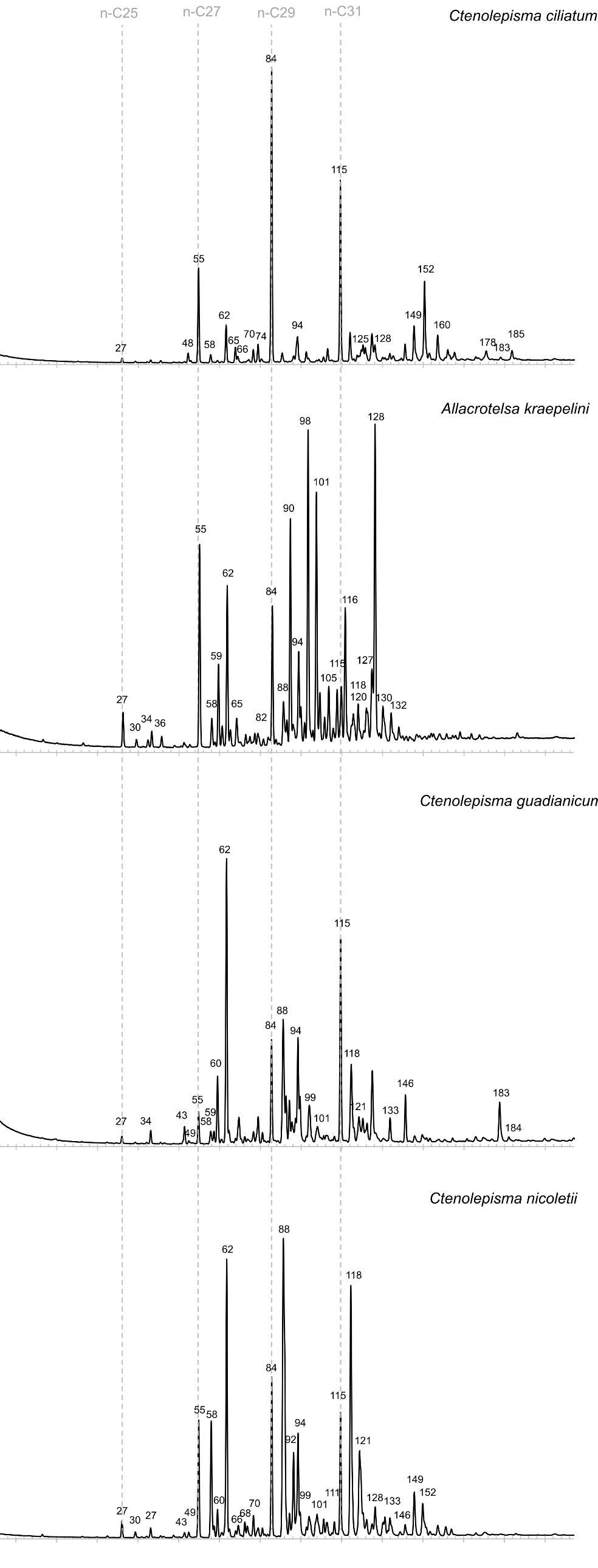
7
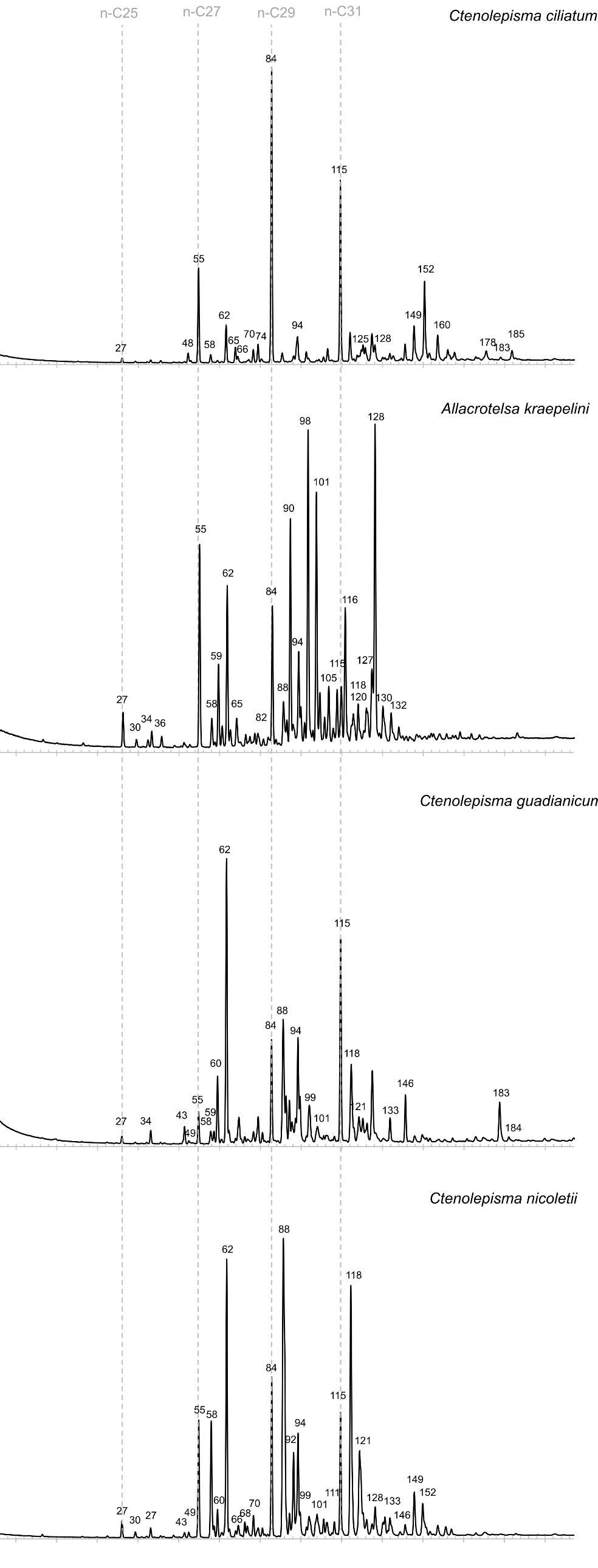
5

n-C29
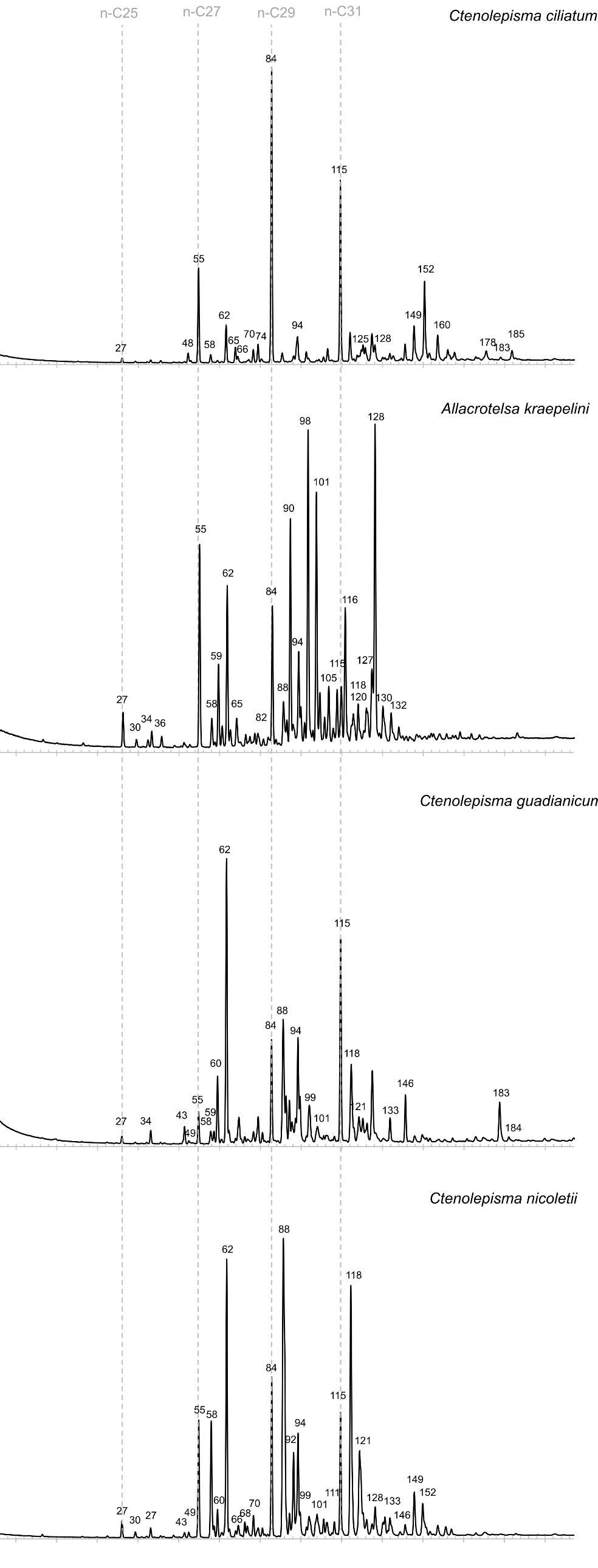

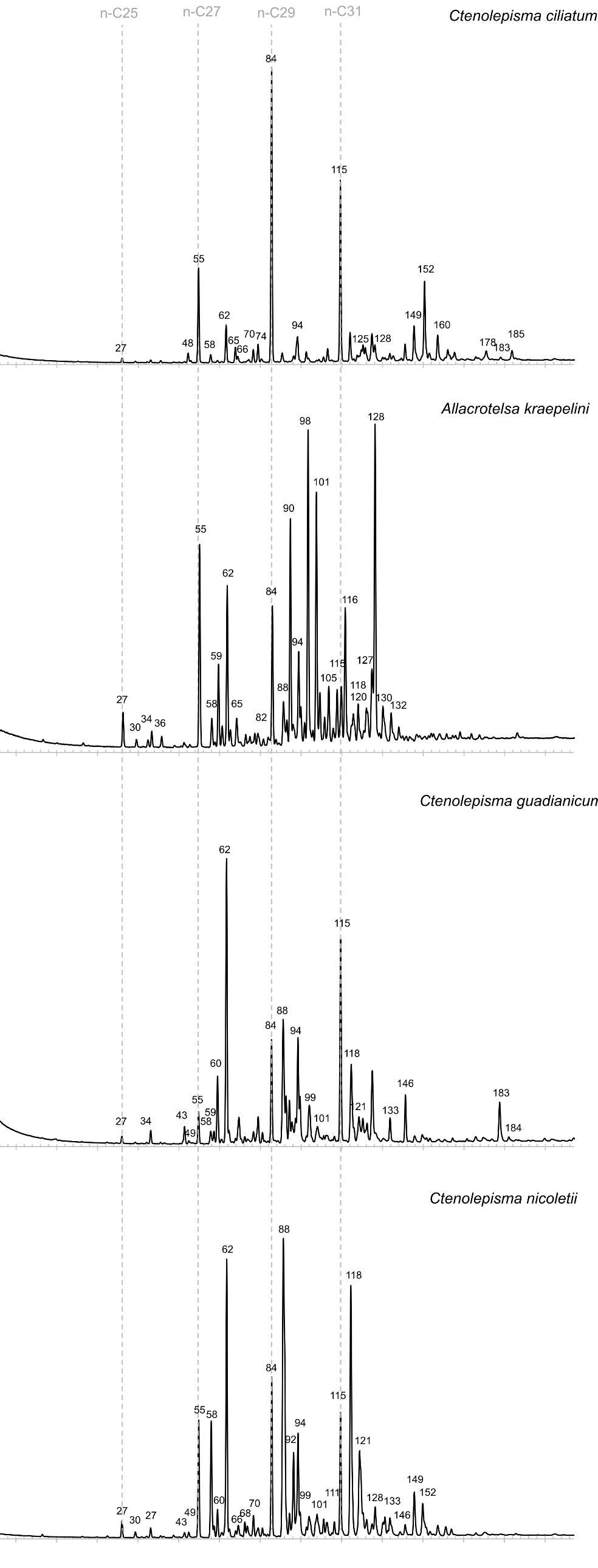
7
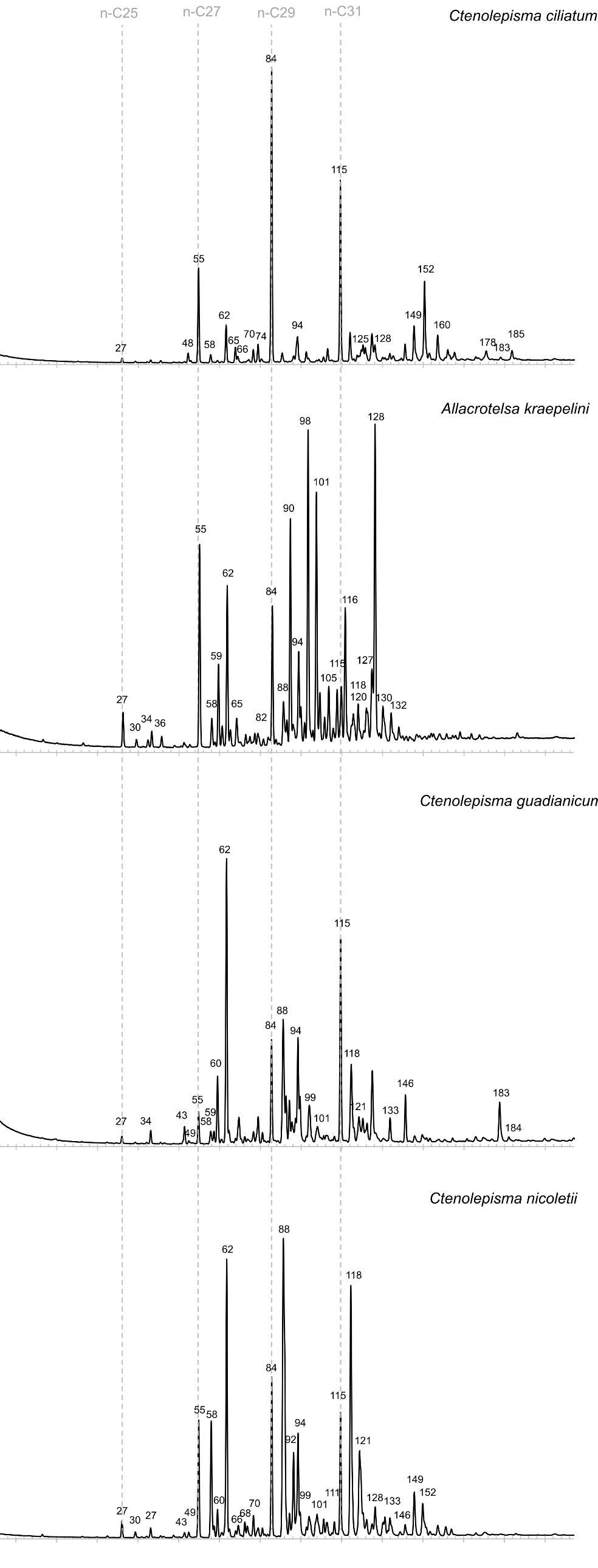
5

n-C31
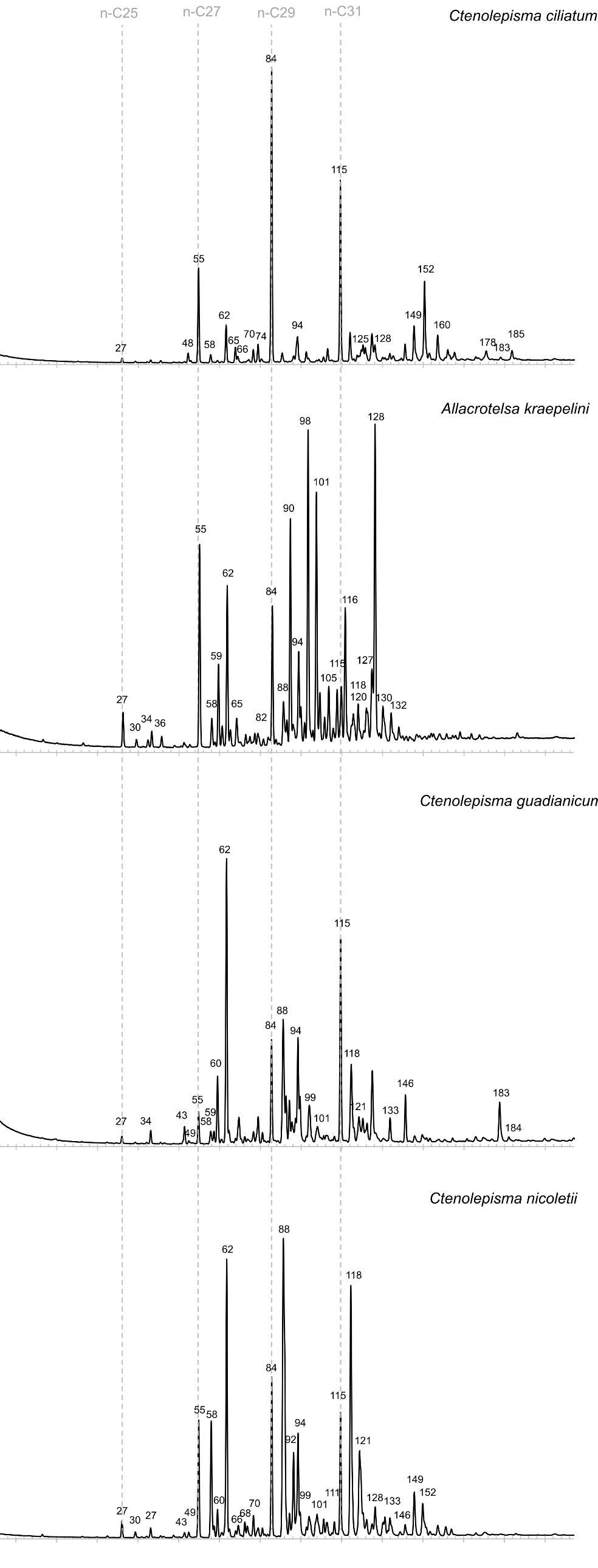

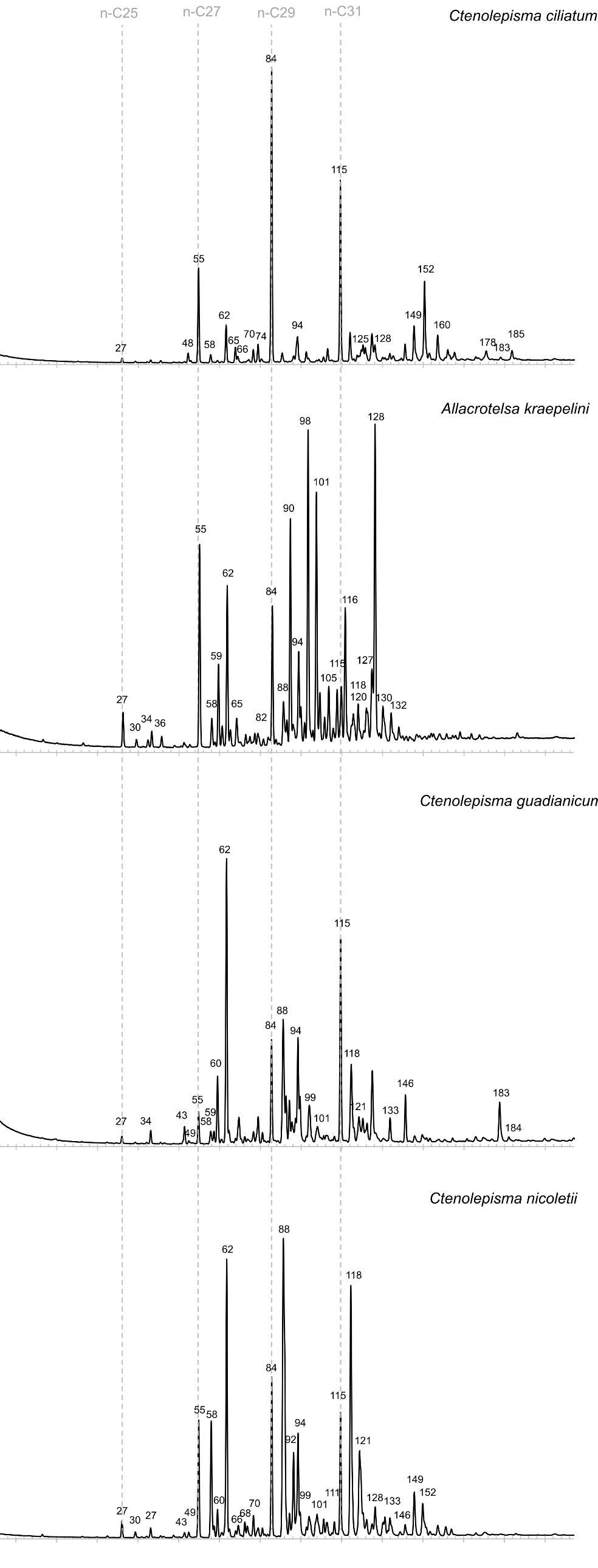
7
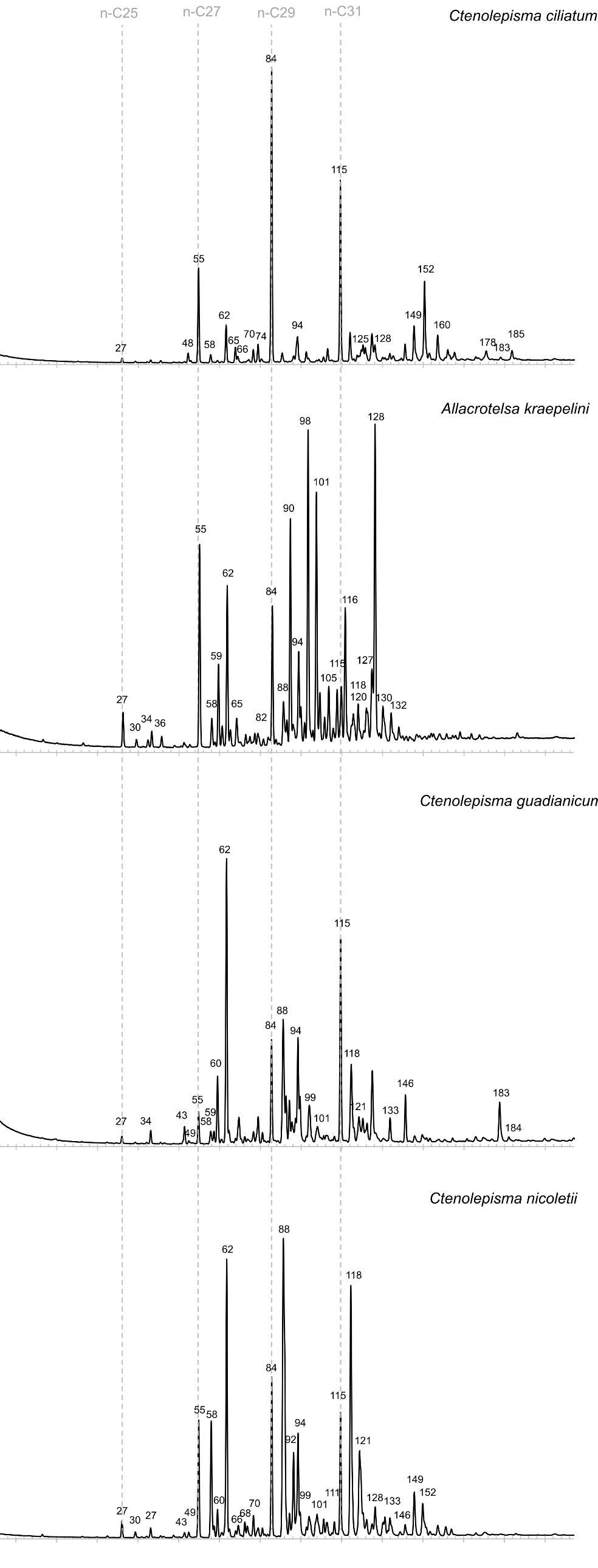
5

n-C27C29
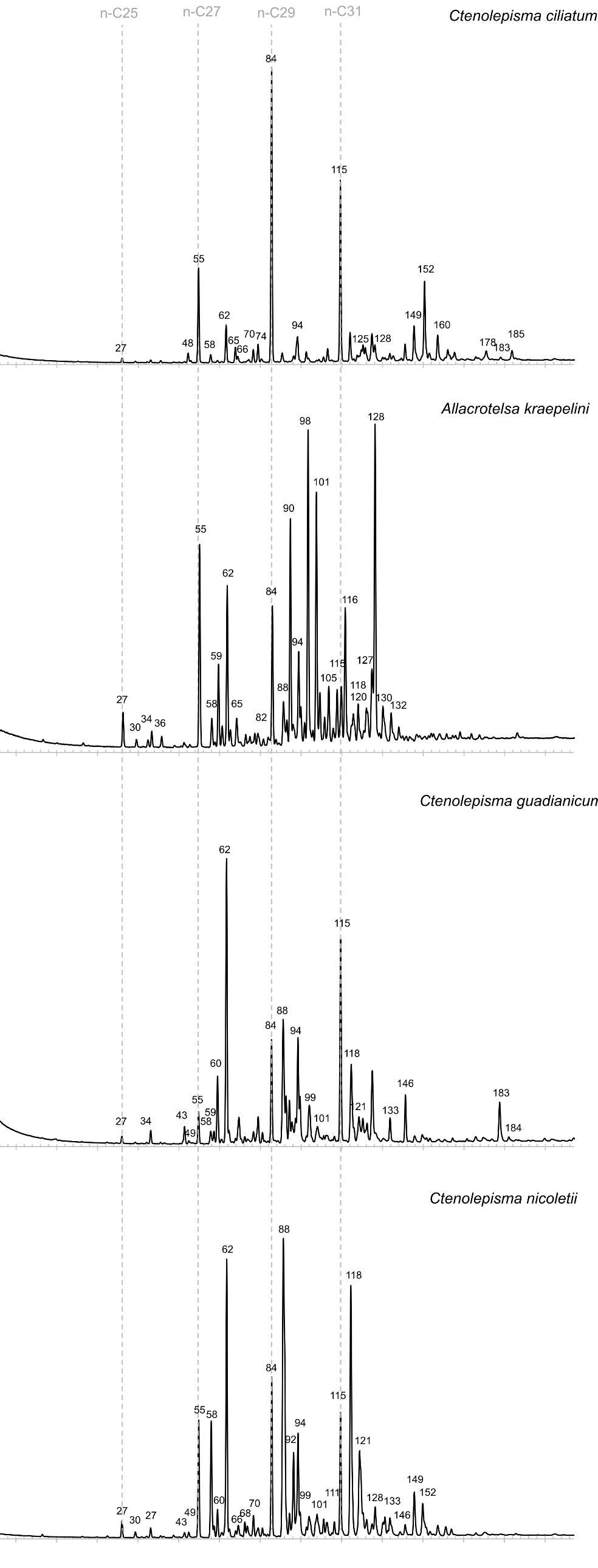

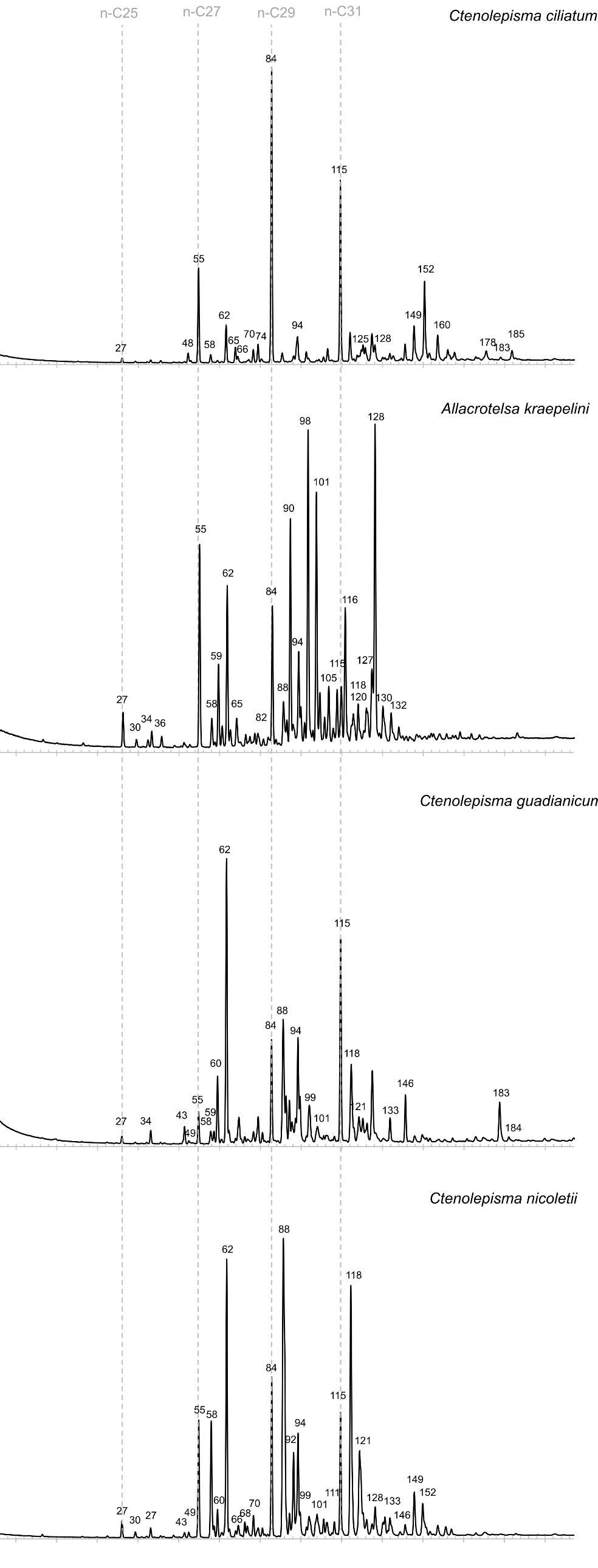
7
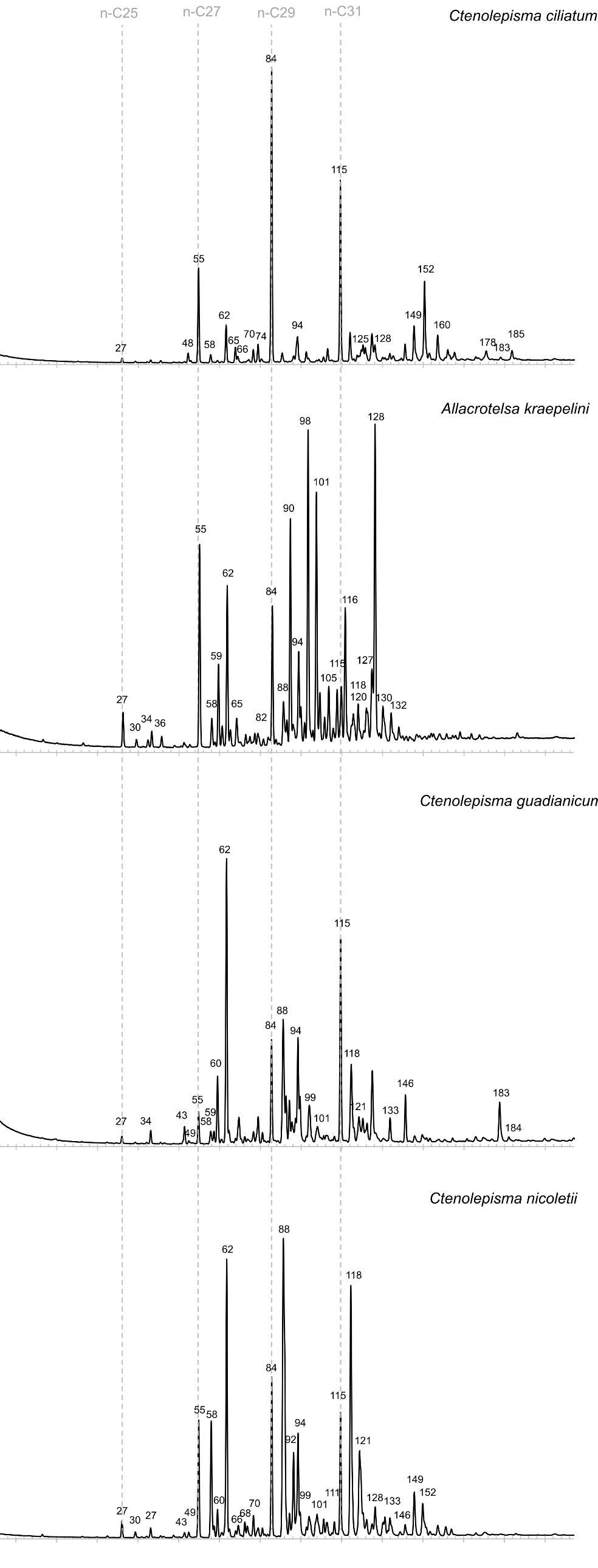
5

n-C25C29
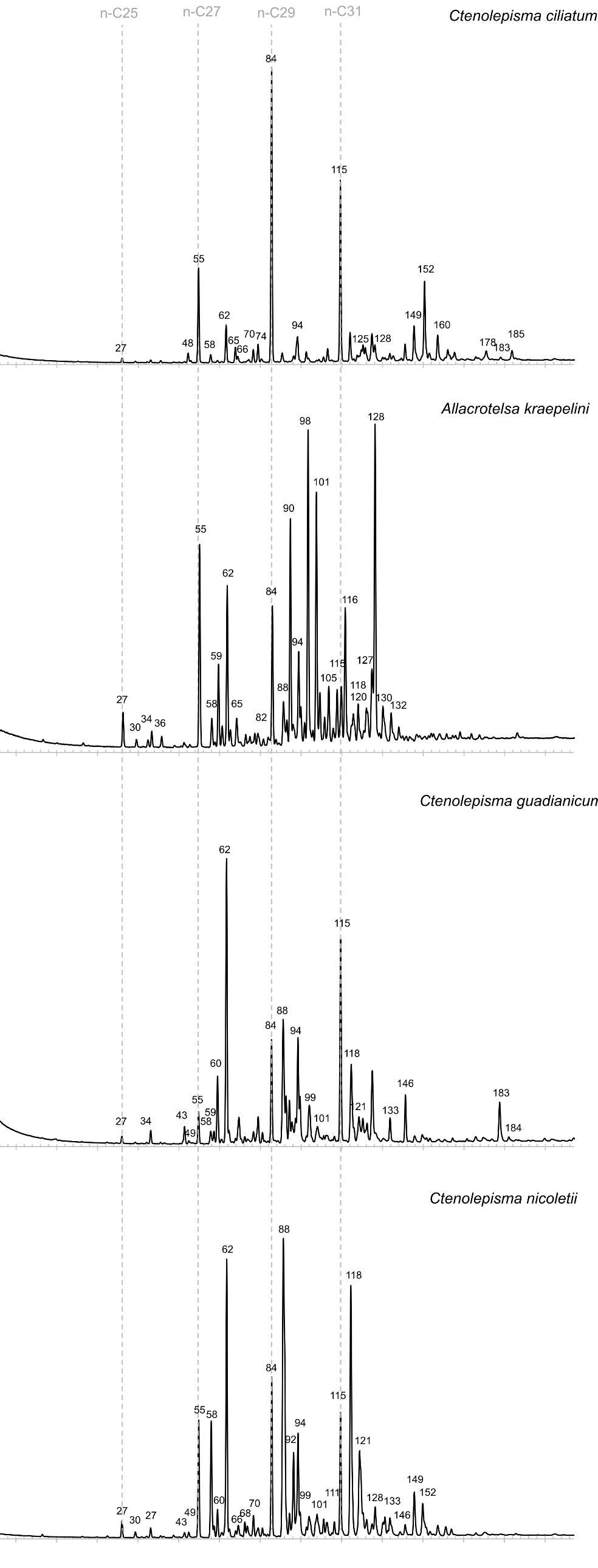

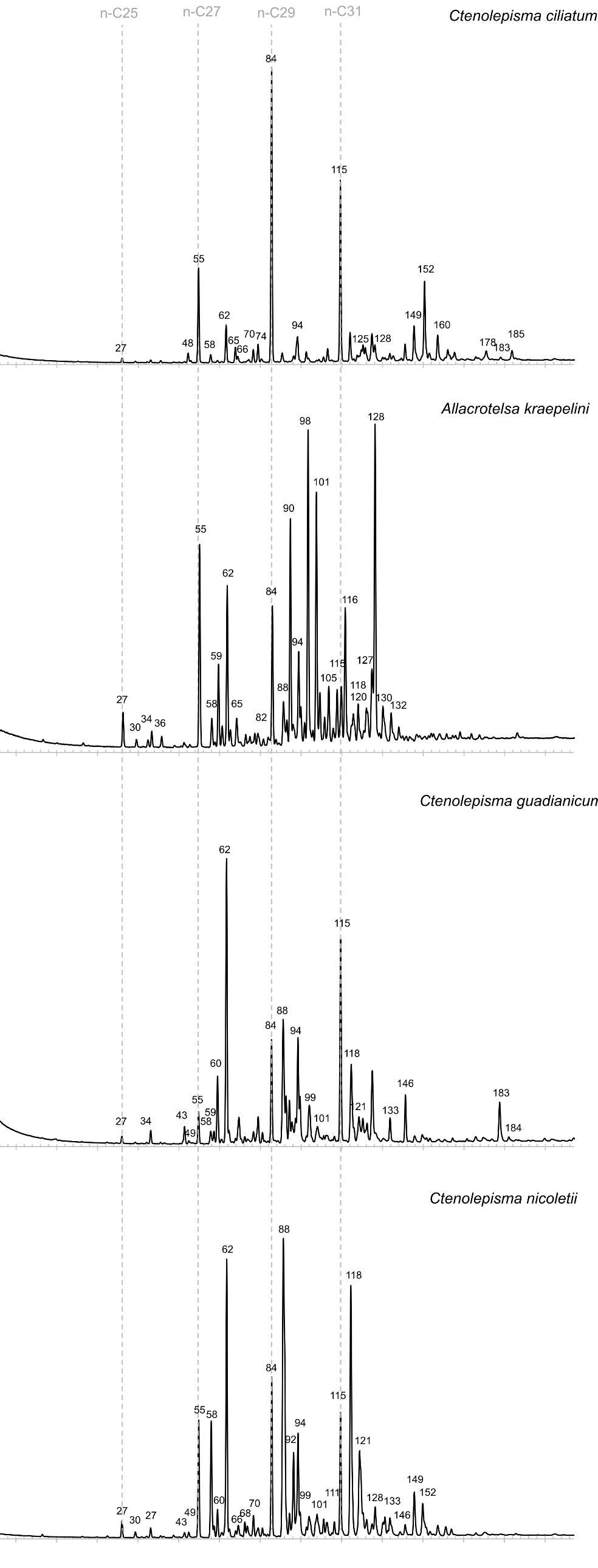
7
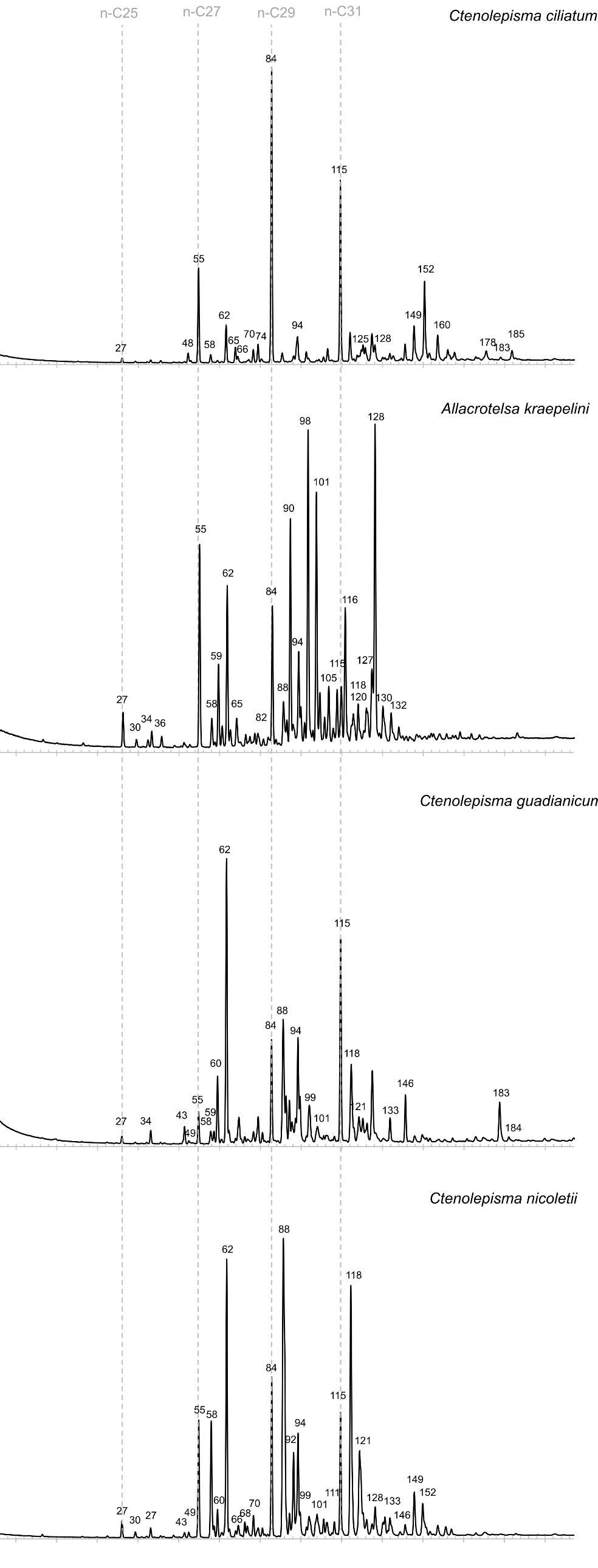
5

n-C29
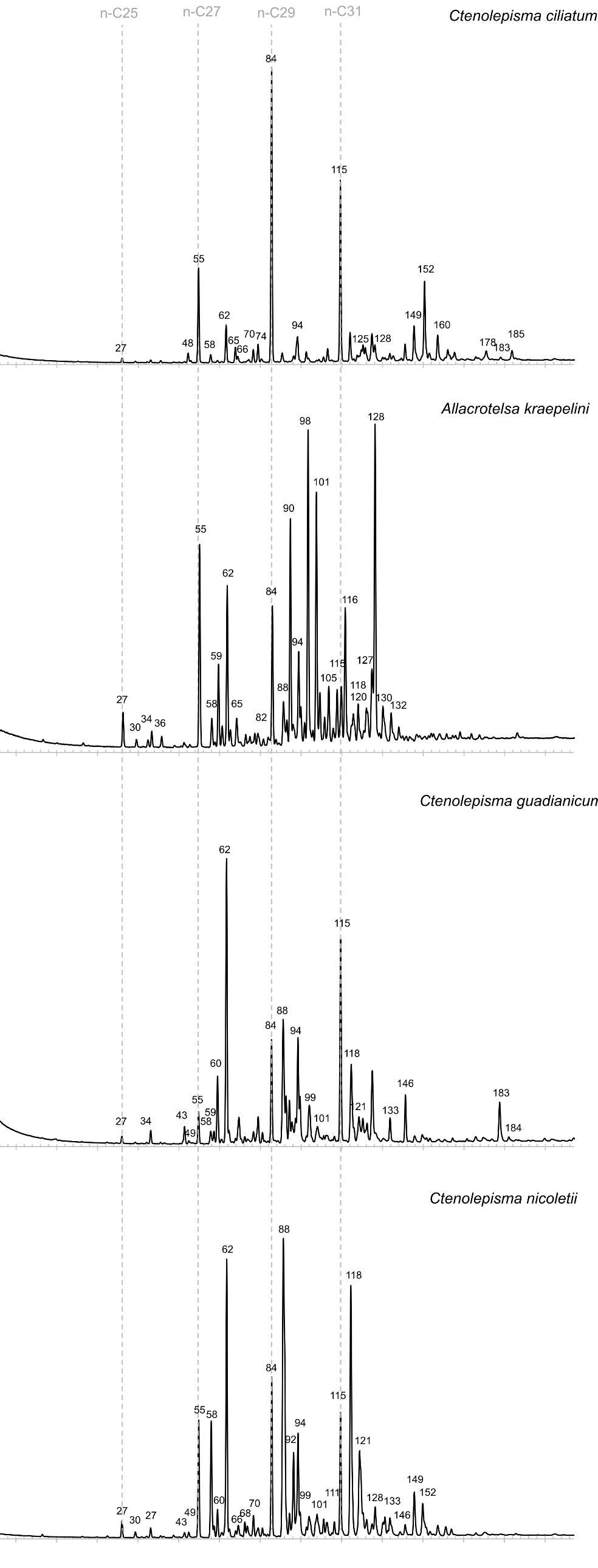

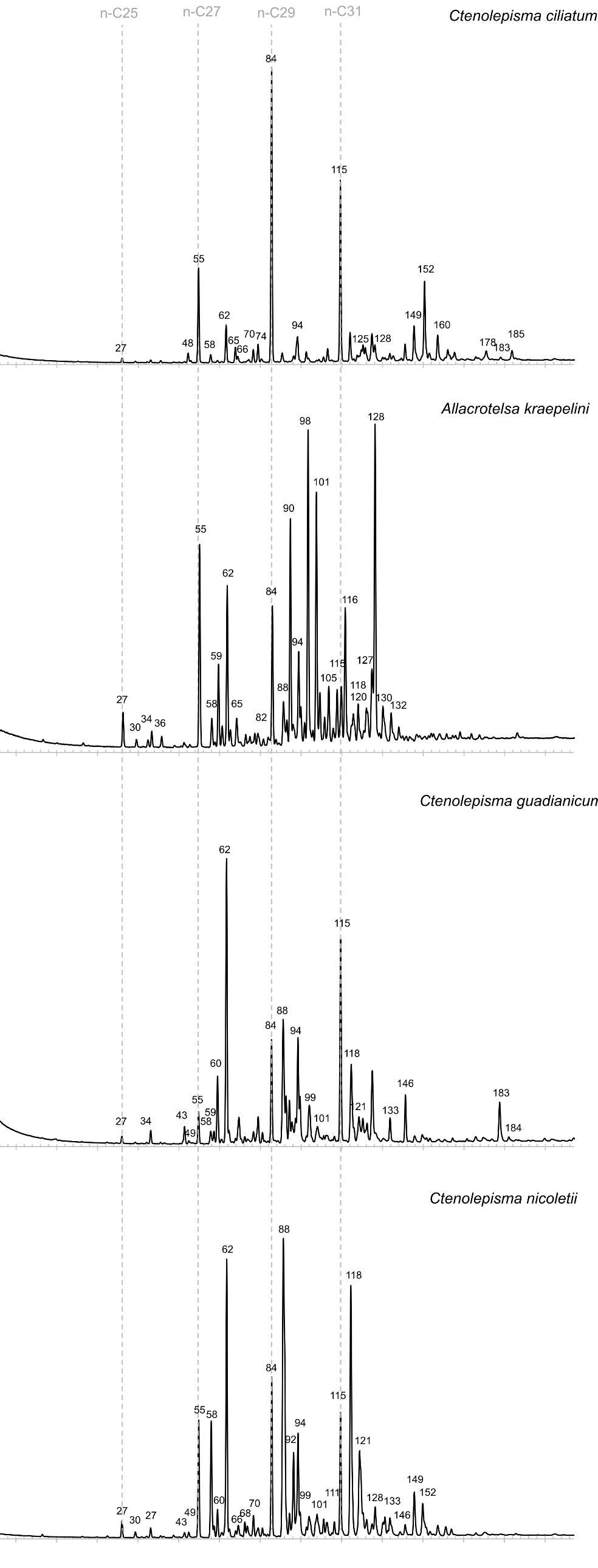
75

n-C3175

n-C27C2975

*Ctenolepisma nicoletii*

*Ctenolepisma guadianicum*
