# Supplementary material for: Chemical and behavioural strategies along the spectrum of host specificity in ant-associated silverfish
Source: BMC Zool. 2022 May 11;7:23. doi: 10.1186/s40850-022-00118-9 (PMC10127367; doi:10.1186/s40850-022-00118-9)
Supplement: Supplementary file 21 — Additional file 21. NMDS based on the CHC profiles of Messor and Messor specialists. [file 40850_2022_118_MOESM21_ESM.docx]

**Hierarchical clustering of the CHC profiles of ants and associated silverfish**

[CHC similarity among](https://www.researchgate.net/publication/7183430_Suzuki_R_Shimodaira_H_Pvclust_an_R_package_for_assessing_the_uncertainty_in_hierarchical_clustering_Bioinformatics_12_1540-1542) *[Messor](https://www.researchgate.net/publication/7183430_Suzuki_R_Shimodaira_H_Pvclust_an_R_package_for_assessing_the_uncertainty_in_hierarchical_clustering_Bioinformatics_12_1540-1542)* [specialists and their](https://www.researchgate.net/publication/7183430_Suzuki_R_Shimodaira_H_Pvclust_an_R_package_for_assessing_the_uncertainty_in_hierarchical_clustering_Bioinformatics_12_1540-1542) *[Messor](https://www.researchgate.net/publication/7183430_Suzuki_R_Shimodaira_H_Pvclust_an_R_package_for_assessing_the_uncertainty_in_hierarchical_clustering_Bioinformatics_12_1540-1542)* [host ants. NMDS plot displays the Bray-Curtis similarities for CHCs. Silverfish species are represented by different coloured circles around a letter code.](https://www.researchgate.net/publication/7183430_Suzuki_R_Shimodaira_H_Pvclust_an_R_package_for_assessing_the_uncertainty_in_hierarchical_clustering_Bioinformatics_12_1540-1542) *[Messor](https://www.researchgate.net/publication/7183430_Suzuki_R_Shimodaira_H_Pvclust_an_R_package_for_assessing_the_uncertainty_in_hierarchical_clustering_Bioinformatics_12_1540-1542)* [ants are depicted by a letter code without coloured circle (black letters:](https://www.researchgate.net/publication/7183430_Suzuki_R_Shimodaira_H_Pvclust_an_R_package_for_assessing_the_uncertainty_in_hierarchical_clustering_Bioinformatics_12_1540-1542) *[Messor barbarus](https://www.researchgate.net/publication/7183430_Suzuki_R_Shimodaira_H_Pvclust_an_R_package_for_assessing_the_uncertainty_in_hierarchical_clustering_Bioinformatics_12_1540-1542)*[, grey letters](https://www.researchgate.net/publication/7183430_Suzuki_R_Shimodaira_H_Pvclust_an_R_package_for_assessing_the_uncertainty_in_hierarchical_clustering_Bioinformatics_12_1540-1542) *[Messor timidus](https://www.researchgate.net/publication/7183430_Suzuki_R_Shimodaira_H_Pvclust_an_R_package_for_assessing_the_uncertainty_in_hierarchical_clustering_Bioinformatics_12_1540-1542)*[). The letter code refers to the host colony: A (S201), B (S202), C (S203), D (S204), E (S103), F (S104), G (S119), H (S120), I (S128), J (S129), K (S132), L (S133), M (S134), N (S135), O (S136), P (S138), R (S142), S (S145), T (S146), U (S149), V (S150), details of nests see Additional file 2.
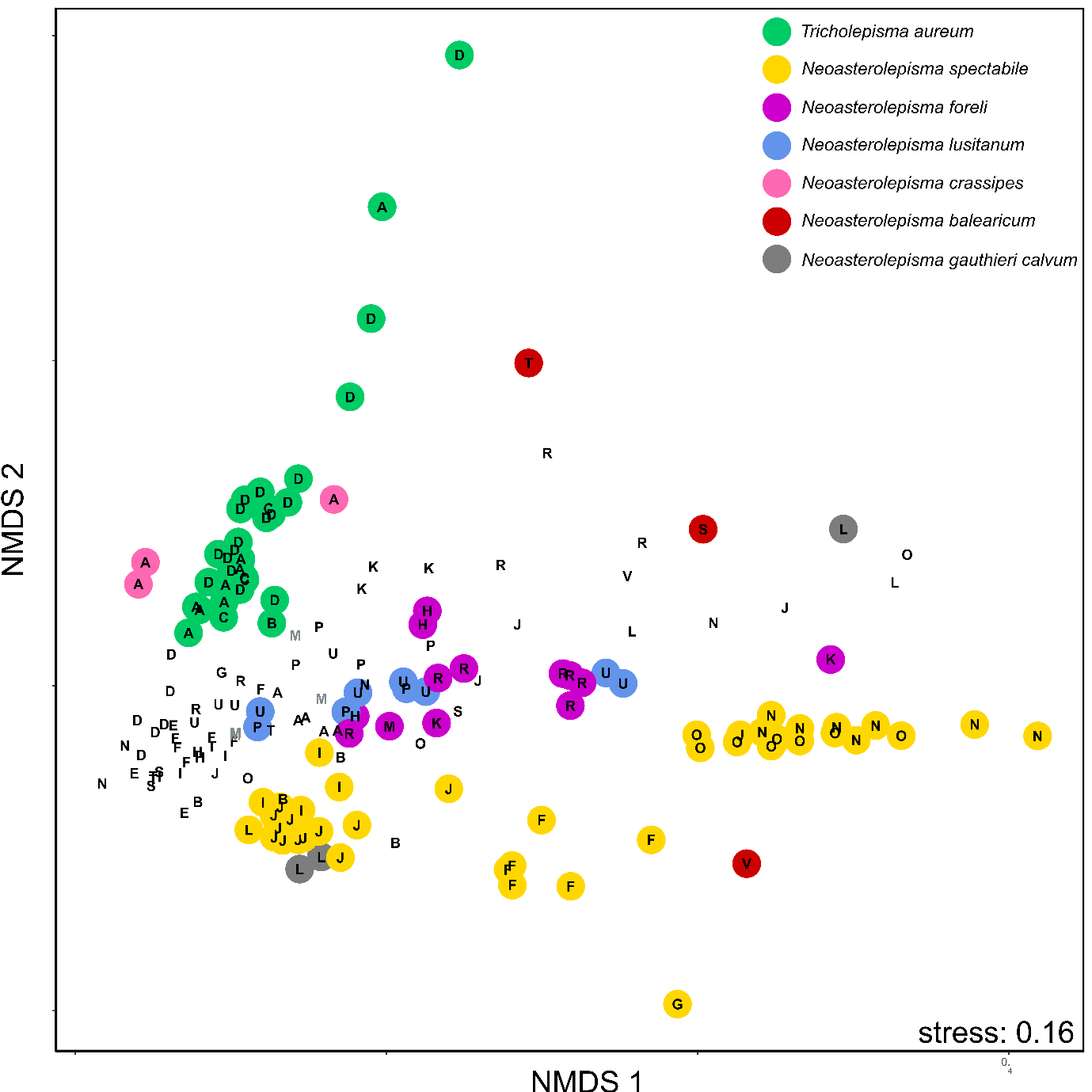
](https://www.researchgate.net/publication/7183430_Suzuki_R_Shimodaira_H_Pvclust_an_R_package_for_assessing_the_uncertainty_in_hierarchical_clustering_Bioinformatics_12_1540-1542)
